# Ring Strain Energies of Three-Membered Homoatomic Inorganic Rings El_3_ and Diheterotetreliranes El_2_Tt (Tt = C, Si, Ge): Accurate versus Additive Approaches

**DOI:** 10.1021/acs.inorgchem.2c01777

**Published:** 2022-08-24

**Authors:** Alicia Rey Planells, Arturo Espinosa Ferao

**Affiliations:** Departamento de Química Orgánica, Facultad de Química, Campus de Espinardo, Universidad de Murcia, 30100 Murcia, Spain

## Abstract

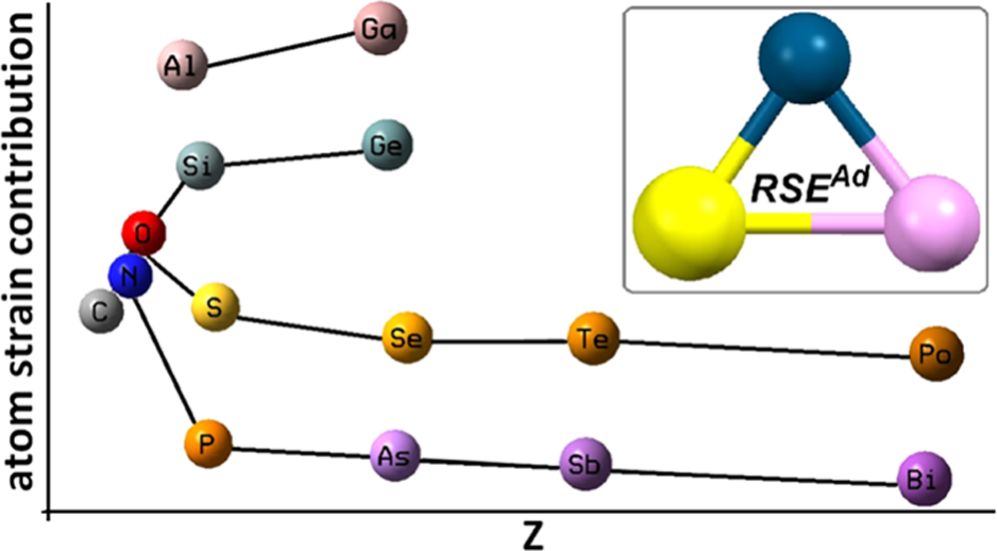

Accurate ring strain energies (RSEs) for three-membered symmetric inorganic rings El_3_ and organic dihetero-monocycles El_2_C and their silicon El_2_Si and germanium El_2_Ge analogues have been computed for group 14–16 “El” heteroatoms using appropriate homodesmotic reactions and calculated at the DLPNO-CCSD-(T)/def2-TZVPP//B3LYP-D4/def2-TZVP(ecp) level. Rings containing triels and Sn/Pb heteroatoms are studied as exceptions to the RSE calculation as they either do not constitute genuine rings or cannot use the general homodesmotic reaction scheme due to uncompensated interactions. Some remarkable concepts already related to the RSE such as aromaticity or strain relaxation by increasing the s-character in the lone pair (LP) of the group 15–16 elements are analyzed extensively. An appealing alternative procedure for the rapid estimation of RSEs using additive rules, based on contributions of ring atoms or endocyclic bonds, is disclosed.

## Abstract 2

Accurate ring strain energies (RSEs) are calculated for saturated three-membered homoatomic inorganic rings El_3_ and diheterotetreliranes El_2_Tt (Tt = C, Si, Ge). Aromaticity, lone-pair-induced strain relaxation for the group 15−16 elements, and bond stiffness are extensively discussed as main factors affecting the RSE. An attractive alternative procedure for fast estimation of the RSE using additive rules is proposed.

## Introduction

Ring strain energy (RSE) constitutes a remarkable parameter assessing the instability of small rings whose atoms acquire unfavorable high-energy bond angles and/or bond distances. This parameter provides information about the energy necessary for ring-enlargement or ring-opening transformations and even affecting other properties such as the stereochemical stability of σ^3^λ^3^-pnictogen ring atoms.^1^ It is also a useful parameter to explain electronic properties and reactivity in organic and inorganic ring systems, such as combustion processes of cyclopropane and cyclobutane,^2^ ring-opening metathesis polymerization (ROMP) processes,^3^ key reactions such as the ring-opening polymerizations (ROP) undergone by aziridines^4^ and other strained small rings (e.g., phosphiranes),^5^ NMR chemical changes in norbornanes,^6^ or changes in the hapticity of cyclopentadienyl rings in metallocenophanes.^7^ Not surprisingly, many computational studies have focused on how the RSE varies with the count of the ring members, the type of atoms forming the ring, and the nature of the peripheral substituents.^8^

The attractiveness of three-membered rings (3MRs) has prompted extensive studies on their synthesis and characterization for both organoheterocycles^9^ and purely inorganic rings. Among inorganic rings, kinetically stabilized tritetreliranes (R_2_El)_3_ (El = Si,^10^ Ge,^11^ and Sn^12^) have been reported, including X-ray structures in some cases. Also, tripnictogeniranes (REl)_3_ such as the lighter triaziridines^10j,13^ (El = N) and their heavier congeners triphosphiranes^10j,14^ (El = P) and triarsiranes^15^ (El = As) were described. It is worth mentioning that the important open-chain molecule ozone O_3_ has a less stable (ΔΔ*E* = 30.2 kcal/mol)^16^ cyclic allotrope (c-O_3_)^10j^ although heavier trichalcogeniranes are, to the best of our knowledge, so far experimentally unreported. Three-membered heterocycles possessing two identical heteroatoms “El” in addition to one carbon atom, referred to as CEl_2_ for short, have prominent examples in dioxiranes^17^ and the related oxaziridines^18^ that can be considered as epoxy derivatives of carbonyl compounds and imines, respectively, and are widely used as oxidizing reagents. As for the heavier heteroatomic tritetreliranes Tt_2_Tt′ (Tt, Tt′ = Si, Ge, Sn), the thermolysis of siladigermirane (SiGe_2_),^19^ as well as the isolation of stable adducts derived from the disilagermirane (Si_2_Ge)^20^ and disilastannirane (Si_2_Sn) rings, was reported.^21^ Moreover, [1+2] cycloaddition of the heavier carbene analogues, silylene (R_2_Si:) and germylene (R_2_Ge:), to a phosphaalkene leading to phosphasilirane and phosphagermirane, respectively,^22^ as well as the synthesis and properties of diphosphasiliranes^23^ and phosphadisiliranes, was also described.^24^ Three-membered rings containing silicon introduce interesting perspectives in the development of new silicon-containing polymeric materials that could exhibit interesting properties, in line with the unique characteristics of the well-known silicones, with many different uses such as in oils, greases, rubber-like materials,^25^ electric insulators,^26^ hydraulic fluids, and membranes^27^ among others. Rivard et al.^28^ recently reviewed the small (three- and four-membered) inorganic rings (i.e., containing no C as ring atom) described to date, which open up a range of interesting possibilities with potential yet to be discovered. Noteworthy is that only 30 types of inorganic rings and 40 organic rings (containing at least one C atom) have been reported so far. Considering that there are only 20 different atoms among the main group elements (groups 13–16) from second to sixth rows, it is possible to obtain 1540 combinations with repetition of the different elements in three-membered rings. In other words, only around 4.5% of the rings have been reported so far, and not all possible applications have been exhausted for them. This is therefore an exciting field still to be explored. Moreover, RSE estimates were reported for only a very limited number of organoheterocycles. Recently, RSE data were published for the parent rings (CH_2_)_2_X, where X are group 13–16 elements (El) in their lowest oxidation state.^29^ The RSE for parent heterocycles C_2_O, C_2_S, C_2_N, C_2_P and disilaanalogues Si_2_El (El = C, N, O, Si, P, S),^30^ CPN,^31^ CPO,^32^ and CPS^33^ (using only ring atoms to refer to each ring type) and of inorganic ditetreliranes Si_2_El (El = Al, Si, P)^34^ and Ge_2_El (El = Ga, Ge, As)^35^ as well were reported. There is therefore an important gap with respect to the systematic evaluation of RSE for a wide range of small rings.

**Figure 1:**
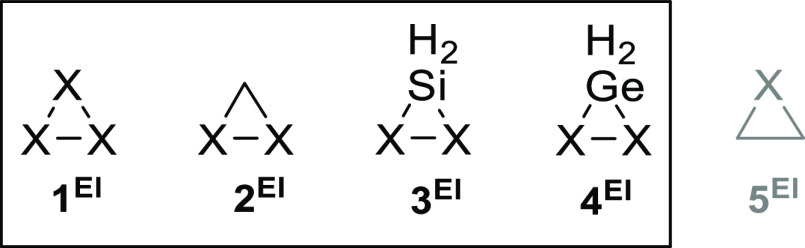
Three-membered heterocycles herein (**1**–**4**) and previously (**5**) studied. X is a group 13–16 element (El) with its typical covalency completed with bonds to H.

For this purpose, the RSE of a set of 54 3MRs are herein reported for symmetric (homocyclic) inorganic rings El_3_ (**1**), organoheterocycles with two identical heteroatoms CEl_2_ (**2**), and the heavier analogues SiEl_2_ (**3**) and GeEl_2_ (**4**), where El is an element belonging to groups 13–16 with its typical covalency (3, 4, 3, and 2 for groups 13, 14, 15, and 16, respectively) completed with bonds to hydrogen (i.e., parent rings) (Figure 1). Data corresponding to the already recently reported C_2_El rings^29^ (**5**) were also obtained to analyze possible trends in the factors affecting the ring strain.

The interesting question of whether it is possible to determine how much strain each ring bond or atom contributes to the total RSE and if this can be calculated additively, is also addressed.

## Results and Discussion

### Accurate RSEs—Scope and Limitations

**Scheme 1:**
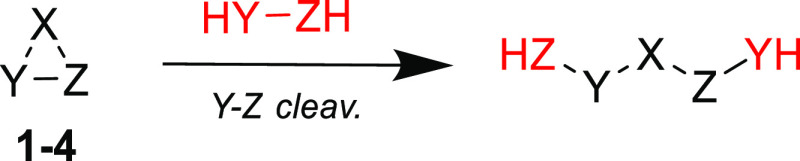
One of the Three Possible Homodesmotic (RC4) Ring-Opening Reactions Used for the Estimation of RSEs for **1**–**4**^**El**^

RSEs of all 3MRs herein investigated were calculated using suitable homodesmotic reactions (reaction class 4, or “RC4”). The latter are the second-best type in a hierarchy of increasingly accurate processes, due to the conservation of larger fragments, according to a recent classification and redefinition of the reaction types used in thermochemistry.^36^ The homodesmotic reactions used (Scheme 1) to evaluate RSEs were shown^29^ to be sufficiently accurate compared to the formally highest ranked, hyper-homodesmotic reactions, that have the disadvantage of being prone to unwanted interactions present in longer chains. The RSE was obtained as the average of the opposite of energetics (including zero-point energy correction) for the three endocyclic bond-cleavage reactions in each case, at the DLPNO-CCSD(T)/def2-TZVPP(ecp)//B3LYP-D4/def2-TZVP(ecp) level (see computational details) (Table 1).

**Table 1:** Calculated (DLPNO-CCSD(T)/def2-TZVPP(ecp)) RSE Values (kcal/mol) for Compounds **1**^**El**^, **2**^**El**^, **3**^**El**^, and **4**^**El**^

**1^Tl^**	1.85^a^	**2^Tl^**		**3^Tl^**		**4^Tl^**	
**1^C^**(10j,^29^,^37^)	27.27	**2^C^**	27.27	**3^C^**(24)	36.32	**4^C^**(24)	36.97
**1^Si^**(10j,37b,^38^)	36.09	**2^Si^**(37b,^38^)	39.32	**3^Si^**	36.09	**4^Si^**	37.09
**1^Ge^**(35)	38.70	**2^Ge^**	40.67	**3^Ge^**	37.95	**4^Ge^**	38.70
**1^Sn^**	36.29^a^	**2^Sn^**	39.12	**3^Sn^**	35.89	**4^Sn^**	36.67^a^
**1^Pb^**	15.22^b^	**2^Pb^**		**3^Pb^**		**4^Pb^**	
**1^N^**(10j)	13.96	**2^N^**	21.63	**3^N^**	41.59	**4^N^**	37.76
**1^P^**(10j)	6.06	**2^P^**	12.24	**3^P^**	17.02	**4^P^**	18.35
**1^As^**	5.74	**2^As^**	12.04	**3^As^**	15.83	**4^As^**	17.03
**1^Sb^**	5.77	**2^Sb^**	12.10	**3^Sb^**	14.21	**4^Sb^**	15.36
**1^Bi^**	6.34	**2^Bi^**	10.01	**3^Bi^**	12.41	**4^Bi^**	13.49
**1^O^**(10j)	28.42	**2^O^**	21.40	**3^O^**	33.78	**4^O^**	35.52
**1^S^**(10j)	28.77	**2^S^**	19.36	**3^S^**	22.44	**4^S^**	24.67
**1^Se^**	25.71	**2^Se^**	18.87	**3^Se^**	20.33	**4^Se^**	22.20
**1^Te^**	26.52	**2^Te^**	20.49	**3^Te^**	19.86	**4^Te^**	21.71
**1^Po^**	24.98	**2^Po^**	19.62	**3^Po^**	18.55	**4^Po^**	20.14

a Calculated according to Scheme 1.

b Calculated as the average between the RSEs resulting from reactions in Scheme 2.

In case of the homocyclic inorganic rings, **1**^**El**^, no energy minimum was found for group 13 elements. Instead, a variety of previously reported El_3_H_3_ hydride isomers were obtained (El = B,^39^ Al, Ga^39,40^), whose interest stems from their potential use as reversible hydrogen carrier devices at low and medium temperatures^41^ and formation of clusters.^42^ El_3_H_3_ hydrides for the heaviest triels In and Tl have not been previously reported and their calculated structures **I–VIII** are collected together with the corresponding relative energies (Figure S1). As they do not contain three identical ElH units (structure **IV**^**Tl**^ being an exception, *vide infra*), no appropriate homodesmotic reactions were found that could allow the estimation of their RSE (Scheme 1).

**Figure 2:**
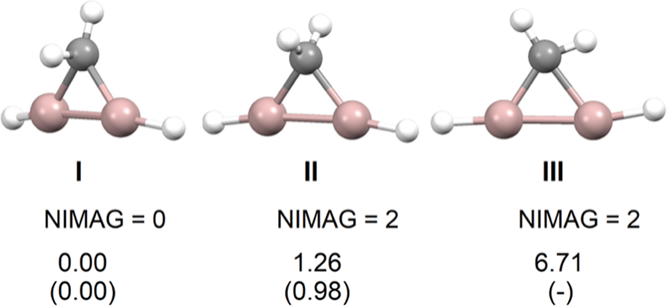
Optimized structures for the distorted tetrahedral (**I**), tetrahedral *C*_2*v*_ (**II**), and planar *C*_*s*_ (**III**) **2**^**Al**^ isomers, number of imaginary frequencies (NIMAG), and their respective relative ZPE-corrected energies (kcal/mol) for **2**^**El**^ (El: Al, Ga) isomers (values for E = Ga in parenthesis).

With regard to triel-containing dihetero-monocyclic species **2**^**El**^, it is known that the incorporation of boron in small rings stabilizes the planar tetracoordination at carbon by π delocalization, in addition to less important σ donor effects.^43^ The diboracyclopropane (diborirane) **2**^**B**^*C*_2*v*_ conformer with tetrahedral C atom and its planar *C*_*s*_ conformer were reported to be second- and first-order saddle points, respectively, at the B3LYP/6-311+G* level. The energy minimum resulted to be a fully planar cyclic structure with a two-electron three-center B–H–B bond. However, among cyclic structures with the required connectivity (containing a CH_2_ group), the most stable one contains an asymmetric planar tetracoordinated carbon atom featuring different endocyclic C–B bond distances.^44^ The corresponding unreported heavier cyclic analogues with the appropriate cyclic −TrH–TrH–CH_2_– connectivity, for Tr = Al (**2**^**Al**^) and Ga (**2**^**Ga**^), show a distorted tetrahedral (i.e., nonplanar) geometry at C for the energy minima (**I**), and two other second-order saddle point cyclic structures with increasing energy for tetrahedral *C*_2*v*_ (**II**) and planar *C*_*s*_ environments at C (**III**), respectively (Figure 2). It seems clear that, unlike B, the heavier triels do not favor planar tetrahedral geometries at the adjacent C atom. Not only in the abovementioned triel-containing **2**^**El**^ but also in **3**^**El**^ and **4**^**El**^ rings, the lack of undistorted cyclic minima precluded the design of suitable homodesmotic reactions considering these effects. Accordingly, these rings were excluded from the RSE calculations.

**Figure 3:**
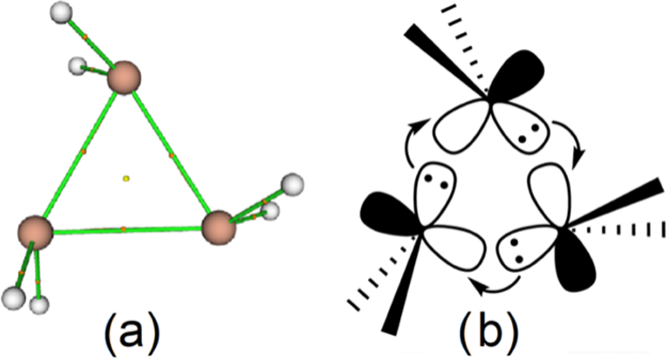
Computed (B3LYP/def2-TZVPP) BCP (small orange spheres), RCP (small yellow sphere), and bond paths for **1**^**Pb**^; sketched representation of El–El bonds for **1**^**Sn**^ and **1**^**Pb**^.

On the other hand, a triplumbirane, c-(PbR_2_)_3_ (R = 2,4,6-triethylphenyl), was isolated in 2003.^45^ The parent **1**^**Pb**^ species was shown to display a cyclic character, featuring elongated bond distances of ∼3.231 Å together with substituent twisting of ca. 50° outside ideal conditions (H–Pb–H plane bisecting the endocyclic bond angle) calculated at the HF/DZ(d) level. This reveals that the Pb–Pb bonding is characterized by donor–acceptor interactions and not arising from overlapping of identical atomic orbitals (AOs). Thus, the Pb–Pb bonds formally result from the interaction of plumbylene (R_2_Pb:) lone pairs with the empty p AO at P of one of the neighboring plumbylene units, thus forcing the plumbylene substituents to twist from their ideal (untwisted) position to maximize overlap (Figure 2b).^46^ At the working level of theory (B3LYP/def2-TZVPP(ecp)//B3LYP-D4/def2-TZVP(ecp)), the parent compound **1**^**Pb**^ presents a ring critical point (RCP) (Figure 3a) and the natural bond orbital^47^ (NBO) analysis reveals that each Pb–Pb bond is rather elongated and weak (*d*_Pb–Pb_ = 3.137 Å; WBI_Pb–Pb_ = 0.547) compared to related acyclic species (*d*_Pb–Pb_ = 2.895 Å; WBI_Pb–Pb_ = 0.854 for model H_3_Pb–PbH_3_). The endocyclic Pb–Pb linkage in **1**^**Pb**^ seems to be mostly formed as a donor–acceptor bond from a filled roughly sp^2^ AO at one Pb atom (Pb1) to an empty almost pure p orbital at the second Pb atom (Pb2), with remarkable SOPT (second-order perturbation theory) electron donation to the σ*(Pb1–Pb3) (*E*_SOPT_ = 24.2 kcal/mol) and the two σ*(Pb1–H) molecular orbitals (MOs) (*E*_SOPT_ = 29.4 kcal/mol each). The unsymmetrically located bond critical point (BCP), closer to the acceptor Pb2 atom, also supports this view (*d*_Pb1-BCP_ = 1.634 Å; *d*_Pb2-BCP_ = 1.503 Å), displaying an almost vanishing value for the Laplacian of the electron density at the BCP (∇^2^ρ = 0.0389 au) as one of the characteristic features of dative bonding.^48^ However, it was not possible to differentiate the two expected valence–shell concentration bands (VSCC) corresponding to the donor and acceptor atoms at the central part of the ∇^2^ρ function along the Pb–Pb path. Instead, only one broad band (superposition of two individual and similar VSCC_Pb_ bands), mostly located within the basin of the donor atom, as additional signature of dative bonding,^48^ could be observed (Figure S2). The same type of nonsymmetric donor–acceptor tetrel–tetrel bonds with twisted substituents were found in **1**^**Sn**^.

For these two rings **1**^**Sn**^ and **1**^**Pb**^, the general homodesmotic reactions used for all other rings (Scheme 1) should not be used as it is not possible to compensate for this type of interactions in the open-chain ring-cleavage products, where donor–acceptor El→El bonds are not present. Therefore, different hyper-homodesmotic reactions were used (Scheme 2) taking advantage of the presence of donor–acceptor Pb→Pb bonds with twisted substituents in five- and six-membered rings (PbH_2_)*_n_* (*n* = 5, 6) and assuming negligible ring strain in these moderate-sized homocyclic rings. The RSE for **1**^**Pb**^ was obtained as the average of both RC5 reactions (per **1**^**Pb**^ unit). However, the H substituents are not twisted in the Sn-containing five- and six-membered homocyclic rings, which let the effect of the donor–acceptor bonds in **1**^**Sn**^ uncompensated. Similarly, **1**^**Tl**^ (structure **IV**^**Tl**^, Figure S1) displays a twisted geometry corresponding to three donor–acceptor endocyclic Tl→Tl bonds, but larger sized (TlH)*_n_* rings (*n* = 5, 6) are not stable. Therefore, the corresponding obtained RSE values for both **1**^**Sn**^ and **1**^**Tl**^ (Table 1) were calculated according to Scheme 1 and should be taken with caution.

**Scheme 2:**
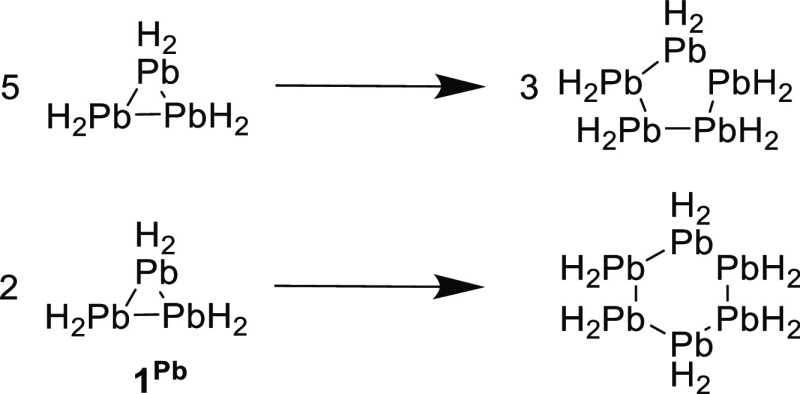
Hyper-homodesmotic (RC5) Reactions Used for the Estimation of RSE for **1**^**Pb**^

A less pronounced substituent twist can be observed in **4**^**Sn**^ (Figure S3). In this case, since the Sn–Sn bond is not symmetrical, neither are the two Ge–Sn bonds which have slightly different bond distances (*d*_Ge–Sn1_ = 2.655 Å, WBI_Ge–Sn1_ = 0.942; *d*_Ge–Sn2_ = 2.662 Å, WBI_Ge–Sn2_ = 0.922). This results from a different p-character at Ge and Sn being 85.2 and 72.8%, respectively, in the first case, and reversed to 74.7 and 84.3% in the second one. Hence, due to uncompensated interactions in the homodesmotic cleavage reactions, the computed RSE for **4**^**Sn**^ should also be taken with caution.

The other lead-containing rings **2**^**Pb**^, **3**^**Pb**^, and **4**^**Pb**^ do not exist as minima and **5**^**Pb**^ was already reported^29^ as a pseudocyclic Dewar–Chatt–Duncanson-type structure.^49^

It is known that Se_3_ and Te_3_ show a slight preference for the cyclic *D*_3*h*_ over the acyclic *C*_2*v*_ (bent) geometry, which is the most stable isomer for the lighter chalcogens O and S.^50^ At the working level of theory, the same cyclic preference is observed for the heaviest Po_3_ and new linear species *D*_∞*h*_ were found as most unstable isomers for O_3_ and Te_3_, whereas *D*_∞h_-Po_3_ is a transition state between two degenerated C_2*v*_ structures (see Table S3 for relative energies of trichalcogen isomers).

### Factors Affecting RSE

**Figure 4:**
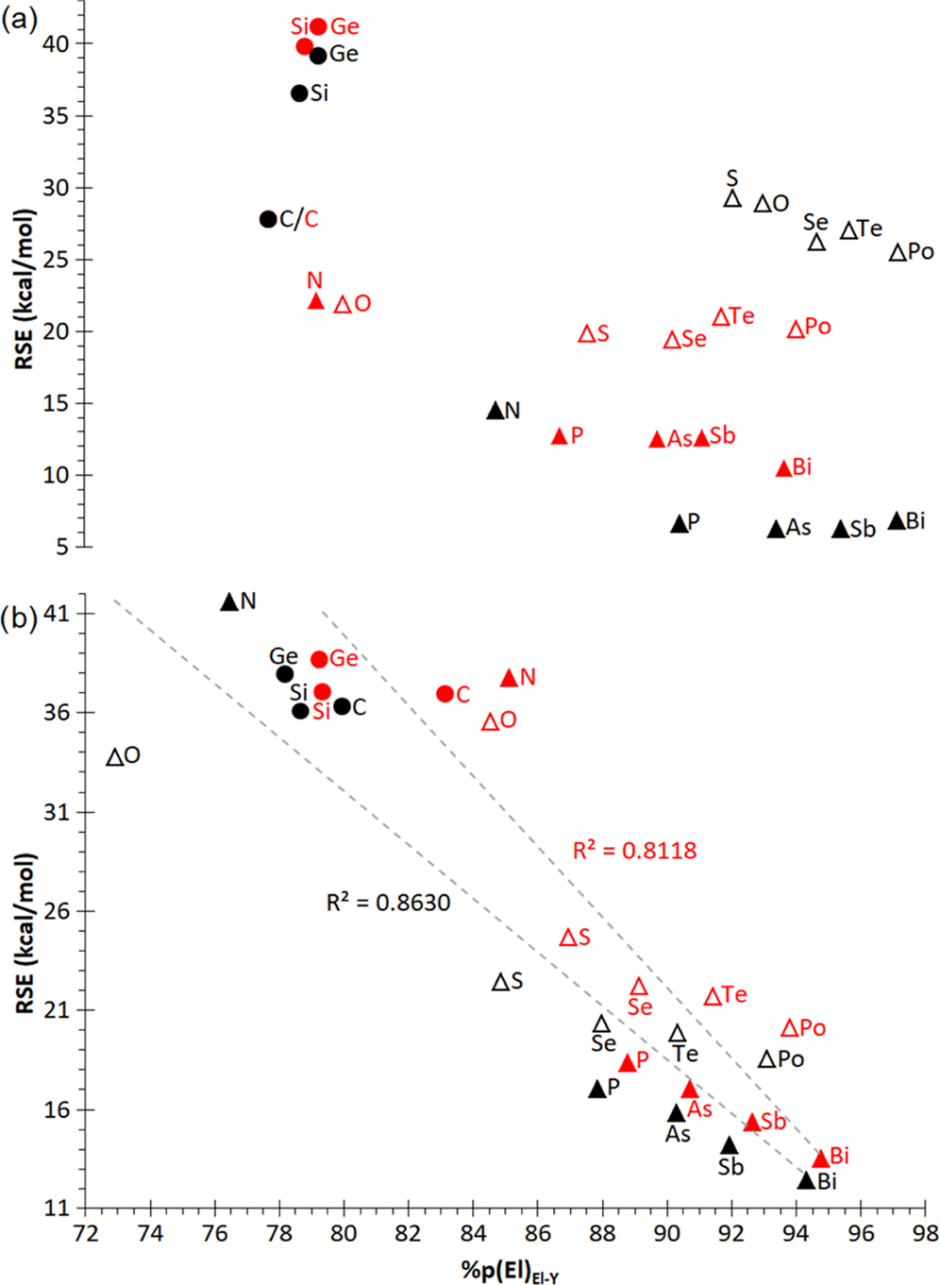
Plots of RSE vs p-character of the AO used by the heteroatom El for its endocyclic El–Y bonds in El_2_Y rings: (a) **1**^**El**^ (Y: El, black), **2**^**El**^ (Y: C, red), (b) **3**^**El**^ (Y: Si, black), and **4**^**El**^ (Y: Ge, red).

As observed for monoheteroatomic saturated^29^ and unsaturated^51^ 3MRs, one of the main mechanisms of strain relaxation is the effect of lone electron pair (LP) hybridization. According to this effect, an increase in the s-character of the LP-containing AO enhances the p-character of the AOs used by El in the endocyclic bonds, giving rise to a sp*^n^*-type hybridization with *n* > 3 (“high” p-character, beyond 75%) which fits better to small endocyclic bond angles (Figure 4).

Rings containing a tetrel atom as heteroelement El lack LPs, thus preventing the corresponding strain relaxation mechanism, which makes them generally the most strained rings for all four ring types **1**–**4**^**El**^. In the case of pnictogen- and chalcogen-containing rings, there is a clear ring strain relaxation from the second to the third row in the **1**^**El**^ and **2**^**El**^ rings (excepting **1**^**O**^), although it remains roughly constant for the heaviest elements (Figure 4a). In turn, for the **3**^**El**^ and **4**^**El**^ rings (Figure 4b), the linear correlation between the increase of the p-character in El for the Si/Ge–El bond and the RSE relaxation is quite remarkable, as already reported for the case of the monohetero-monocyclic rings **5**^**El**^ (which also included group 13 heteroelements). It is important to note that **3**^**N**^ and **3**^**O**^ present RSE well beyond the heaviest congeners and in the range of group 14 rings. On the other hand, among the four types of rings studied, the least strained are homoatomic **1**^**El**^ rings, while the ring strain slightly increases on changing one El atom to C (**2**^**El**^), Si (**3**^**El**^), and Ge (**4**^**El**^).

The RSE also shows some correlation with the s-character of the LP-containing AO at the El heteroatom for groups 15 and 16 (Figure S4 and Table S1), although it does not vary significantly for the heaviest group 15 **1**^**El**^-type rings.

**Figure 5:**
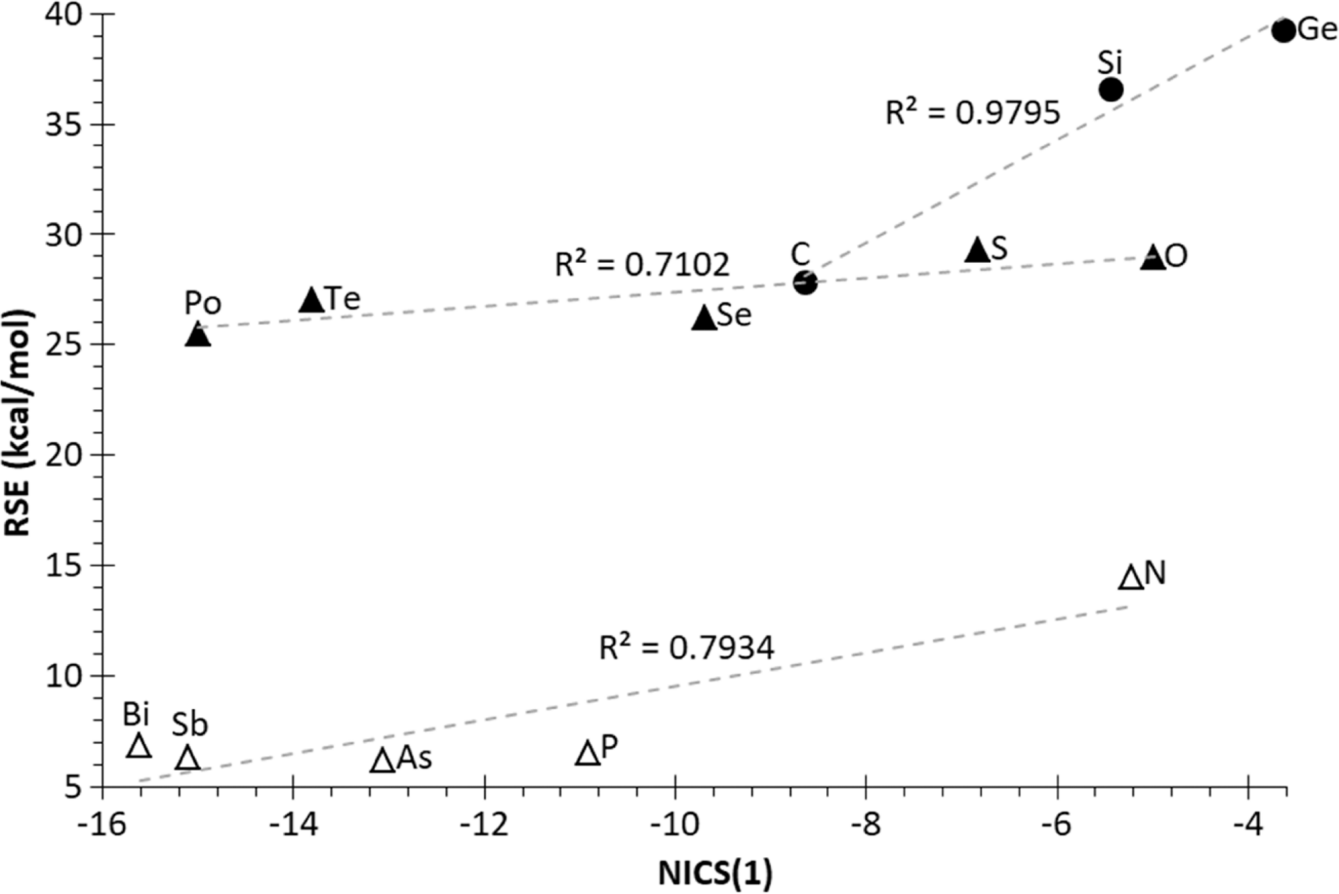
Plot of RSE vs NICS(1) for **1**^**El**^.

The unusual stability of cyclopropane **1**^**C**^, with a RSE (27.5 kcal/mol) very close to that of cyclobutane (26.5 kcal/mol), contrasts with its silicon counterpart trisilacyclopropane (trisilirane, **1**^**Si**^) whose RSE is beyond twice that of tetrasilacyclobutane.^52^ This, along with the exhibition of olefin-like properties, such as the formation of metal complexes or undergoing catalytic hydrogenation,^53^ has led to numerous studies attributing σ-aromaticity as the cause of this stabilization.^54^ However, based on “extracyclic resonance energy” (ECRE) calculations, the σ-aromaticity stabilization for **1**^**C**^ has been quantified in only 3.5 kcal/mol.^55^ Nucleus-independent chemical shifts (NICS)^56^ were reported to quantify aromaticity, so that the more negative the NICS value, the higher the aromatic character. The analysis of NICS values for a complete set of three- to six-membered inorganic rings derived from the main group elements unveils that the ring σ-(anti)aromaticity is mostly due to interactions among electrons making up the endocyclic El–El bonds rather than the El–H bonds or LPs.^57^ This study also points out the fact that the simple counting of electrons in endocyclic bonds follows Hückel’s rule so that the saturated three- and five-membered rings (4n+2 electrons) are aromatic whereas four- and six-membered rings (4n electrons) are antiaromatic. NICS analysis using localized molecular orbitals suggested that the σ-ring electrons of trichalcogeniranes **1**^**El**^ (El = O, S, Se, Te, Po) are chiefly responsible for their aromaticity.^57^ Moreover, a NICS variation study along the *z-*axis perpendicular to the ring plane revealed double (σ + π) aromaticity in heavier trichalcogeniranes, the induced diatropic ring current arising from T_*x*,*y*_-allowed transitions involving both σ- (a_1_′ → e′) and π-type (a_2_″ → e″) molecular orbitals (MOs).^58^ The overall excitation energies were shown to decrease (aromaticity increase) on descending the group, with the π-aromaticity prevailing over the σ-component only in the case of S and Se. NICS(1) values were calculated for all herein studied **1**^**El**^ rings at the working level of theory, being in line with those already reported (root-mean-square error RMSE = 0.657 ppm). For group 14 **1**^**El**^ rings, the increase (less negative) in NICS(1) values on descending the group entails a destabilization of the rings (higher RSE) (Figure 5). The decrease in aromaticity might be related to poorer spatial overlap among σ(El–H) MOs at both sides of the rings due to the increasing ring size (Figure S5). However, for group 15–16 elements, a remarkable increasing aromaticity (more negative NICS(1) values) on descending the groups does not significantly affect the RSE (except for N). This could be the result of the counteracting additional effect of a simultaneous increase in the flexibility of endocyclic bond angles (*vide infra*).

To try to explain this effect, the highest occupied molecular orbital–least unoccupied molecular orbital (HOMO–LUMO) gap, Δε_H–L_, was studied. It has been used as an indicator of the kinetic stability of a compound so that a low-lying HOMO would indicate a difficult electron extraction and a high LUMO suggesting the unfavorable addition of electrons.^59^ This Δε_H–L_ represents the chemical hardness (η) of a molecule according to Pearson,^60^ large band gaps corresponding to stable structures, which is the normal situation for classical aromatics.

**Figure 6:**
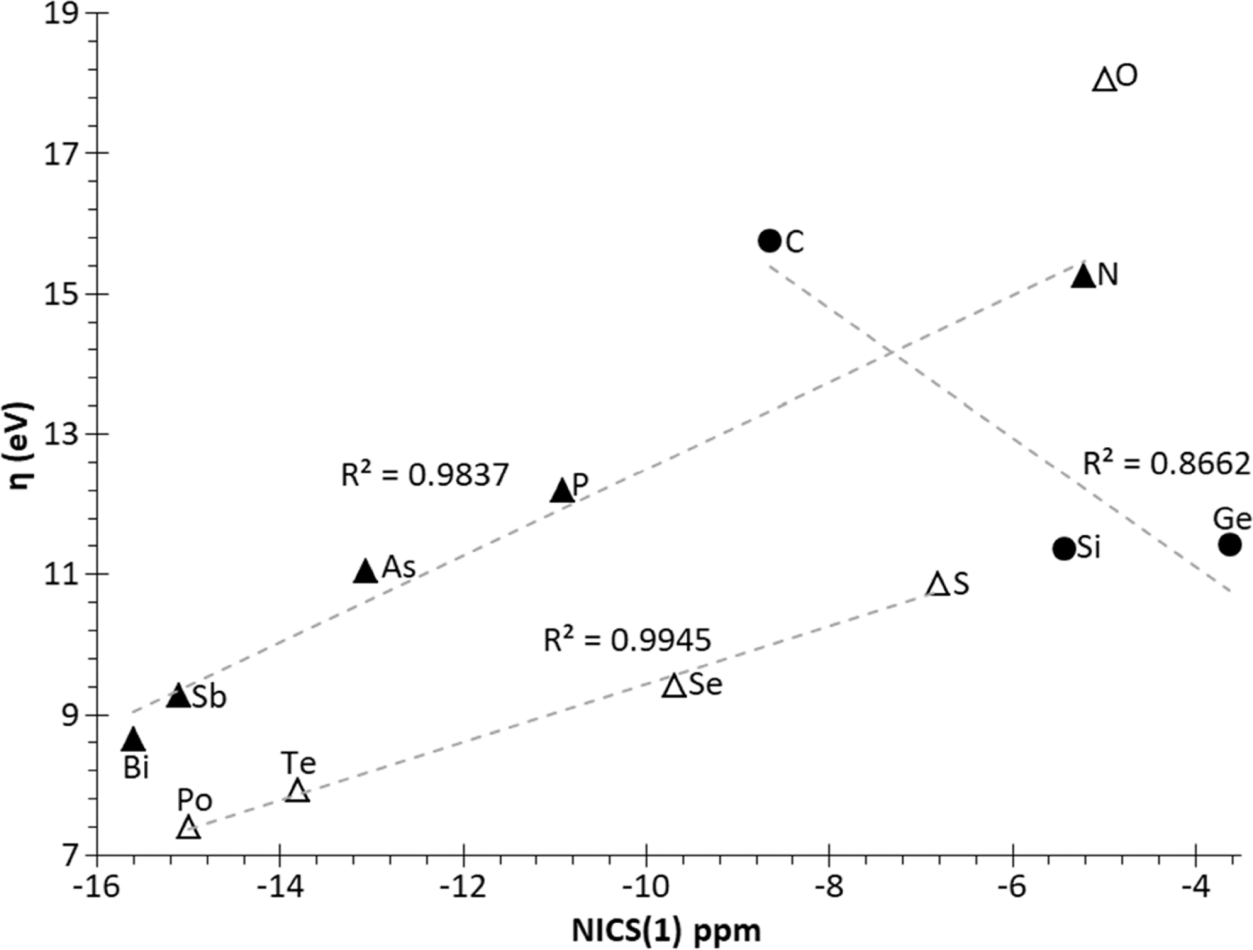
Plot of η vs NICS(1) for **1**^**El**^. Oxygen is excluded from the linear correlation of group 16.

However, Fowler pointed out that the (large) HOMO–LUMO separation cannot be seen as an absolute criterion for the aromaticity or kinetic stability in the case of polycyclic aromatic hydrocarbons, as lower gaps facilitate electronic transitions related to delocalization.^61^ In fact, there exists a clear linear correlation between more negative NICS(1) values (aromaticity) with lower H–L gap values in **1**^**El**^ rings for groups 15 and 16 (Figure 6). The latter is most likely just reflecting a decrease in AO energy differences as the principal quantum number increases when moving down the group. On the other hand, the lack of LPs in group 14 **1**^**El**^ rings entails higher gap between σ(El–El) and σ*(El–El), which is rather large for the scarcely σ-aromatic cyclopropane (*vide supra*) and decreases for heavier congeners, paralleling a decrease in σ-aromaticity (Figure 6).

**Figure 7:**
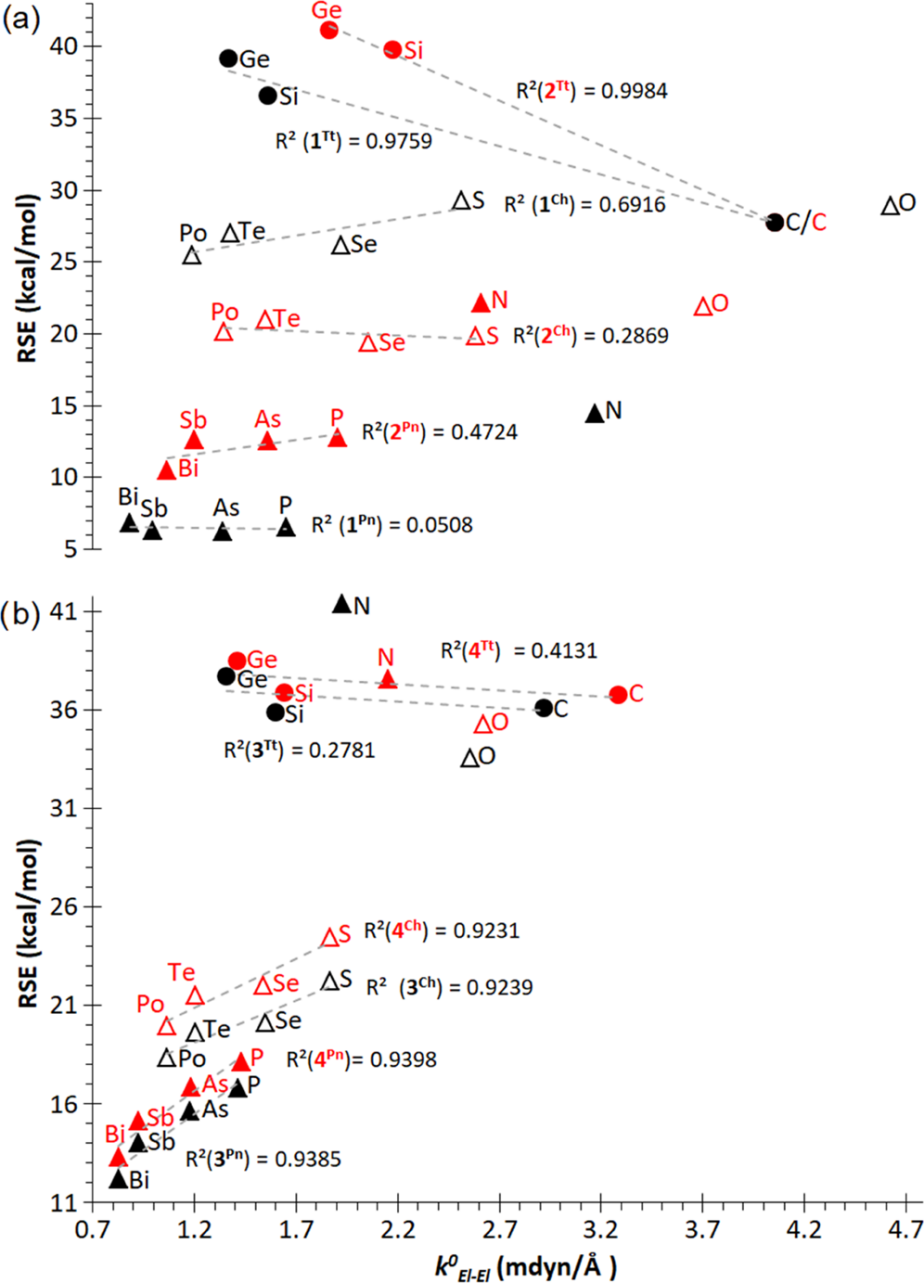
Plots of RSE against *k*_^0^El–El_ in El_2_Y rings: (a) **1**^**El**^ (Y: El, black), **2**^**El**^ (Y: C, red), (b) **3**^**El**^ (Y: Si, black), and **4**^**El**^ (Y: Ge, red). Correlation lines for the different ring systems and groups (Tt: tetrels; Pn: pnictogens; Ch: chalcogens) excluding O and N.

The increasing p-character in endocyclic bonds on descending the groups is expected to cause a decrease in bond stiffness. To evaluate this effect, relaxed force constants *k*^0^ were calculated for every endocyclic bond. The Hessian matrix in nonredundant internal coordinates is transformed into its inverse (or Moore–Penrose pseudo-inverse) providing the constant compliance matrix *C*_*ij*_ from which the relaxed force constants are obtained as reciprocals of the diagonal elements, *k*_*^0^ii*_ = 1/*C*_*ii*_. These are generally used as numerically stable and fully transferable parameters.^62^ The four ring types **1**–**4** show a remarkable decrease in the El–El bond strength, *k*_^0^El–El_, for El heteroatoms from the third row onward, in line with an increase in the p-character, especially for pnictogen and chalcogen El elements (Figure 7) where LPs strain relaxation^29,51^ is possible. As already observed for the p-character, for **1**^**El**^ and **2**^**El**^ rings, the expected decrease in *k*_^0^El–El_ on moving down the group does not lead to a significant relaxation of the ring strain, except for **2**^**Pn**^ to some extent (Pn = pnictogen atoms). By contrast, in **3**^**El**^ and **4**^**El**^ rings, the RSE decreases significantly with the increase of bond flexibility with significant linear correlation (*R*^2^ = 0.476 and 0.529 for **3**^**El**^ and **4**^**El**^, respectively).

**Figure 8:**
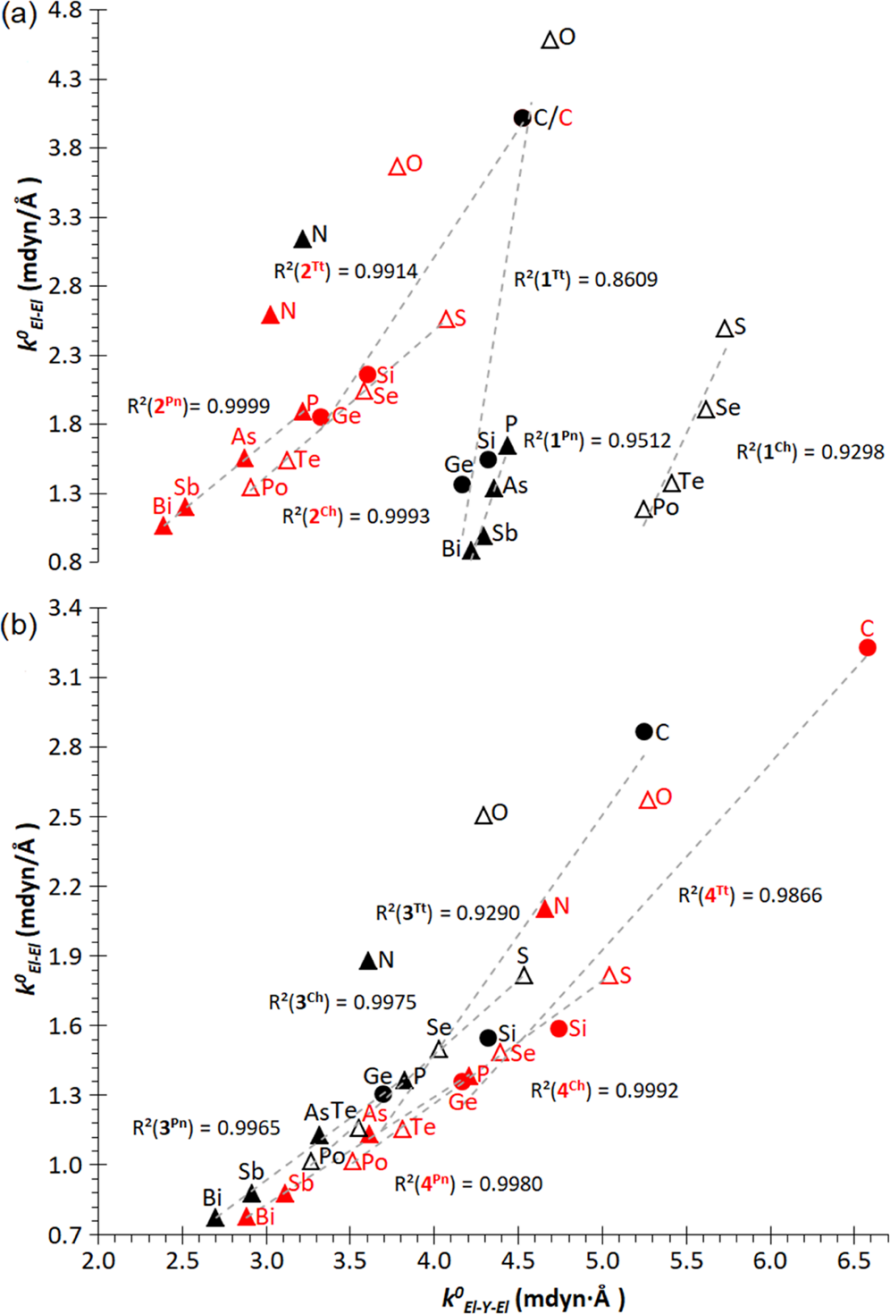
Plots of *k*_^0^El–El_ against *k*_^0^El–Y–El_ in El_2_Y rings: (a) **1**^**El**^ (Y: El, black), **2**^**El**^ (Y: C, red), (b) **3**^**El**^ (Y: Si, black), and **4**^**El**^ (Y: Ge, red). Correlation lines for the different ring systems and groups (Tt: tetrels; Pn: pnictogens; Ch: chalcogens) excluding O and N.

The force constant for the El–El bond, *k*_^0^El–El_, is expected to be coupled to some extent to that for the El–Y–El (Y: El, C, Si or Ge) bond angle, *k*_^0^El–Y–El_, as indeed observed by the systematic linear increase in *k*_^0^El–El_ when increasing *k*_^0^El–Y–El_ for all tetrel-containing rings **2**^**El**^, **3**^**El**^, and **4**^**El**^ (Figure 8). However, in homoatomic **1**^**El**^ rings, only some correlation is observed by groups, after excluding the lightest elements.

A decrease in *k*_^0^El–El–El_ (increase of bond angle elastic behavior) on descending the groups leads to a strain relaxation in all four types of rings, excluding the lightest (second row) and group 14 elements (Figure S6). In the case of homoatomic **1**^**El**^ rings, the bond angle flexibility and aromatic stabilization are counteracting effects that cause the RSE to remain largely unchanged when moving down the groups 15 and 16, except for the lightest **1**^**N**^ and **1**^**O**^ rings, the latter displaying low aromaticity and (comparatively) high bond angle flexibility. Simple acyclic model molecules HEl–El–ElH lacking competing aromaticity effects clearly show higher bond flexibility than the corresponding cyclic analogues El_3_, as expected, as well as (nonlinear) increasing angle flexibility on descending the groups 14–16 (Figure S7).

### Additive Estimation of RSEs

Although high-accuracy RSEs have been reported in the preceding section for a wide set of 3MRs, it would be desirable to have a quick method at hand for obtaining a rough estimation of RSEs for other unreported ring systems. Such methodology should consist of a summatory of contributions arising from the different constituent elements being either ring atoms and/or bonds. Therefore, as first approach, the RSE could be additively estimated (RSE^ad^) using only atomic addends *A*_*i*_ extended to the three constituent atoms *i* (eq 1) at any given ring.
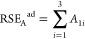
1Out of the 58 RSE values herein calculated for rings **1**–**4** (Table 1), only 49 were used, excluding those with Sn heteroatoms (**1**–**4**^**Sn**^) because they introduce uncompensated effects (*vide supra*) in the RSE calculation, as well as the only herein described Pb- and Tl-containing ring (**1**^**Pb**^ and **1**^**Tl**^), which are not suitable for later stages. In addition, 12 previously reported RSE values^29^ were included at (approximately) the same level^63^ for monohetero-monocycles **5**^**El**^. To calculate the contributions to the ring strain of the triels group, **5**^**Al**^ and **5**^**Ga**^ were also included, in addition to six new molecules Tt_2_Tr and CSiTr (where Tt: Si, Ge and Tr: Al, Ga)^64^ (*vide infra*). In this way, an oversized system of 67 equations (eq 1) with 15 A_1_^El^ unknowns can be written.

Upon numerical resolution of this system, a set of parameters *A*_1_^El^ (Table 2) with a high RMSE of 4.813 kcal/mol was obtained. This is in line with the highly dispersed plot of estimated RSE_A_^ad^ against the accurately (RC4-based) computed RSE (Table 1), with moderate *R*^2^ = 0.8175 (Figure S11a). A testbed of 13 new saturated 3MRs (Si_2_P, Si_2_S, Ge_2_N, CSiN, CSiP, CSiO, CSiS, CGeO, CGeAl, CGeGa, SiGeAl, SiGeGa, and SiGeO), not included in the set of rings belonging to the **1**–**5** categories (Figure 1), was used only for checking the performance of the additive method, by comparing the accurate RSE values resulting from the evaluation of RC4-type homodesmotic reactions (Scheme 1) with the RSE_A_^ad^ estimation (Table 3). The estimates arising from this approximation are not very close to the reference values but, despite being a crude method, it can give an idea of the RSE with a maximum unsigned absolute error (unsigned difference) of 10.63 kcal/mol. For instance, for the additive estimation of the RSE for alumasilagermirane (AlSiGe), the atomic-strain contributions *A*_1_ (in kcal/mol) for Al (19.87), Si (12.21), and Ge (12.85) are summed up, leading to RSE_A_^ad^ = 44.93 kcal/mol, which overestimates in 5.22 kcal/mol the reference most accurately computed RSE value of 39.71 kcal/mol obtained by evaluation of homodesmotic reactions.

**Table 2:** Calculated Atoms *A*_1_^El^ and Bond-Strain Contributions *B*_2_^El–El^, *B*_2_^C–El^, *B*_2_^Si–El^, and *B*_2_^Ge–El^ (kcal/mol) to RSE^ad^

El	*A*_1_^El^	*B*_2_^El–El^	*B*_2_^C–El^	*B*_2_^Si–El^	*B*_2_^Ge–El^
Al	19.87		17.76	13.07	13.57
Ga	21.37		17.61	14.34	14.87
C	8.31	8.48	8.48	13.71	13.93
Si	12.21	12.07	13.71	12.07	12.52
Ge	12.85	12.89	13.93	12.52	12.89
N	8.54	4.55	8.99	18.52	16.60
P	2.29	1.93	5.55	7.54	8.21
As	1.90	1.93	4.97	6.95	7.55
Sb	1.52	2.05	4.45	6.08	6.66
Bi	0.91	2.32	2.94	5.05	5.59
O	9.54	9.15	7.58	12.32	13.19
S	6.99	9.61	4.79	6.42	7.53
Se	6.03	8.71	4.45	5.81	6.75
Te	6.09	9.18	4.15	5.34	6.27
Po	5.44	8.77	3.46	4.89	5.69

**Table 3:** Calculated (DLPNO-CCSDT/def2TZVPPecp) Accurate (RC4) and Additively Estimated (ad) RSEs (kcal/mol) for a Testbed of 3MRs^a^

	RSE_RC4_	RSE_A_^ad^	RSE_B_^ad^
Si_2_P	27.70	26.72 (−0.98)	27.16 (−0.54)
Si_2_S	24.68	31.42 (6.74)	24.90 (0.22)
Ge_2_N	41.76	34.23 (−7.53)	46.10 (4.34)
CSiN	39.69	29.07 (−10.63)	41.22 (1.53)
CSiP	26.79	22.82 (−3.97)	26.80 (0.01)
CSiO	38.70	30.07 (−8.63)	33.60 (−5.10)
CSiS	24.40	27.52 (3.11)	24.91 (0.51)
CGeO	37.81	30.70 (−7.11)	34.69 (−3.12)
AlCGe	43.45^b^	41.03 (−2.42)	45.25 (1.80)
GaCGe	45.90	42.53 (−3.38)	46.41 (0.50)
AlSiGe	39.71	44.93 (5.22)	39.16 (−0.56)
GaSiGe	42.17	46.43 (4.26)	41.74 (−0.43)
SiGeO	42.58	34.60 (−7.98)	38.02 (−4.56)

a Signed absolute errors are in parenthesis.

b Calculated only with the contribution of the C–Al and C–Ge bond-cleavage homodesmotic reactions due to convergence problems in the third ring-opening reaction.

**Figure 9:**
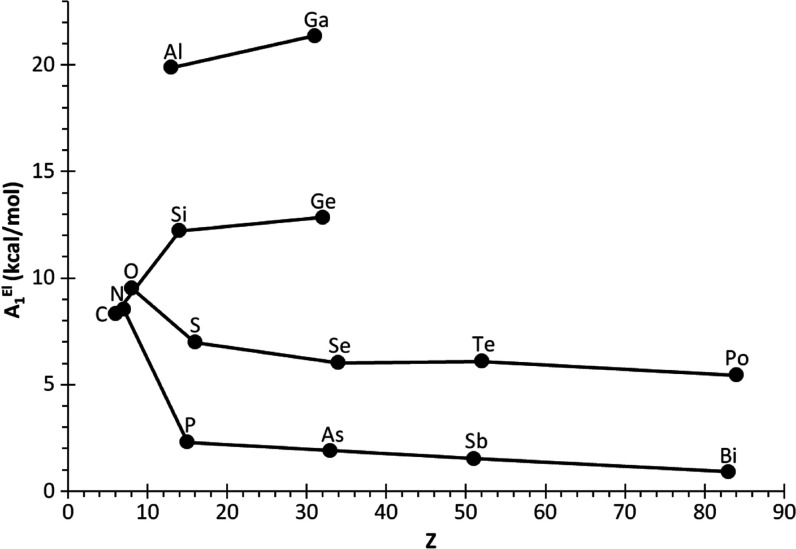
Variation of the calculated atom-strain contributions **A**_**1**_^**El**^ (kcal/mol) to RSE_1_^ad^ with the atomic number.

The variation of the obtained atom-strain contributions *A*_1_^El^ (Figure 9) is in agreement with the general tendencies reported^29^ for heterocycles containing one heteroatom, **5**^**El**^. Thus, second-row elements (C, N, and O) have similar values. On descending the groups, the atom-strain contributions decrease for elements having LPs (groups 15 and 16), especially for pnictogens, whereas they increase for those lacking LPs (groups 13 and 14), the highest values being observed for triels that bear an empty p orbital (Figure 9). This effect is most likely related to the already reported LP strain releasing effect,^29,51^ in turn affecting the p-character of the endocyclic bonds (see Figure 4).

**Figure 10:**
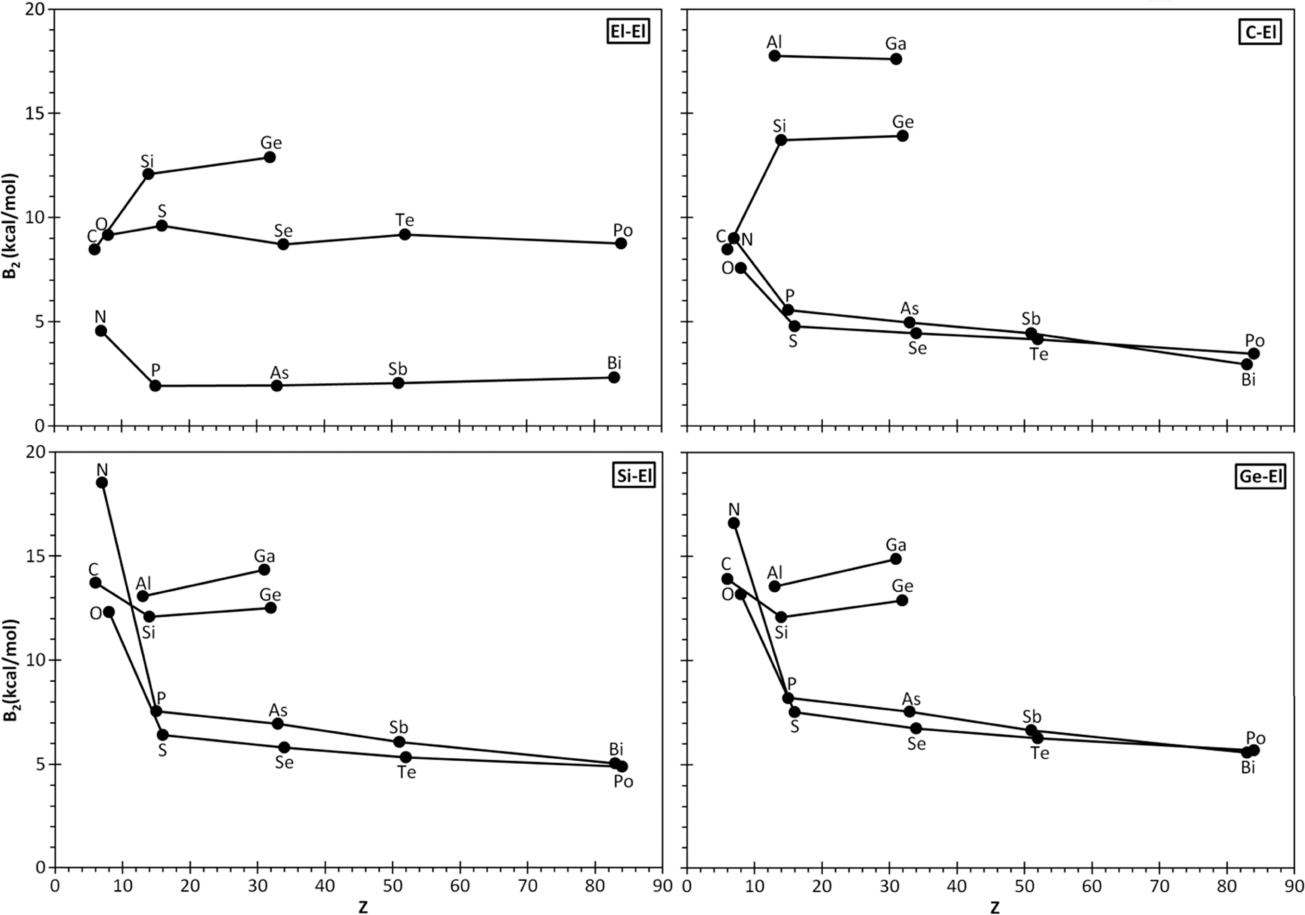
Variation of the calculated bond-strain contributions *B*_2_ (kcal/mol) to RSE_2_^ad^ with the atomic number.

A refinement of the methodology consists of using bond-related addends *B*_2*i*_ in the additive estimation RSE_B_^ad^ (eq 2), instead of atom-based parameters. Resolving the corresponding overdimensioned 67 equation system with 52 *B*_2_^El–El′^ variables (Table 2), the remarkably lower RMSE of 1.168 kcal/mol reflects the better estimation resulting from this approximation (Table 3). For the abovementioned example of the AlSiGe ring, the bond-based additive estimation would result from the summation of the strain contributions *B*_2_ (in kcal/mol) for Si–Al (13.07), Si–Ge (12.52), and Ge–Al (13.57), resulting in RSE_B_^ad^ = 39.16 kcal/mol, which only underestimates in 0.56 kcal/mol the reference value.
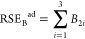
2Analysis of the *B*_2_^El–El′^ bond-strain parameters reveals some interesting features (Figure 10), mostly in line with the abovementioned atom-strain contributions. As expected, bonds involving group 13–14 elements contribute the highest bond energy strain for all four types of rings **1**–**4**^**El**^. Strain contributions are lower for bonds involving group 16 than group 15 elements, except for homoatomic El–El bonds (Figure 10). Remarkably high strain contributions are obtained for bonds of N with the heaviest tetrels (Si and Ge).

**Figure 11:**
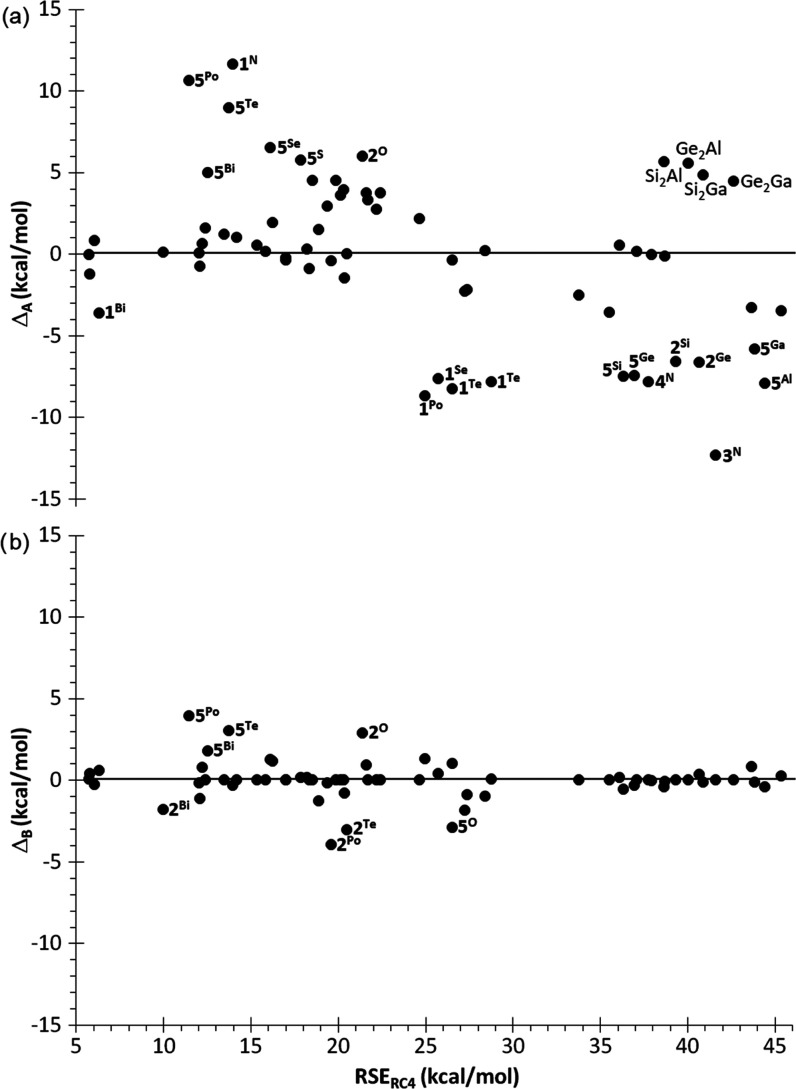
Plots of deviations Δ of additively estimated RSE based on (a) atom-strain and (b) bond-strain contributions vs the reference RSE_RC4_.

Worth noting is that on plotting RSE_B_^ad^ versus RSE (Figure S11b), the scarcely dispersed linear correlation (*R*^2^ = 0.9893) becomes obvious, with three **5**^**El**^ rings (El = Po, Te, Bi) showing the highest positive deviations (Δ = RSE_B_^ad^ – RSE of 3.94, 3.02, and 1.81 kcal/mol, respectively), while the corresponding **2**^**El**^ rings feature the largest negative deviations (−3.94, −3.02, and −1.81 kcal/mol, respectively). This fact strongly suggests an overestimation of the three *B*_2_^C–El^ and underestimation of the three *B*_2_^El–El^ parameters for the three abovementioned El elements, assuming that *B*_2_^C–C^ is properly estimated (as indeed evidenced by the correct additive estimation of all other **5**^**El**^ rings). The opposite effect is observed for **5**^**O**^ (Δ = −2.91 kcal/mol) and **2**^**O**^ (Δ = 2.91 kcal/mol), hinting at an underestimated *B*_2_^C–O^ and overestimated *B*_2_^O–O^ strain parameters (Figure 11).

One possibility for further refinement of the additive methodology would consist of using both atom- and bond-based addends in the third level of ring strain estimation, RSE_A&B_^ad^ (eq 3).
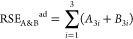
3With the 49 equations resulting from molecules **1**–**4** together with the 12 additional equations of type **5**, an underdimensioned system of 67 (atom- and bond-based) unknowns with only 61 equations is obtained. Therefore, to balance the system, the abovementioned six rings (equations) (Tt_2_Tr and CSiTr, where Tt: Si, Ge and Tr: Al, Ga) had to be included, provided that they do not introduce new unknowns. In case of boron, no cyclic minima were found for Tt_2_B, whereas for Tt_2_In and Tt_2_Tl, all cyclic minima displayed unconventional geometries (*vide supra*) not allowing accurate calculation of RSE. Worth noting is that in the case of Si_2_Tr (Tr: Al, Ga), there exists a conformational preference (without significant variation in hybridization) for the H_2_Si–SiH_2_ moiety featuring two axial H atoms in relative antiperiplanar orientation and two equatorial H in a synclinal conformation.^65^

The resulting equidimensional system of 67 equations with 67 unknowns has not a single but an infinite number of mathematical solutions with the same RMSE (1.168 kcal/mol) which, in turn, does not essentially outperform that obtained with the only-bonds method (no variation up to the 13th decimal figure).

It is possible to make a directed search to ascertain what is the most meaningful solution through a stepwise procedure. This requires a thermochemical evaluation of appropriate reactions for the estimation of electronic strain energy contribution of every X–Y endocyclic bond and using them as boundary conditions (see the SI). However, it resulted in not worth the great effort required for this much more elaborated methodology, as the ensuing set of *A*_4_^El^ and bond *B*_4_^El^ strain contributions (Table S5) do not represent any improvement regarding mathematical accuracy compared to the rather simple only-bonds additive estimation method for RSEs. Furthermore, it seems to overestimate the bond-strain contributions *B*_4_^El^ (Figure S8) but at the price of compensating with increasingly negative atom-strain contributions *A*_4_^El^ (Figure S9) for atoms typically involved in more strained rings.

## Experimental Section

Density functional theory (DFT) calculations were performed with the ORCA program.^66^ All geometry optimizations were run in redundant internal coordinates in the gas phase, with tight convergence criteria, and using the B3LYP^67^ functional together with Ahlrichs segmented def2-TZVP basis set^68^ and the latest Grimme’s semiempirical atom-pairwise London dispersion correction (DFT-D4).^69^ From these geometries, all electronic data were obtained through single-point calculations (SP) using the same quality basis set but including additional polarization, def2-TZVPP.^70^ Energy values were corrected for the zero-point vibrational term at the optimization level and obtained by the newly developed DLPNO method^71^ for the “coupled-cluster” level with single, double, and triple perturbatively introduced excitations (CCSD(T)).^72^ Analysis of the hybridization in the AO used for the endocyclic bonds was performed with the NBO method.^73^ Properties derived from the topological analysis of the electronic density were obtained with the Multiwfn program,^74^ and MO was drawn with Visual Molecular Dynamics (VMD).^75^

## Conclusions

Accurate high-level (DLPNO-CCSD(T)/def2TZVPP//B3LYP-D4/def2TZVP) values were provided for the ring strain energy (RSE) in three-membered symmetric inorganic rings El_3_ and organic dihetero-monocycles El_2_C and their silicon El_2_Si and germanium El_2_Ge analogues for group 14–16 heteroatoms El. The absence of undistorted cyclic minima prevented the design of suitable homodesmotic reactions for triel-containing **1**–**4**^El^ rings. For the **1**^**Tl**^, **1**^**Sn**^, and **1**^**Pb**^ rings containing endocyclic El–El donor–acceptor bonds, the general homodesmotic reactions used for other rings should be taken with caution due to uncompensated effects. Only in case of **1**^**Pb**^, suitable alternative homodesmotic reactions with compensation of Pb–Pb donor–acceptor bonds with twisted substituents provided a good estimation of its RSE. With some exceptions in the **1**–**4**^El^ rings, pnictogen and chalcogen-containing derivatives exhibit a clear relaxation of the ring strain that increases on descending the groups, due to the increase of the s-character of the LP-containing orbital which promotes an increase of p-character of the AO used by El for the endocyclic bonds, in turn decreasing the bond stiffness. The simultaneous counteracting increase in aromaticity for **1**^**El**^ rings on moving down in groups 15–16 let the RSE almost unaffected. The latter has been analyzed based on the counter-intuitive correlation between increasing aromaticity and decreasing H–L gap, most likely arising from a decrease in AO energy differences as the principal quantum number increases down the group.

Finally, a new fast method of estimating the ring strain based on the additivity of atom- and/or endocyclic bond-strain contributions is proposed, thus providing an attractive and efficient alternative approximation to the RSE. The method of choice is based on a summatory of only-bonds addends and can be currently applied to any three-membered saturated ring containing only El–El and El–Tt (Tt = C, Si, Ge) bonds.

## Supporting Information Available

Plots of the relaxed force constants and RSE versus other parameters, additional tables with electronic, structural, and bond strength parameters, and Cartesian coordinates and energies for all calculated species (PDF)
