# Supplementary material for: Ring Strain Energies of Three-Membered Homoatomic Inorganic Rings El3 and Diheterotetreliranes El2Tt (Tt = C, Si, Ge): Accurate versus Additive Approaches
Source: Inorg Chem. 2022 Aug 24;61(35):13846–57. doi: 10.1021/acs.inorgchem.2c01777 (PMC9449980; doi:10.1021/acs.inorgchem.2c01777)
Supplement: Supplementary file 1 — ic2c01777_si_001.pdf [file ic2c01777_si_001.pdf]

Supporting Information

Ring Strain Energies of Three-Membered Homoatomic Inorganic Rings El_3 and Diheterotetreliranes El_2Tt (Tt = C, Si, Ge). Accurate *versus* Additive Approaches.

Alicia Rey Planells[a] and Arturo Espinosa Ferao*[a]

[a] Depto. Química Orgánica, Facultad de Química, Campus de Espinardo, Universidad de Murcia, 30100 Murcia (Spain), E-mail: artuesp@um.es.

Table of contents

Figure S1. El_3H_3 isomers (El : In, Tl) and their respective relative ΔE_{ZPE} (kcal/mol) S1

Figure S2. Representation of the $\nabla^2\rho$ for **1**^{Pb}: a) contour map; b) variation along the Pb−Pb bond path. S1

Figure S3. Optimized structure and computed BCP, RCP and bond paths for **4**^{Sn}. S2

Figure S4. Plot of RSE vs s-character (%) of AO used by El for its LP in **1**^{El}. S2

Table S1. s-character (%) of AO used by the heteroatom El for its LP in **1**^{El}. S2

Figure S5. Computed Kohn−Sham isosurfaces for HOMO−5 of a) **1**^{C} and b) **1**^{Si}. S2

Figure S6. Plots of RSE against $k^0_{El\text{-}Y\text{-}El}$ in El_2Y rings. S3

Figure S7. Plot of $k^0_{El\text{-}El\text{-}El}$ in **1**^{El} and acyclic HEl-El-ElH species against NICS(1) of **1**^{El}. S4

Table S2. Calculated C-C, C-El bond distances and their respectives WBI and (G/ρ) at BCP for compounds **1-4**^{El}. S5

Table S3. Computed, ΔE_{ZPE}, for trichalcogene E_3 isomers. S7

Final refinement of the atoms and bonds additive estimation methodology for RSEs S8

Scheme S1. [2+2] Cycloreversion reactions used for the estimation of BDE_{ring}. S8

Table S4. Calculated theoretical bond-strain contributions B_0 (kcal/mol). S8

Table S5. Calculated A_4^{El}, $B_4^{El\text{-}El}$, $B_4^{C\text{-}El}$, $B_4^{Si\text{-}El}$ and $B_4^{Ge\text{-}El}$ (kcal/mol) to RSE^{ad}. S9

Figure S8. Variation of the calculated $B_4^{El\text{-}El}$, $B_4^{C\text{-}El}$, $B_4^{Si\text{-}El}$ and $B_4^{Ge\text{-}El}$ with Z. S9

Figure S9. Variation of the calculated A_4^{El} (kcal/mol) with Z. S10

Figure S10. Optimized structures and computed BCP, RCP and bond paths for **2-4**^{Tl} S10

Figure S11. Plot of a) RSE_A^{ad} and b) RSE_B^{ad} vs RSE_{RC4}. S11

Calculated structures. Cartesian coordinates and energies for all computed minima. S12-88

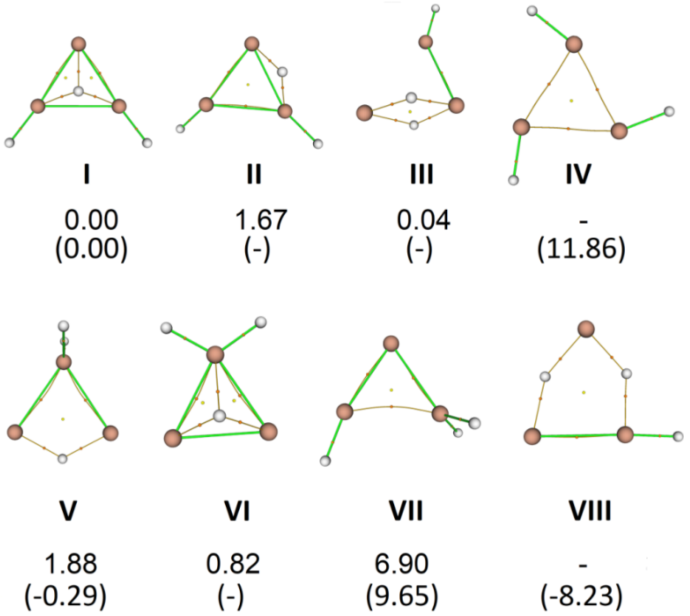

Figure S1. Optimized structures of heaviest El_3H_3 isomers (El : In, Tl) and their respective relative ZPE corrected energy values (kcal/mol) calculated at DLPNO-CCSD(T)/def2-TZVPP(ecp) level for In (and Tl in parenthesis). Ring critical points, bond critical points and bond paths are indicated. Geometries correspond to In when both triels (**I**, **V**, **VII**) or only In (**II**, **III**, **VI**) are available and to Tl in the other two (**IV** and **VIII**).

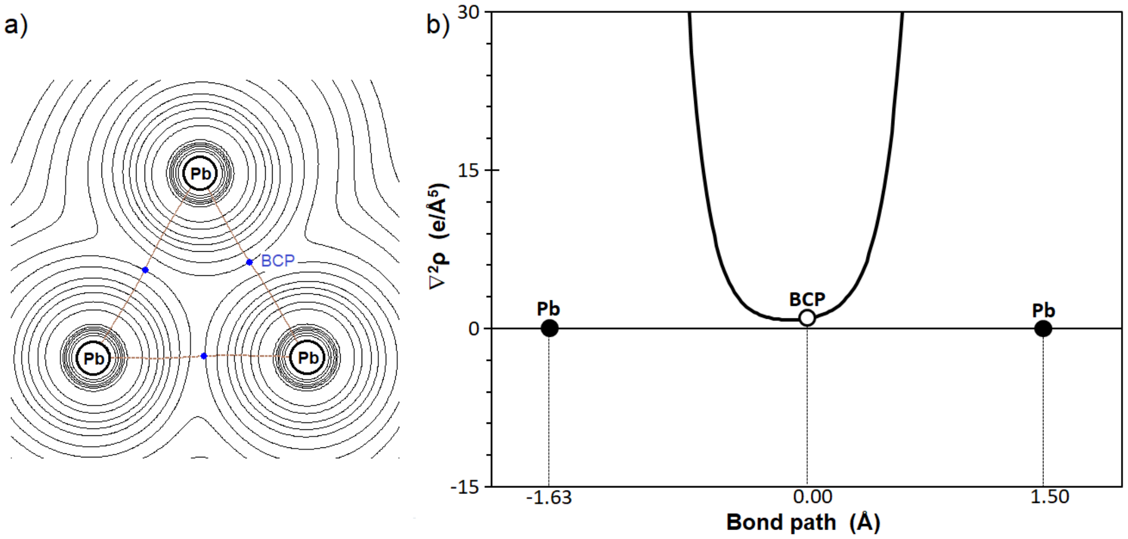

Figure S2. Computed (B3LYP-D4/def2-TZVPPecp) representation of the Laplacian of electron density $\nabla^2\rho$ for $\mathbf{1}^{\mathbf{Pb}}$: a) contour map at the ring plane with BCPs (blue) and bond paths indicated; b) variation along the Pb−Pb bond path.

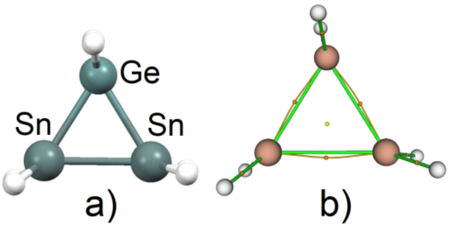

Figure S3. Optimized structure and computed (B3LYP/def2-TZVPP) BCP (small orange spheres), RCP (small yellow sphere) and bond paths for **4**^{Sn}.

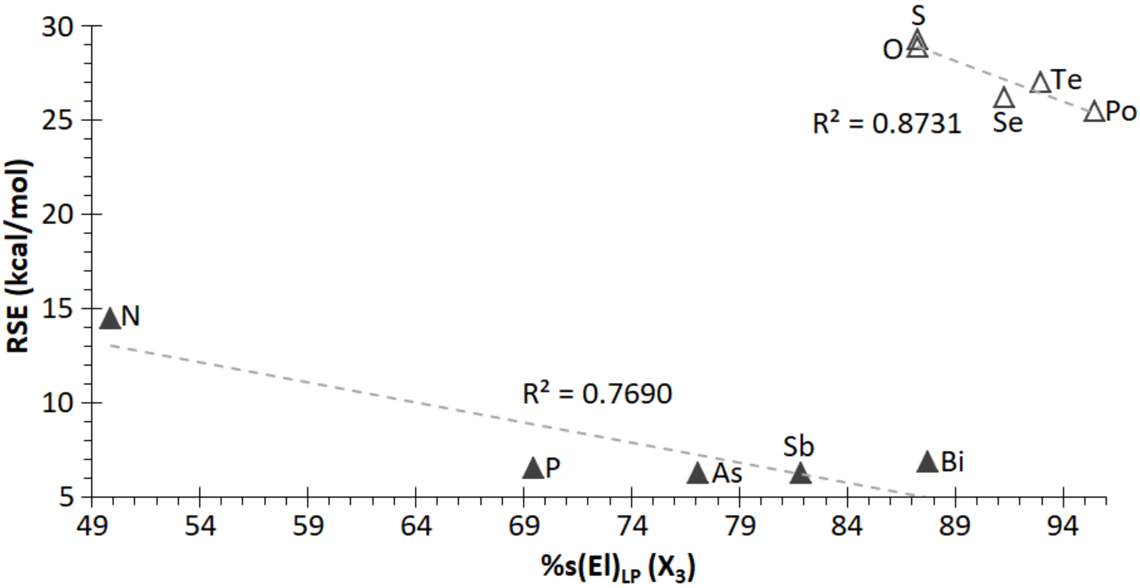

Figure S4. Plot of RSE vs s-character (%) of AO used by the heteroatom El for its LP in **1**^{El}.

Table S1. s-character (%) of AO used by the heteroatom El for its LP in **1**^{El}.

**1^{El} (X_3)**	**N_3**	**P_3**	**As_3**	**Sb_3**	**Bi_3**	**O_3**	**S_3**	**Se_3**	**Te_3**	**Po_3**
**%s(El)$_{LP}$**	49.88	69.46	77.1	81.88	87.75	87.27	87.24	91.26	92.94	95.45

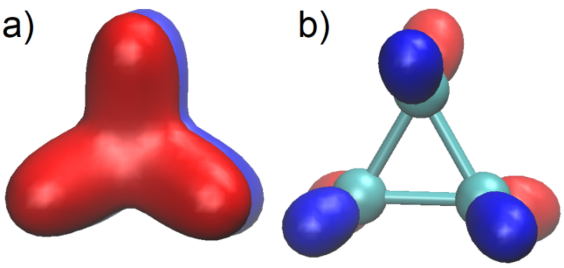

Figure S5. Computed (B3LYP-D3/def2-TZVP) Kohn−Sham isosurfaces (0.07 au) for HOMO−5 of a) **1**^{C} and b) **1**^{Si}.

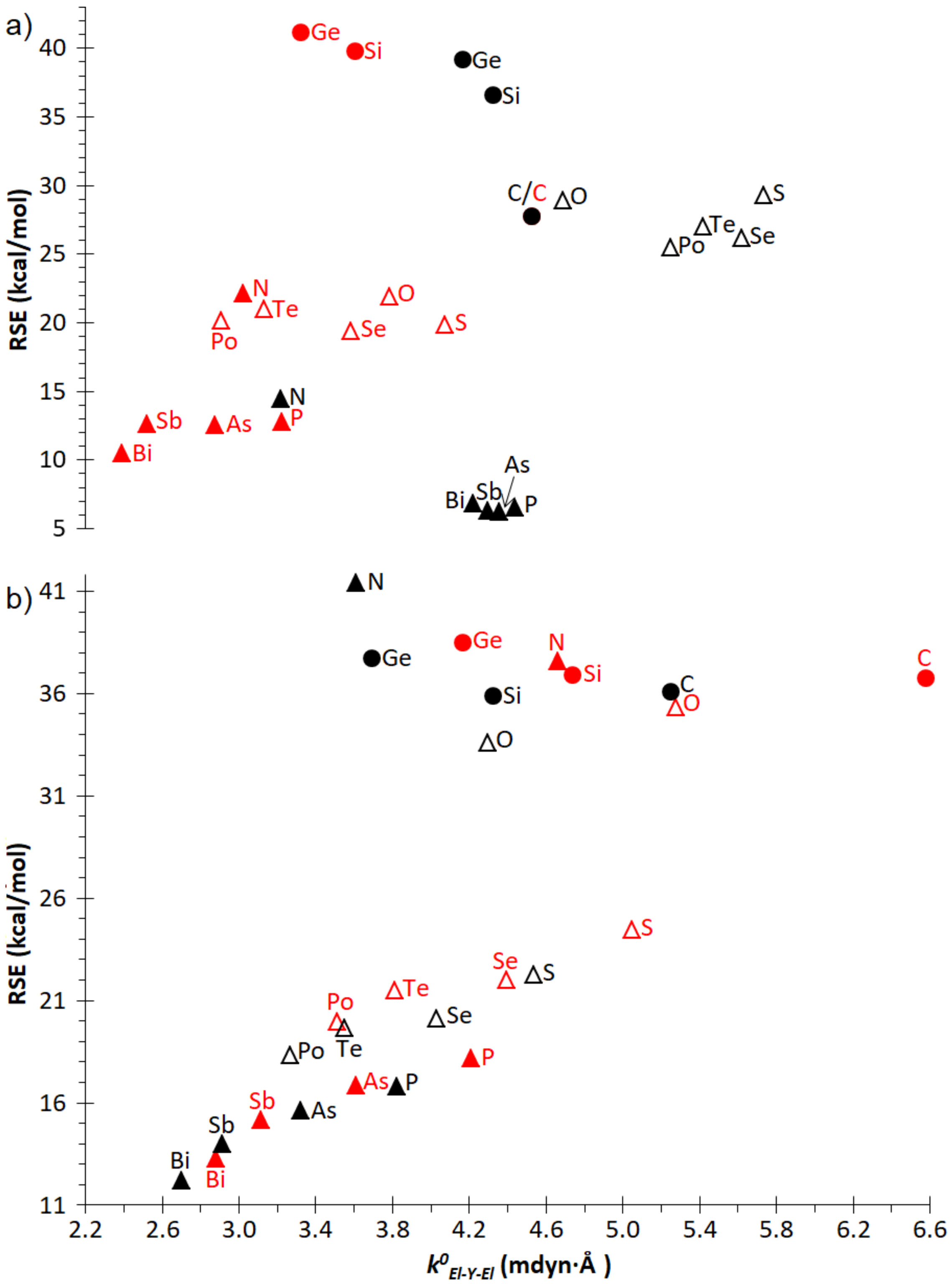

Figure S6. Plots of RSE against $k^0_{El\text{-}Y\text{-}El}$ in El_2Y rings: a) **1**^{El} (Y: El, black), **2**^{El} (Y: C, red); b) **3**^{El} (Y: Si, black), **4**^{El} (Y: Ge, red).

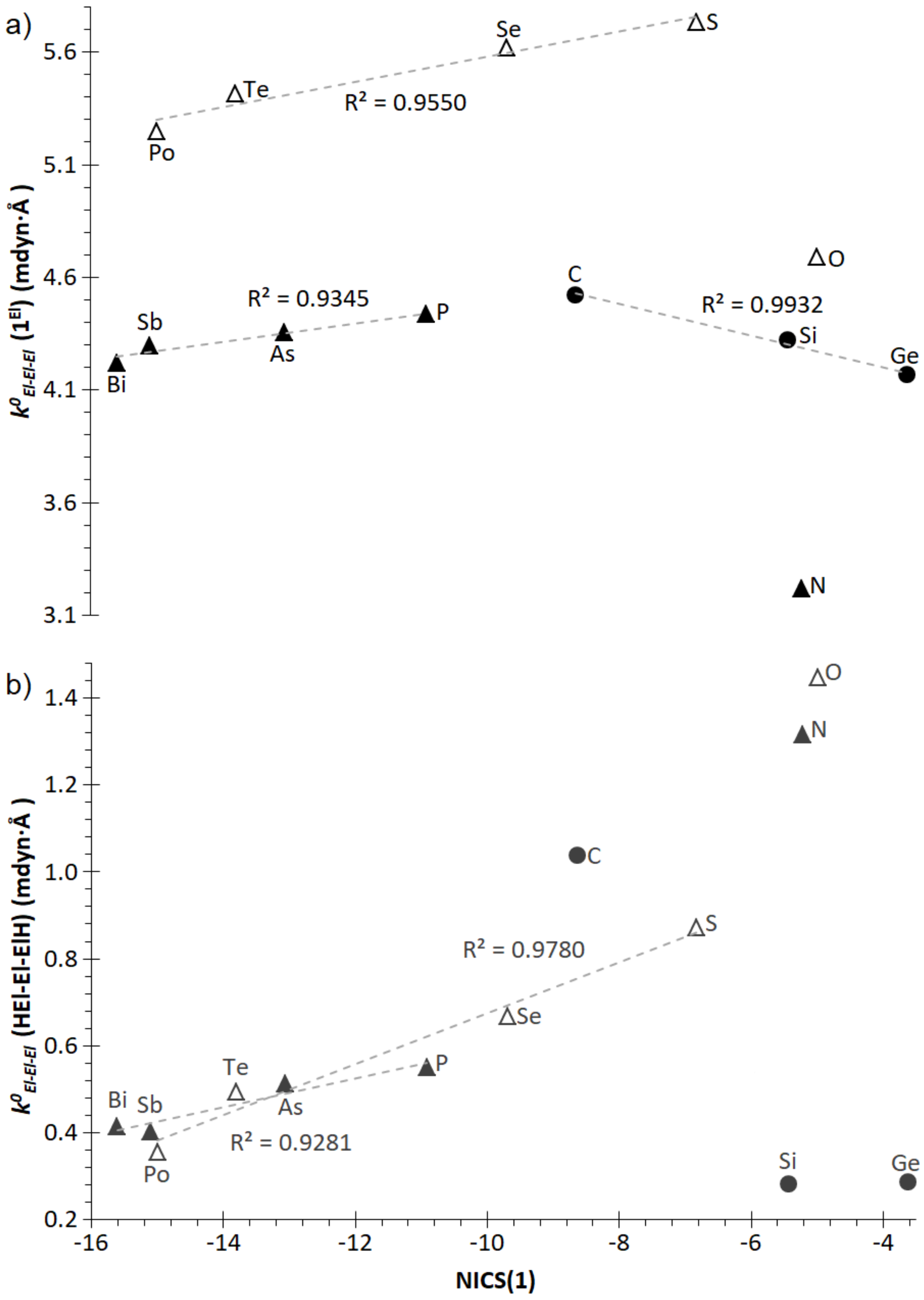

Figure S7. Plot of $k^0_{El\text{-}El\text{-}El}$ in $\mathbf{1}^{\mathbf{El}}$ (black) and acyclic HEl-El-ElH species (gray) against NICS(1) values of $\mathbf{1}^{\mathbf{El}}$. Excluding $\mathbf{1}^{\mathbf{N}}$ and $\mathbf{1}^{\mathbf{O}}$ and the corresponding linear structure from the linear correlation.

Table S2. Calculated C-C, C-El bond distances (Å) and their respectives WBI and Lagrangian kinetic energy density per electron (G/ρ) at BCP for compounds **1-4^{El}**.

**1^{El}**	d_{El-El} (Å)	WBI_{El-El}	G_{El-El} (au)	ρ_{El-El} (au)	$(G/\rho)_{El-El}$ (au)	G_{RCP} (au)	ρ_{RCP} (au)	$(G/\rho)_{RCP}$ (au)
C	1.504	1.001	0.091	0.241	0.377	0.147	0.200	0.738
Si	2.339	0.989	0.012	0.088	0.135	0.025	0.065	0.380
Ge	2.455	0.967	0.020	0.078	0.253	0.026	0.054	0.479
Sn	2.881	0.920	0.015	0.048	0.308	0.013	0.032	0.405
Pb	3.137	0.548	0.014	0.031	0.431	0.008	0.022	0.372
N[a)]	1.460	1.008	0.162	0.281	0.577	0.233	0.214	1.087
P[a)]	2.235	1.018	0.029	0.107	0.269	0.038	0.084	0.455
As[a)]	2.478	1.004	0.025	0.079	0.307	0.025	0.059	0.428
Sb[a)]	2.868	1.001	0.015	0.054	0.279	0.014	0.039	0.355
Bi[a)]	3.025	0.996	0.016	0.046	0.336	0.011	0.032	0.355
O	1.320	1.005	0.243	0.288	0.844	0.291	0.206	1.409
S	2.080	1.040	0.054	0.133	0.405	0.064	0.098	0.655
Se	2.340	1.021	0.036	0.092	0.393	0.036	0.065	0.560
Te	2.767	1.019	0.021	0.063	0.340	0.019	0.043	0.451
Po	2.940	1.009	0.020	0.052	0.379	0.015	0.035	0.424

**2^{El}**	d_{El-El} (Å)	WBI_{El-El}	G_{El-El} (au)	ρ_{El-El} (au)	$(G/\rho)_{El-El}$ (au)	d_{C-El} (Å)	WBI_{C-El}	G_{C-El} (au)	ρ_{C-El} (au)	$(G/\rho)_{C-El}$ (au)	G_{RCP} (au)	ρ_{RCP} (au)	$(G/\rho)_{RCP}$ (au)
C	1.504	1.001	0.091	0.241	0.377	1.504	1.001	0.091	0.241	0.377	0.147	0.200	0.738
Si	2.256	0.974				1.904	0.875	0.109	0.112	0.974	0.035	0.084	0.412
Ge	2.373	0.958	0.026	0.080	0.328	2.000	0.880	0.076	0.115	0.661	0.041	0.074	0.549
Sn	2.718	0.920	0.021	0.056	0.368	2.202	0.852	0.062	0.094	0.652	0.029	0.052	0.561
N	1.497	0.992	0.159	0.255	0.624	1.444	1.007	0.143	0.271	0.527	0.208	0.214	0.971
P	2.214	1.001	0.034	0.105	0.323	1.859	0.977	0.093	0.147	0.632	0.050	0.101	0.493
As	2.454	0.982	0.030	0.077	0.385	1.990	0.962	0.064	0.126	0.505	0.038	0.076	0.499
Sb	2.827	0.981	0.019	0.052	0.363	2.191	0.927	0.058	0.099	0.587	0.023	0.052	0.443
Bi	2.983	0.971	0.019	0.044	0.430	2.286	0.926	0.053	0.091	0.580	0.022	0.044	0.502
O	1.498	0.977	0.229	0.241	0.948	1.387	0.969	0.242	0.279	0.870	0.256	0.217	1.182
S	2.097	1.014	0.059	0.127	0.462	1.799	1.035	0.066	0.179	0.366	0.080	0.118	0.677
Se	2.375	0.992	0.041	0.088	0.465	1.953	1.022	0.061	0.143	0.428	0.052	0.085	0.615
Te	2.744	0.995	0.025	0.060	0.418	2.160	0.993	0.057	0.109	0.526	0.031	0.059	0.530
Po	2.915	0.980	0.023	0.050	0.464	2.260	0.985	0.053	0.097	0.547	0.027	0.049	0.556

3^{El}	$d_{El\text{-}El}$ (Å)	$WBI_{El\text{-}El}$	$G_{El\text{-}El}$ (au)	$\rho_{El\text{-}El}$ (au)	$(G/\rho)_{El\text{-}El}$ (au)	$d_{Si\text{-}El}$ (Å)	$WBI_{Si\text{-}El}$	$G_{Si\text{-}El}$ (au)	$\rho_{Si\text{-}El}$ (au)	$(G/\rho)_{Si\text{-}El}$ (au)	G_{RCP} (au)	ρ_{RCP} (au)	$(G/\rho)_{RCP}$ (au)
C	1.504	1.001	0.091	0.241	0.377	1.504	1.001	0.091	0.241	0.377	0.147	0.200	0.738
Si	2.339	0.989	0.012	0.088	0.135	2.339	0.989	0.012	0.088	0.135	0.025	0.065	0.380
Ge	2.457	0.970	0.021	0.077	0.266	2.394	0.977	0.014	0.083	0.174	0.026	0.058	0.447
Sn	2.807	0.934	0.016	0.054	0.293	2.603	0.956	0.016	0.068	0.233	0.019	0.042	0.455
N	1.571	1.015	0.124	0.220	0.562	1.729	0.782	0.198	0.129	1.540	0.170	0.113	1.509
P	2.298	1.000	0.026	0.097	0.269	2.234	1.004	0.051	0.095	0.536	0.033	0.078	0.422
As	2.544	0.975	0.022	0.071	0.313	2.352	1.008	0.022	0.088	0.246	0.026	0.063	0.416
Sb	2.924	0.971	0.014	0.049	0.287	2.570	1.009	0.016	0.073	0.218	0.017	0.045	0.381
Bi	3.083	0.962	0.014	0.041	0.340	2.659	1.003	0.017	0.067	0.256	0.016	0.038	0.407
O	1.581	0.998	0.167	0.201	0.828	1.647	0.671	0.260	0.138	1.880	0.201	0.116	1.726
S	2.187	1.013	0.047	0.113	0.416	2.107	0.971	0.091	0.101	0.904	0.056	0.086	0.651
Se	2.462	0.990	0.032	0.080	0.406	2.256	1.006	0.057	0.092	0.618	0.036	0.070	0.513
Te	2.837	0.987	0.020	0.055	0.359	2.480	1.040	0.019	0.081	0.233	0.023	0.051	0.458
Po	3.008	0.974	0.018	0.046	0.393	2.577	1.040	0.018	0.074	0.246	0.020	0.043	0.462

4^{El}	$d_{El\text{-}El}$ (Å)	$WBI_{El\text{-}El}$	$G_{El\text{-}El}$ (au)	$\rho_{El\text{-}El}$ (au)	$(G/\rho)_{El\text{-}El}$ (au)	$d_{Ge\text{-}El}$ (Å)	$WBI_{Ge\text{-}El}$	$G_{Ge\text{-}El}$ (au)	$\rho_{Ge\text{-}El}$ (au)	$(G/\rho)_{Ge\text{-}El}$ (au)	G_{RCP} (au)	ρ_{RCP} (au)	$(G/\rho)_{RCP}$ (au)
C	1.504	1.001	0.091	0.241	0.377	1.504	1.001	0.091	0.241	0.377	0.147	0.200	0.738
Si	2.338	0.987	0.011	0.089	0.124	2.395	0.980	0.015	0.082	0.186	0.026	0.062	0.414
Ge	2.455	0.967	0.020	0.078	0.253	2.455	0.967	0.020	0.078	0.253	0.026	0.054	0.479
Sn	2.813	0.911	0.015	0.054	0.287	2.659[a)]	0.932[a)]	0.018[a)]	0.064[a)]	0.287[a)]	0.018	0.039	0.470
N	1.539	1.033	0.123	0.239	0.515	1.866	0.805	0.147	0.129	1.143	0.126	0.111	1.132
P	2.297	1.010	0.024	0.098	0.249	2.314	0.983	0.033	0.092	0.362	0.033	0.072	0.462
As	2.545	0.985	0.021	0.072	0.292	2.425	0.988	0.025	0.083	0.306	0.025	0.058	0.437
Sb	2.927	0.979	0.013	0.049	0.269	2.629	0.995	0.019	0.068	0.280	0.016	0.042	0.393
Bi	3.087	0.968	0.013	0.041	0.322	2.713	0.988	0.020	0.063	0.317	0.015	0.036	0.412
O	1.559	1.006	0.164	0.211	0.777	1.787	0.685	0.206	0.139	1.485	0.152	0.113	1.338
S	2.184	1.024	0.044	0.114	0.389	2.207	0.926	0.064	0.101	0.628	0.049	0.080	0.617
Se	2.463	1.000	0.030	0.080	0.379	2.346	0.962	0.041	0.090	0.459	0.034	0.064	0.535
Te	2.838	1.010	0.019	0.055	0.335	2.556	0.996	0.024	0.076	0.321	0.022	0.047	0.468
Po	3.009	0.9815	0.017	0.046	0.370	2.647	1.012	0.023	0.069	0.333	0.019	0.040	0.465

[a)]average values

Table S3. Computed zero-point corrected energies, ΔE_{rel} (kcal/mol), for trichalcogene E_3 isomers, relative to the most stable isomer in each case.

	**1^{O}**	**O_3**C_{2v}	**O3**$D_{\infty h}$	**1^{S}**	**S_3**C_{2v}	**1^{Se}**	**Se_3**C_{2v}	**1^{Te}**	**Te_3**C_{2v}	**Te_3**$D_{\infty h}$	**1^{Po}**	**Po_3**C_{2v}	**Po_3**$D_{\infty h}$[a)]
B3LYP-D4	29.30	0.00	80.50	8.10	0.00	0.00	1.67	0.00	7.02	60.59	0.00	8.97	39.23
DLNPO-CCSD(T)(fc)/def2-TZVPP	25.72	0.00	85.55	4.18	0.00	0.00	5.33	0.00	10.46	63.68	0.00	13.41	42.54

[a)] One imaginary frequency $\nu = -110.24\ cm^{-1}$

Final refinement of the atoms and bonds additive estimation methodology for RSEs

To ascertain which of the inifinite mathematical solutions of the atoms- and bond-strains additive methodology for RSE is the most physico-chemically meaningful one, the following stepwise procedure was proposed. First, the individual electronic bond dissociation energy for every X-Y endocyclic bond (BDE_{ring}) was roughly estimated as half of the energy associated to the [2+2]-cycloreversion reaction affording the X: and Y=Y molecular fragments (Scheme S3), *i.e.* BDE_{ring} = ½(E_X + E_{YY} – E_{XY2}). Some inaccuracy is assumed as the π(Y=Y) bond energy and possible (anti)aromatic character of the ring remain uncompensated. The strain component for the X-Y endocyclic bond was then computed as the difference B_0 = BDE_{RC4} – BDE_{ring} (see the SI), where the minuend refers to the most favourable (homo- or heterolytic) X-Y bond dissociation of the acyclic homodesmotic (RC4-type) ring cleavage product (see Scheme 1).

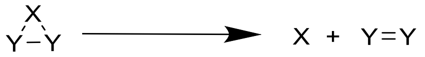

Scheme S1. [2+2] Cycloreversion reactions used for the estimation of BDE_{ring} of the X-Y bond in **1-5^{El}**.

In order to make an optimal refinement in which the atom- and bond-strain contributions have a reliable physical meaning, strict preliminary conditions were introduced to dampen possible abrupt changes in the numerical resolution of the equidimensional system. For this purpose, to the initial sixty-seven equations with sixty-seven (atoms- and bonds-based) unknowns (as in equation 3), other additional sixty-seven boundary equations were included using the B_0 values as fixed initial bond-strain contributions and the same set of atom-based unknowns. The overdimensioned system with a total of 134 equations and sixty-seven unknowns was numerically solved using zeroes and the B_0 contributions as starting values for the atoms and bonds-based unknowns, respectively. The obtained parameters were then used as starting values and further re-optimised using equation 4 and removing the boundary conditions.

$$RSE_{A\&B}{}^{ad} = \sum_{i=1}^{3}(A_{4i} + B_{4i}) \quad (4)$$

Despite the more elaborated last methodology, the resulting set of A_4^{El} and bond B_4^{El} strain contributions (Table S4) do not represent any improvement regarding accuracy (RMSE 1.168 kcal/mol) compared to the rather simple only-bonds method. Furthermore, inspection of the obtained parameters seems to overestimate the bond-strain contributions B_4^{El} (Figure S9), certainly keeping the expected physico-chemical sense, but at the price of compensating with increasingly negative atom-strain contributions A_4^{El} (Figure S9) for atoms typically involved in more strained rings. Therefore, it is not worth the great effort required for the atoms- and bonds-based methodology, as far as almost the same accuracy can be obtained using the only-bonds additive estimation method for RSEs.

Table S4. Calculated electronic bond-strain contributions B_0 (kcal/mol).

		**C-Al**	58.714	**Si-Al**	38.670	**Ge-Al**	39.089
		**C-Ga**	15.498	**Si-Ga**	40.778	**Ge-Ga**	42.544
**C-C**	43.720	**C-C**	43.720	**C-Si**	40.170	**C-Ge**	62.830
**Si-Si**	31.228	**C-Si**	40.170	**Si-Si**	31.228	**Si-Ge**	31.635
**Ge-Ge**	31.737	**C-Ge**	62.830	**Si-Ge**	31.635	**Ge-Ge**	31.737
**N-N**	2.756	**C-N**	28.883	**Si-N**	47.165	**Ge-N**	46.260
**P-P**	5.348	**C-P**	24.659	**Si-P**	29.175	**Ge-P**	30.005
**As-As**	2.563	**C-As**	43.060	**Si-As**	24.910	**Ge-As**	25.784
**Sb-Sb**	0.810	**C-Sb**	4.628	**Si-Sb**	21.037	**Ge-Sb**	22.151
**Bi-Bi**	1.781	**C-Bi**	77.571	**Si-Bi**	18.948	**Ge-Bi**	20.109
**O-O**	13.729	**C-O**	27.103	**Si-O**	18.237	**Ge-O**	17.186

**S-S**	-10.252	**C-S**	10.053	**Si-S**	13.990	**Ge-S**	15.063
**Se-Se**	-8.336	**C-Se**	29.990	**Si-Se**	9.990	**Ge-Se**	13.604
**Te-Te**	6.978	**C-Te**	-46.162	**Si-Te**	29.390	**Ge-Te**	-25.915
**Po-Po**	-50.015	**C-Po**	55.253	**Si-Po**	19.127	**Ge-Po**	-26.991

Table S5. Calculated atoms $\mathbf{A_4^{El}}$ and bonds strain contributions $\mathbf{B_4^{El\text{-}El}}$, $\mathbf{B_4^{C\text{-}El}}$, $\mathbf{B_4^{Si\text{-}El}}$ and $\mathbf{B_4^{Ge\text{-}El}}$ (kcal/mol) to RSE^{ad}.

El	$\mathbf{A_4^{El}}$	$\mathbf{B_4^{El\text{-}El}}$	$\mathbf{B_4^{C\text{-}El}}$	$\mathbf{B_4^{Si\text{-}El}}$	$\mathbf{B_4^{Ge\text{-}El}}$
Al	-29.150		45.758	38.935	36.150
Ga	-24.703		43.384	37.985	35.231
C	-26.851	35.328	35.328	38.422	35.357
Si	-22.580	34.654	38.422	34.654	31.815
Ge	-16.014	28.908	35.357	31.815	28.908
N	-4.975	9.528	24.906	32.296	27.098
P	-7.129	9.061	22.543	22.399	19.781
As	-7.845	9.778	22.313	22.161	19.478
Sb	1.100	0.950	17.326	16.820	14.111
Bi	-19.493	21.807	26.114	26.084	23.341
O	-4.992	14.141	23.502	26.101	23.688
S	18.617	-9.007	8.903	8.397	6.228
Se	11.431	-2.721	12.164	11.385	9.037
Te	24.616	-15.441	5.267	4.325	1.967
Po	27.703	-18.938	3.031	2.331	-0.157

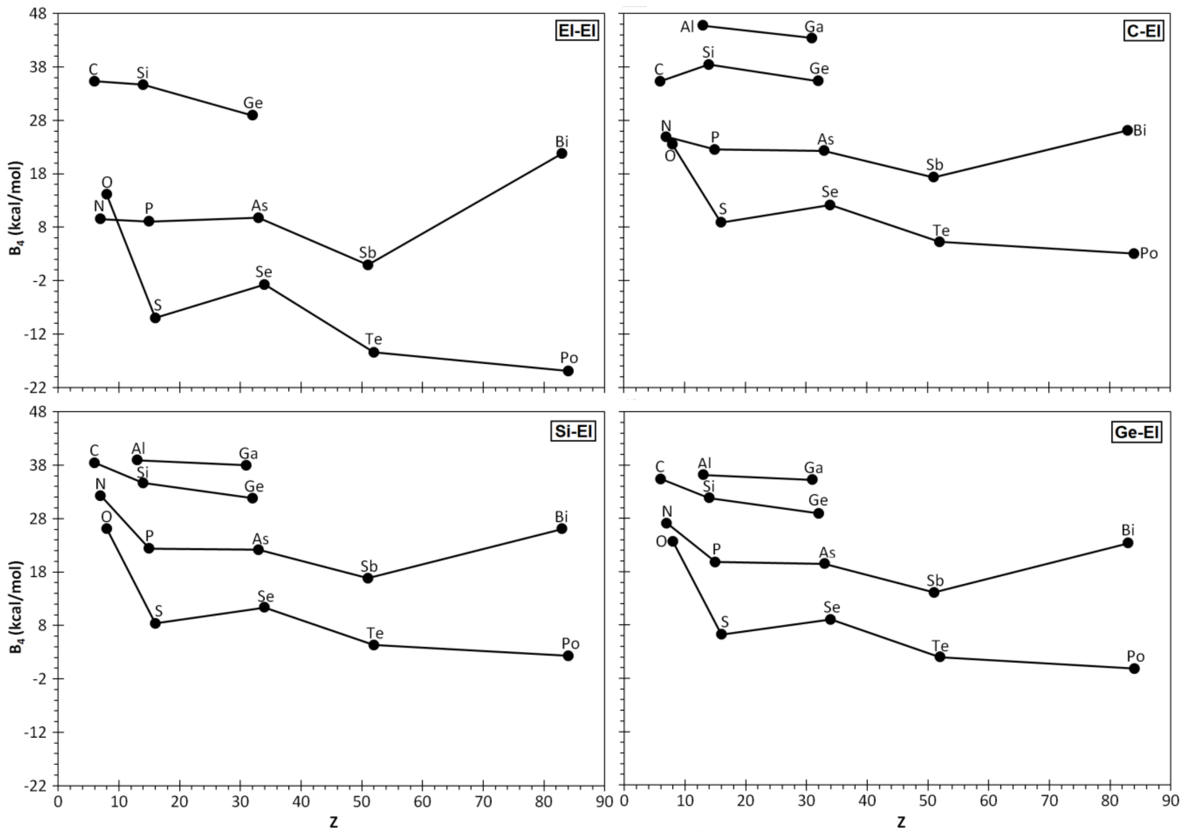

Figure S8. Variation of the calculated bond-strain contributions $\mathbf{B_4^{El\text{-}El}}$, $\mathbf{B_4^{C\text{-}El}}$, $\mathbf{B_4^{Si\text{-}El}}$ and $\mathbf{B_4^{Ge\text{-}El}}$ (kcal/mol) to RSE_4^{ad} with the atomic number.

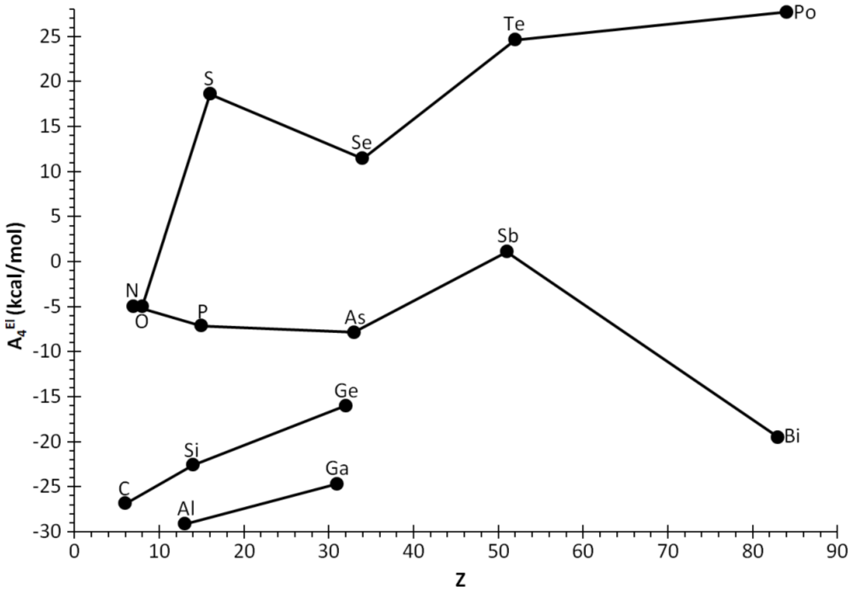

Figure S9. Variation of the calculated bond-strain contributions $\mathbf{A_4}^{El}$ (kcal/mol) to RSE_4^{ad} with the atomic number.

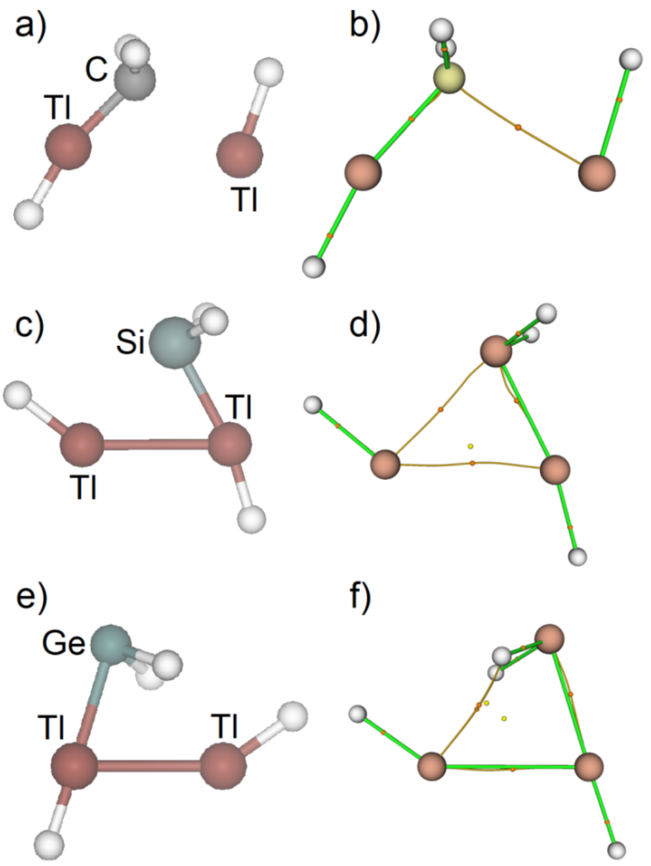

Figure S10. Optimized structures and computed (B3LYP/def2-TZVPP) BCP (small orange spheres), RCP (small yellow sphere) and bond paths for $\mathbf{2}^{Tl}$ (a-b), $\mathbf{3}^{Tl}$ (c-d) and $\mathbf{4}^{Tl}$ (e-f).

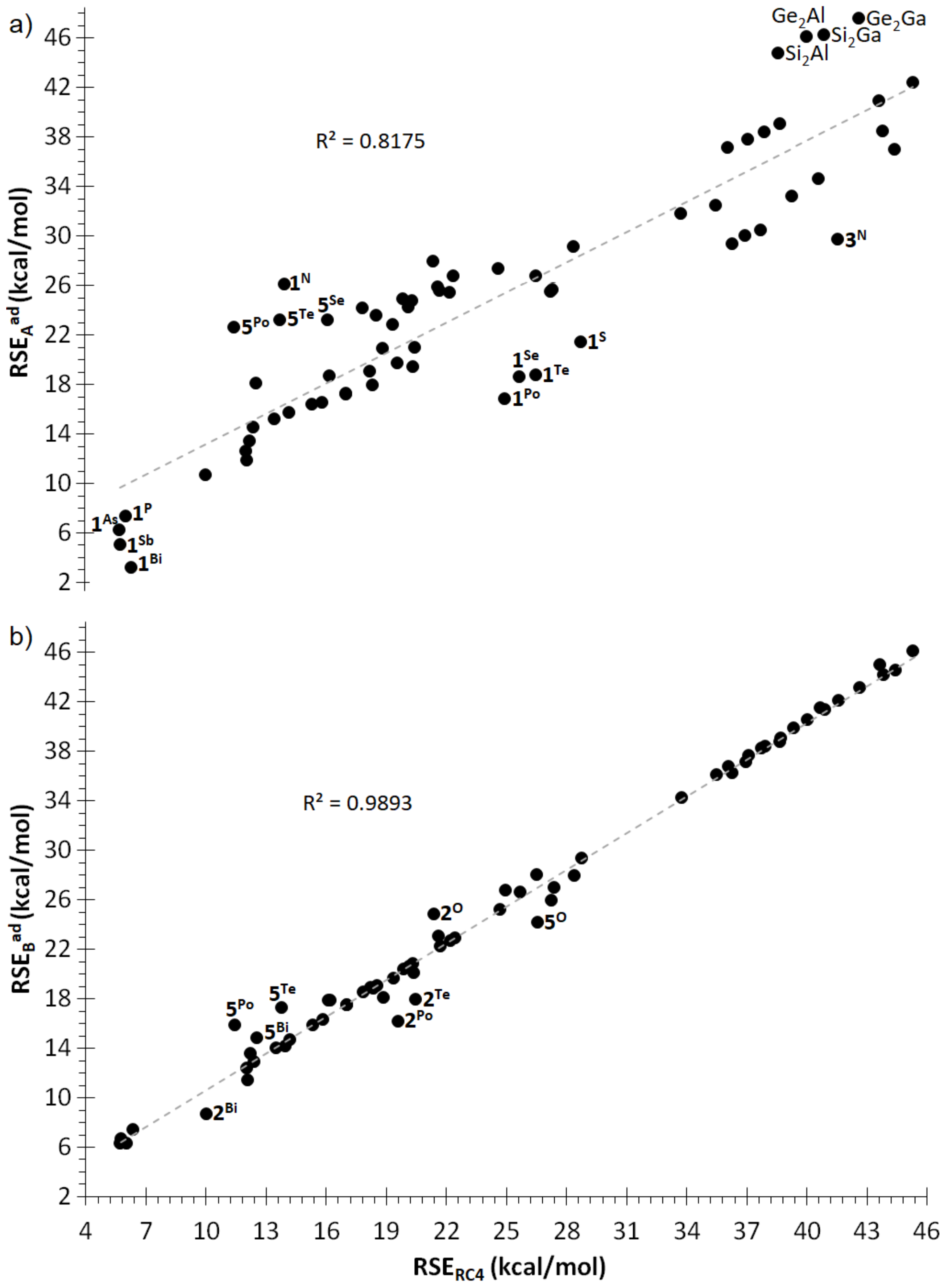

Figure S11. Plot of a) RSE_A^{ad} and b) RSE_B^{ad} vs RSE_{RC4}.

Calculated structures

Cartesian coordinates (in Å), G correction (G-E) and ZPE (in hartrees) for TSs and minima were computed at B3LYP-D4/def2-TZVP. For TSs the imaginary frequency is given. In addition, electronic energies (in hartrees) are quoted using, unless otherwise indicated, the default DLPNO-CCSD(T)/def2-TZVPP(ecp) level.

I^{In} E = -570.253703626904 au
ZPE = 0.01457416 au
G_{corr} = -0.02081143 au

In	1.31594117059649	3.28142460744070	0.44220888730379
In	-0.33462940637632	0.55892880280700	0.42442835954874
In	2.46481275395635	0.71652438475233	1.18136709598568
H	0.95353990752654	4.62990252415258	-0.66593641805003
H	1.34855537142708	1.40926812789347	-0.68935286276061
H	-1.69948953713017	0.29937090295389	-0.69271506202756

II^{In} E = -570.252511655417 au
ZPE = 0.01602681 au
G_{corr} = -0.01936721 au

In	-0.15147065307219	2.37963675174253	-0.51605735665088
In	-1.68809809742684	-0.00307532013133	0.35116987005937
In	1.26810783019636	-0.18953987431442	-0.51172904401203
H	-2.97560474812008	-1.19634146825982	0.02472084977780
H	2.62013435564978	-1.19975331421242	0.01581505671949
H	1.27412817277299	1.44902809517547	0.64387320410627

III^{In} E = -570.254298397284 au
ZPE = 0.01521374 au
G_{corr} = -0.02134765 au

In	-0.08160724752775	1.95346884347064	-0.21905700461097
In	-2.13911859909871	-0.57486454881122	0.17603313312107
In	1.62283648083733	-0.49960207093108	0.04116388904308
H	-1.21194194557578	0.66235949278708	-1.23868403817561
H	-1.14977813255506	0.98880509905918	1.15930600337193
H	3.31736282392000	-1.13004708557461	0.04106091725051

V^{In} E = -570.251746580850 au
ZPE = 0.01560165 au
G_{corr} = -0.02077340 au

In	-0.47835237664818	2.33615321475391	-0.71912198218345
In	-1.62984850939139	-0.46261580418299	0.21768585346118

In	1.38255072900775	-0.68212136745333	-0.71059839735076
H	1.22610814256130	1.30258053756952	-1.05757879817532
H	-2.88134584405905	-1.21364039906102	-0.77812375319150
H	-1.99890573147043	-0.71507674162609	1.92635781743982

VI^{In} E = -570.252074299288 au
ZPE = 0.01423039 au
G_{corr} = -0.01987156 au

In	0.12523850429790	2.37164628500527	0.00943574222619
In	-1.33242320830571	-0.26056234163522	0.34871137069484
In	1.66716053221286	-0.31060663907083	-0.01826884131915
H	0.08691886055052	0.57902824709743	-1.23086519733934
H	-1.39355194675737	-1.99114691171809	0.00375140119425
H	-2.87723624199819	0.53284633032144	0.03808555454323

VII^{In} E = -570.243826357622 au
ZPE = 0.01567749 au
G_{corr} = -0.02070241 au

In	-1.11997945798823	2.09062719088676	-0.23806749554208
In	-2.19981036544012	-0.62149209020858	0.18400430860563
In	0.86111836884541	-0.54967070512244	-0.12114306259299
H	-0.11276033180910	3.54367774940977	-0.50300993602259
H	1.92348084607297	-0.77794173396513	1.26824935480920
H	1.63193295031907	-1.10988366100039	-1.60587439925716

I^{Tl} E = -517.353783823208 au
ZPE = 0.01333544 au
G_{corr} = -0.02463565 au

Tl	1.06327745841055	3.31046233179687	0.44544295916190
Tl	-0.31041532038193	0.26981391323068	0.42459680420406
Tl	2.57046611443196	0.70229327888953	1.23123123198738
H	0.98829053365422	4.70244804384132	-0.74367500273978
H	1.51524586772125	1.33953122260811	-0.71427047956520
H	-1.77813439383609	0.57087055963345	-0.64332551304835

VI^{Tl} E = -517.334264735136 au
ZPE = 0.01271562 au
G_{corr} = -0.02655412 au

Tl	0.81693899452060	2.98017930202974	0.00777225823807
H	-0.21141353425791	4.53677768859744	-0.00897498400449
Tl	-1.80070851761501	0.80343300660299	-0.01965496199960
H	-2.62741491981120	-0.86904960419842	-0.00715975400773

Tl	1.38818635925899	-0.36977576948861	-0.00201260582053
H	3.24973353726557	-0.25281274610297	0.03003001366506

VI^{Tl} E = -517.355948595516 au
ZPE = 0.01503134 au
G_{corr} = -0.02352074 au

Tl	-0.64505653616712	2.00069601517860	-1.72475022029962
Tl	-1.60473901062241	-0.44690502633811	0.20858019604453
Tl	1.65607355520836	-0.29914082627870	0.24129451544265
H	1.24934854766967	1.31041983361677	-1.07357869584189
H	-3.36572877273839	-0.12180710710561	-0.09717780312793
H	-1.66969137335009	-1.87798344907294	1.32425274778221

VII^{Tl} E = -517.339560902717 au
ZPE = 0.01449873 au
G_{corr} = -0.02408877 au

Tl	-1.22483782661418	2.14994180617320	-0.23411818627489
Tl	-2.32736547419602	-0.69468081708417	0.21195004181735
Tl	0.83735195673427	-0.52972876374423	-0.12290024017042
H	0.02300197754893	3.46968035355530	-0.51373974715349
H	1.97767172214938	-0.75248852504590	1.25786816735918
H	1.69815965437762	-1.06740730385421	-1.61490126557772

VIII^{Tl} E = -517.367301798212 au
ZPE = 0.01373712 au
G_{corr} = -0.02446730 au

Tl	0.74977863503192	2.74537734225902	-0.39095986263069
Tl	-2.24707377051932	0.09877654321671	0.72687132605089
Tl	1.45079812605436	-0.38750015778255	-0.39098167576724
H	-0.48914707195946	-0.91271254767499	0.24782151168684
H	1.94243642266473	-2.15315730055582	-0.42086768459137
H	-1.05959548127220	1.84917099053762	0.23590896525157

1^{C} E = -117.671873330993 au
ZPE = 0.08110442 au
G_{corr} = 0.05682064 au

C	-0.43710941234856	-0.07673559524682	-0.00000029679412
C	1.06678346317007	-0.07644518396642	-0.00000009728722
C	0.31492268651494	1.22586407889926	-0.00000005120120
H	-0.94617449322957	-0.37073918926088	-0.90848810554809
H	-0.94617542372376	-0.37073988038577	0.90848827632929
H	1.57590205716387	-0.37031105447073	-0.90845364817334

H	1.57590186451036	-0.37031123536788	0.90845388711557
H	0.31495680545829	1.81364828719419	0.90844841898965
H	0.31495691248435	1.81364864260504	-0.90844838343054

1[Si] E = -870.711449013150 au
ZPE = 0.04924100 au
G_{corr} = 0.02004412 au

Si	-0.85463234470760	-0.31796633751579	0.00000032871016
Si	1.48447211898667	-0.31783508785045	-0.00000058338500
Si	0.31481440491994	1.70820319368781	-0.00000020970521
H	-1.56549212616678	-0.72822629200183	-1.23194421986345
H	-1.56549192206833	-0.72823176291041	1.23194318885236
H	2.19540215240297	-0.72798229553945	-1.23194197006065
H	2.19539971982040	-0.72797814490522	1.23194382696756
H	0.31474909019351	2.52894774017012	1.23193136743433
H	0.31474336661921	2.52894785686521	-1.23193172895010

1[Ge] E = -6230.394444387248 au
ZPE = 0.04530514 au
G_{corr} = 0.01276151 au

Ge	-0.91267118896607	-0.35135283996303	0.00001015509225
Ge	1.54249069661017	-0.35126764571996	-0.00000344135057
Ge	0.31485108049715	1.77445447304310	-0.00000622999510
H	-1.65426145268151	-0.77896824304835	-1.27572815210384
H	-1.65427184539106	-0.77897561402171	1.27573622919161
H	2.28419476808548	-0.77869601917356	-1.27573542451963
H	2.28418446443050	-0.77870279627841	1.27573207583203
H	0.31471895423746	2.63068879119374	1.27562413831759
H	0.31472898317788	2.63069876396817	-1.27562935046433

1[Sn] E = -644.538501201106 au
ZPE = 0.03815489 au
G_{corr} = 0.00211060 au

Sn	-1.23293561523865	-0.25204217277215	-0.00000647429340
Sn	1.61613746412590	-0.67753252538419	-0.00001777449747
Sn	0.56095027037086	2.00263240135741	0.00006036243239
H	-1.83792267963803	-1.07832100226322	-1.38442204942603
H	-1.83766711733719	-1.07840719382954	1.38447014776132
H	2.63423853870681	-0.78894025824189	-1.38436299814616
H	2.63414249023931	-0.78922392845062	1.38437397278488
H	0.14833414566992	2.93996913436891	1.38441348585909
H	0.14852750310107	2.93974454521528	-1.38450667247461

1[Pb] E = -580.129346983212 au
ZPE = 0.03401411 au

G_{corr} = -0.00475865 au

Pb	-1.45017537747934	-0.04322437613822	0.00062491392011
Pb	1.54606126611761	-0.97085584757503	-0.00018295893803
Pb	0.84912194331058	2.08539921206969	-0.00064331487657
H	-1.92308947763093	-1.11830321200772	-1.37886387917979
H	-1.92360388309319	-1.11898845869680	1.37892546074833
H	2.71382816289174	-0.84171521825310	-1.37900569991480
H	2.71368770721838	-0.83942869687991	1.37840336259163
H	0.15536535649472	3.03186206965389	1.37910109925862
H	0.15276876217042	3.03313339782718	-1.37835898360951

1^{N} E = -165.682699685128 au
ZPE = 0.04697269 au
G_{corr} = 0.02295946 au

N	0.86009295108690	1.99083360242462	0.18247539550160
H	0.91253590465151	2.44691263050031	-0.73125676739477
N	0.04066054745039	0.78827281629393	0.05790701288426
H	-0.27383272399313	0.70600702804409	-0.91166523128708
N	1.49266972001304	0.69544459649910	-0.03585901598052
H	1.78559660079128	0.39936824623795	0.89579082627650

1^{P} E = -1024.517655132127 au
ZPE = 0.02930428 au
G_{corr} = 0.00155531 au

P	0.92447006532044	2.41075724264451	0.35233324361215
H	0.87787683438541	2.79316243700334	-1.01435692522916
P	-0.34588248025012	0.56949771156021	0.16594213547320
H	-0.58811685019732	0.66546394651589	-1.22998889436726
P	1.85982438422317	0.44235872566255	-0.09375441735548
H	2.08955104651842	0.14559885661351	1.27721707786654

1^{As} E = -6705.219592326327 au
ZPE = 0.02519072 au
G_{corr} = -0.00601954 au

As	-0.11775915510556	-1.39799620749208	-0.24224020723375
As	1.18605289510283	0.71946096321144	-0.29220033626491
As	-1.18586491682655	0.73543270237661	0.39030550770128
H	0.21596409686790	-1.70938705953664	1.21642728449685
H	1.64925220864042	0.60998256549841	1.15997050720331
H	-1.63151101867904	0.97748579594228	-1.05397770590279

1^{Sb} E = -720.634775201127 au
ZPE = 0.02113441 au
G_{corr} = -0.01273163 au

Sb	0.96756625879819	2.75585377603561	0.46334088514052
H	0.84883856182093	3.08659885963484	-1.21684668950455
Sb	-0.66046885459027	0.39453439567946	0.22469143993154
H	-0.85274668380011	0.61860656723341	-1.46646346215772
Sb	2.17244782667891	0.23065000599915	-0.11722473448360
H	2.34208589109234	-0.05940468458247	1.56989478107381

1^Bi^ E = -643.736489952968 au
ZPE = 0.01911903 au
G_{corr} = -0.01703555 au

Bi	0.98064470528177	2.83933761051700	0.49218483606049
H	0.84125374807838	3.16890822359475	-1.27095361871127
Bi	-0.73615407703995	0.34971520484347	0.24041742491342
H	-0.92682004708124	0.60446245813272	-1.53048478828788
Bi	2.25148305721849	0.17674200378487	-0.12423709986939
H	2.40731561354255	-0.11232658087280	1.65046546589462

1^{O} E = -225.093116725741 au
ZPE = 0.00663585 au
G_{corr} = -0.01659501 au

O	0.31493350471320	1.18346130513462	0.00000000000000
O	1.03026559571404	-0.05537197110822	0.00000000000000
O	-0.40037228042725	-0.05538704402640	0.00000000000000

1^{S} E = -1193.168818030230 au
ZPE = 0.00343573 au
G_{corr} = -0.02308510 au

S	0.31492958208066	1.56558568066174	0.00000000000000
S	1.36084320303691	-0.24643055746816	0.00000000000000
S	-0.73094596511757	-0.24645283319358	0.00000000000000

1^{Se} E = -7200.369015771013 au
ZPE = 0.00192150 au
G_{corr} = -0.02876631 au

Se	0.31492887597830	1.73122450644515	0.00000000000000
Se	1.50482198897884	-0.32924754497944	0.00000000000000
Se	-0.87492404495714	-0.32927467146571	0.00000000000000

1^{Te} E = -802.383595431761 au
ZPE = 0.00128391 au
G_{corr} = -0.03179958 au

Te	1.52976532721989	1.25384345772855	-0.52279956902426
Te	-0.73085668651325	0.96088582684338	1.04514267324455

Te	0.14591817929336	-1.14202699457194	-0.52234310422027

1[Po] E = -711.565240235153 au
ZPE = 0.00093177 au
G_{corr} = -0.03420531 au

Po	1.60578290233557	1.30999684654254	-0.55548394043837
Po	-0.79637357300298	0.99892954515832	1.11072544528007
Po	0.13541749066741	-1.23622410170087	-0.55524150484169

I[Al] E = -524.357085482528 au
ZPE = 0.03616535 au
G_{corr} = 0.00826639 au

C	0.39324645159022	0.36632209662552	0.14661143633910
H	0.24315563561589	0.09441424299798	-0.93020320860300
H	-0.40545122710923	-0.09203197431795	0.72856011011944
Al	2.34918384335688	0.20227622008140	0.05704104835522
H	3.43108982161440	-0.87507757130222	0.49777160036473
Al	0.97080716623959	2.19927455290452	-0.26426151949072
H	0.30931430869217	3.64412043301068	-0.23552046708480

II[Al] E = -524.353983666419 au
ZPE = 0.03506818 au
G_{corr} = 0.00805168 au
-349.00, -127.82

C	0.37780972015407	0.40798348184025	-0.00000009815161
H	-0.18947496653957	0.08065835156878	-0.88167572204402
H	-0.18947473919859	0.08065832015467	0.88167573112680
Al	2.33913334778397	0.13050637561545	0.00000003833526
H	3.31539677376782	-1.12376655404882	0.00000004707256
Al	1.11833636610135	2.24540957161854	0.00000004729694
H	0.51962901793095	3.71786127325112	-0.00000004363592

III[Al] E = -524.346671589201 au
ZPE = 0.03644097 au
G_{corr} = 0.00921597 au
-357.74

C	0.49681765261364	0.32820143652790	0.04785250133064
H	-0.35921472936851	0.97492993191590	-0.19875568718324
H	0.01700278011734	-0.63532267176237	0.22028264365276
Al	2.39733320322195	0.29786986054268	0.26114763536265
H	3.26793151915476	-0.94658606773407	0.64407114993756
Al	1.25140453813349	2.19203907336703	-0.30006572144188
H	0.22007103612726	3.32816643714289	-0.67453352165851

I^{Ga} E = -3887.288830116804 au
ZPE = 0.03611714 au
G_{corr} = 0.00611075 au

C	0.39146248060161	0.26507626808943	0.06446741492983
H	-0.14153002543152	0.49582696858365	-0.87268470548854
H	-0.26769863258950	-0.33266875014830	0.69269746667953
Ga	2.32114813792699	0.09880752774669	-0.04516976177828
H	3.44391366327699	-0.70967369820342	0.67887461227716
Ga	1.23558561648549	2.22850459553040	-0.15527134317180
H	0.30846475972987	3.49342508840150	-0.36291468344793

II^{Ga} E = -3887.285614948599 au
ZPE = 0.03446061 au
G_{corr} = 0.00508134 au
-331.76, -212.86

C	0.33006858171692	0.37993256836188	-0.00000077439217
H	-0.22760465850101	0.05807404638570	-0.88568091891227
H	-0.22760173762149	0.05807291637480	0.88568094165015
Ga	2.34380057752470	0.14353684130331	0.00000015453148
H	3.35354823443594	-1.06660905841528	0.00000079523211
Ga	1.12902522421497	2.24264496825960	-0.00000011216861
H	0.59011929822994	3.72365853772998	-0.00000008594069

2^{Si} E = -619.697699975128 au
ZPE = 0.05879506 au
G_{corr} = 0.03151248 au

Si	-0.81284359581572	-0.18548655168587	0.00000007928194
Si	1.44267633246706	-0.18535425569892	0.00000026813022
C	0.31482667214329	1.34805480501916	0.00000004675614
H	-1.59567623869666	-0.42183577715330	-1.23092293500865
H	-1.59567732674047	-0.42183488901588	1.23092250243981
H	2.22553667461987	-0.42161050895269	-1.23092280979033
H	2.22553848262717	-0.42160976957911	1.23092232987867
H	0.31479133146488	1.96377757377426	0.89589299624332
H	0.31479212793058	1.96377824329234	-0.89589247793113

2^{Ge} E = -4192.803302112713 au
ZPE = 0.05601981 au
G_{corr} = 0.02639133 au

Ge	-0.87145880716313	-0.20845180682467	0.00000322698879
Ge	1.50125157872024	-0.20828264210683	-0.00000164762684
C	0.31479138946026	1.40168599874824	0.00000101370404
H	-1.67450911164130	-0.44566358275953	-1.28373834193013
H	-1.67451767683964	-0.44566869964076	1.28373826716002

H	2.30435138244054	-0.44537345331001	-1.28373378959725
H	2.30435043570750	-0.44537089197066	1.28373200062813
H	0.31485471324040	2.00750389577328	0.90012709586031
H	0.31485055607513	2.00750005209094	-0.90012782518708

2^{Sn} E = -468.884544710694 au
ZPE = 0.05079938 au
G_{corr} = 0.01897886 au

Sn	-1.04423987375185	-0.26627052165586	-0.00000023759444
Sn	1.67407651639108	-0.26613077299735	-0.00000021395685
C	0.31482425075026	1.46628173491299	-0.00000013891099
H	-1.97552970557312	-0.46711199740614	-1.41913241895395
H	-1.97552817152126	-0.46711283919147	1.41913282374353
H	2.60539425378014	-0.46684681216384	-1.41913194518317
H	2.60539268428125	-0.46684716970814	1.41913254389002
H	0.31478753093730	2.07595870412510	0.89888756511950
H	0.31478697470620	2.07595854408471	-0.89888797815365

2^{Pb} E = au
ZPE = au
G_{corr} = au

Pb	-1.75663532857153	-0.38463823568911	0.00000995934708
Pb	2.38673601489195	-0.38594618385775	-0.00000941889283
C	0.31496108996753	0.52875125747090	-0.00000634433343
H	-2.57914305689531	0.32394025071217	-1.45272107627252
H	-2.57913577899617	0.32397237800491	1.45272986922657
H	3.20838408133254	0.32494614679371	-1.45234201178147
H	3.20836402963896	0.32488959782445	1.45236102889771
H	0.31522092901773	1.08098951308444	0.93605547445571
H	0.31521247961431	1.08097414565628	-0.93607748064683

2^{N} E = -149.699281561280 au
ZPE = 0.05882697 au
G_{corr} = 0.03474573 au

C	1.48710501057207	2.30036730511650	2.14723735280753
H	1.95658536449634	1.96923863674822	1.22630703479365
H	1.92424955276528	1.89589499652285	3.05471470423600
N	0.05755032945634	2.49732567471707	2.19404353356379
H	-0.32178969065710	2.42964611435898	1.25185499168455
N	0.99916994056002	3.65916779042047	2.12963206152498
H	1.00487892280704	4.03022657211590	3.07751770138950

2^{P} E = -722.227682956814 au
ZPE = 0.04585540 au
G_{corr} = 0.01924446 au

C	1.57321047141533	2.23050591701638	2.14598107623334
H	1.99332180647659	1.82038876778908	1.23598177409750
H	2.07730127392876	1.89065940734035	3.04218983062594
P	-0.27855309240346	2.31504234127461	2.27977928688413
H	-0.53715298817893	2.21932093581947	0.88559949606084
P	1.10626146369662	4.02685816093759	2.04738964219869
H	1.17336049506507	4.27909155982248	3.44438627389954

2[As] E = -4509.353112861828 au
ZPE = 0.04279081 au
G_{corr} = 0.01378777 au

C	1.60187293420245	2.20727382887742	2.14559292681685
H	2.00755558548685	1.78813121981063	1.23437352664095
H	2.11206609959314	1.88328425744747	3.04298386184595
As	-0.38165309472857	2.24605641611265	2.29926193526613
H	-0.60783625936178	2.15517451913532	0.78824082470656
As	1.15278867721978	4.14211639439846	2.02874476178056
H	1.22295548758811	4.35983045421802	3.54210954294297

2[Sb] E = -519.617451697924 au
ZPE = 0.03940251 au
G_{corr} = 0.00857915 au

C	1.64348491615550	2.17311777724082	2.14500240275717
H	2.04000979070596	1.73450464871667	1.23781209499871
H	2.17083318822788	1.86312584812406	3.03855960136042
Sb	-0.53912202306958	2.13472530619189	2.32807794657957
H	-0.74583387110645	2.04045250144310	0.62283217189554
Sb	1.22637935132701	4.31870399565477	2.00118884724696
H	1.31199807775966	4.51723701262866	3.70783431516160

2[Bi] E = -468.340331512333 au
ZPE = 0.03770056 au
G_{corr} = 0.00519788 au

C	1.66663205358307	2.15414863836437	2.14468537452309
H	2.05545282860597	1.71232349625002	1.23570428737010
H	2.19562725959931	1.85262073127146	3.04018504053102
Bi	-0.60985910779075	2.09276473860000	2.34334933499246
H	-0.80305073949189	1.99038929872215	0.55213312648376
Bi	1.25165486447110	4.39656055399861	1.98659457941826
H	1.35129227102317	4.58305963279335	3.77865563668129

2[O] E = au
ZPE = au
G_{corr} = au

C	0.38976614762792	0.41495025466495	-0.00000002760053
H	-0.10655725039435	0.12839649729822	-0.92673710858718
H	-0.10655716036366	0.12839649686865	0.92673708260045
O	1.02594227470108	1.64729753017701	-0.00000000180907
O	1.77510143842901	0.34982057099117	0.00000005539634

2[S] E = au
ZPE = au
G_{corr} = au

C	0.28857685477833	0.35655490210093	0.00000001330154
H	-0.20977195685332	0.06874578731717	-0.91774803848335
H	-0.20977183855735	0.06874547972897	0.91774805207251
S	1.03003392801547	1.99568599235219	-0.00000014260751
S	2.07862846261687	0.17912918850074	0.00000011571680

2[Se] E = au
ZPE = au
G_{corr} = au

C	0.25631537587940	0.33777997879206	0.00000001466863
H	-0.23817913910622	0.05252231548733	-0.91856614400235
H	-0.23818678238756	0.05253218696815	0.91856687405210
Se	1.00504356883973	2.14108547704489	0.00000546169606
Se	2.19270242677465	0.08494139170757	-0.00000620641444

2[Te] E = au
ZPE = au
G_{corr} = au

C	0.21695909454474	0.31506552760057	-0.00000001515671
H	-0.28091599213371	0.02792178142336	-0.91587171237195
H	-0.28091593538068	0.02792181789360	0.91587170115390
Te	0.97499041452552	2.33704898872103	0.00000002382344
Te	2.34757786844413	-0.03909676563856	0.00000000255132

2[Po] E = au
ZPE = au
G_{corr} = au

C	0.19534850571742	0.30228184326131	0.00000005746572
H	-0.29990241525052	0.01707621487746	-0.91753400874791
H	-0.29990275031140	0.01707634865827	0.91753405886242
Po	0.96198134148198	2.42841887067568	0.00000001554954
Po	2.42017076836252	-0.09599192747274	-0.00000012312976

3[C] E = -368.679896203050 au

ZPE = 0.06932692 au
G_{corr} = 0.04359119 au

C	-0.46148026415279	-0.21782971608891	0.00002914249394
C	1.09114753586773	-0.21799792731498	-0.00001007174956
Si	0.31480935300308	1.46413460633481	0.00005487005194
H	-0.93990106313150	-0.58322857068604	-0.90167311214343
H	-0.94006634427070	-0.58385957917959	0.90141111650535
H	1.56997339029998	-0.58368256521380	-0.90137574590695
H	1.56983378742015	-0.58330868280250	0.90161656729937
H	0.31511495802796	2.26194818609653	1.23913986616065
H	0.31453310693610	2.26170311885447	-1.23919263271132

3^{Si} = 1^{Si}

3^{Ge} E = -4443.833426053528 au
ZPE = 0.04671849 au
G_{corr} = 0.01529817 au

Ge	-0.91374560203929	-0.31824795460067	-0.00000805552567
Ge	1.54360500371554	-0.31808553611666	-0.00000502105612
Si	0.31479002090636	1.73617987792377	-0.00000036523088
H	-1.64872049391762	-0.75154872132917	-1.27617492447427
H	-1.64870471943957	-0.75155420732559	1.27617018550682
H	2.27862921675629	-0.75132339294449	-1.27616591388228
H	2.27861437602733	-0.75129559858613	1.27617643534673
H	0.31476159780490	2.56187328855543	1.22890743730189
H	0.31473506018607	2.56188111442349	-1.22889977798622

3^{Sn} E = -719.925357991886 au
ZPE = 0.04195262 au
G_{corr} = 0.00806832 au

Sn	-1.08861707696242	-0.37084908295712	0.00000089300320
Sn	1.71845781283637	-0.37071484385312	0.00000079067102
Si	0.31480552642303	1.82159549637571	0.00000008587944
H	-1.96256414900778	-0.79811481424656	-1.40758990263017
H	-1.96256958543583	-0.79811478235075	1.40758838488218
H	2.59247862671354	-0.79783315347034	-1.40758939928771
H	2.59248419679127	-0.79783009534638	1.40758840208968
H	0.31474491305119	2.66486972184439	1.22057529363604
H	0.31474419559063	2.66487042400416	-1.22057454824368

3^{Pb} E = au
ZPE = au
G_{corr} = au

3[N] E = -400.742643400270 au
ZPE = 0.04750014 au
G_{corr} = 0.02208248 au

Si	1.60140763725185	2.20726247690394	2.14659432904421
H	2.30922103400966	1.80890369988966	0.91700697856472
H	2.15528146606339	1.57975598093716	3.35952681299137
N	-0.08903060627904	2.56931327577870	2.19036379375521
H	-0.57219849779987	2.58197392147374	1.29696606686855
N	0.90026838904923	3.78809698805544	2.13487407259892
H	0.80280000770479	4.24656074696134	3.03597532617702

3[P] E = -973.259502470611 au
ZPE = 0.03633929 au
G_{corr} = 0.00834876au

Si	1.69510707242685	2.13187101260957	2.14405787346615
H	2.27528670135636	1.58760429200971	0.90241569067971
H	2.36891091085979	1.65781633042879	3.36707335781951
P	-0.51243738903288	2.44928862423817	2.27115827526883
H	-0.67431539535916	2.33898992755321	0.86309030443607
P	0.92681239704271	4.22799816965520	2.06231372120572
H	1.02838513270631	4.38829873350533	3.47119815712398

3[As] E = -4760.393115514242 au
ZPE = 0.03363745 au
G_{corr} = 0.00328236 au

Si	1.71437336454915	2.11627732136873	2.14353549168374
H	2.28594347116813	1.55168029494232	0.90550330308112
H	2.40562225752150	1.65532553390261	3.36334648324432
As	-0.61889620304794	2.37793675359561	2.28853845688401
H	-0.73446295441271	2.27452368829722	0.76682337403127
As	0.97366066622190	4.34712118124516	2.04597787878767
H	1.08150882799995	4.45900231664831	3.56758239228783

3[Sb] E = -770.661515174708 au
ZPE = 0.03072666 au
G_{corr} = -0.00150563 au

Si	1.76616649455881	2.07394016597786	2.14277160075880
H	2.32533630792874	1.47960828633738	0.91048845399104
H	2.48413522958252	1.63212534803793	3.35678031877806
Sb	-0.79047066181334	2.27429918089854	2.31646394149569
H	-0.87246365651474	2.17286095430337	0.60267928493138
Sb	1.03829935912130	4.53585537256173	2.01964361247762
H	1.15674635713669	4.61317778188314	3.73248016756737

3[Bi] E = -719.390410032684 au
ZPE = 0.02925984 au
G_{corr} = -0.00463325 au

Si	1.78359576400481	2.05994180025695	2.14242139808272
H	2.34008318168921	1.44978691032113	0.91466217248679
H	2.51583873404495	1.62417989562100	3.35211898278353
Bi	-0.86354689412786	2.23181402212879	2.33086874101366
H	-0.92530504685523	2.12632795697112	0.53268719331372
Bi	1.06353407357411	4.61623030046703	2.00594683493802
H	1.19354961766998	4.67358620423393	3.80260205738153

3[O] E = -440.446901777604 au
ZPE = 0.02289193 au
G_{corr} = -0.00237829 au

Si	-0.60436215745234	1.61302287748922	-0.36461748145740
H	-0.60443831838602	2.41743759728639	0.86690595570748
H	-0.60443857624368	2.41743794460095	-1.59614064702387
O	-1.39491864926287	0.16769075531462	-0.36461738304749
O	0.18647695134491	0.16784617530881	-0.36461699417872

3[S] E = -1085.742567726871 au
ZPE = 0.02007039 au
G_{corr} = -0.00739922 au

Si	-0.60435952998190	1.75157515981797	-0.36461701118658
H	-0.60445463213123	2.56543302308843	0.86323535210592
H	-0.60445160286056	2.56543217676464	-1.59246978075845
S	-1.69749923892973	-0.04959705174358	-0.36461580318584
S	0.48908425390342	-0.04940795792746	-0.36461930697505

3[Se] E = -5090.532742583647 au
ZPE = 0.01890299 au
G_{corr} = -0.01096545 au

S	i-0.60438629378986	1.78554465543064	-0.36462242834261
H	-0.60443491378404	2.60431806586758	0.86117788925701
H	-0.60444080526137	2.60433077466752	-1.59041483464557
Se	-1.83536410254419	-0.10549645427932	-0.36461606106670
Se	0.62694536537947	-0.10526169168643	-0.36461111520212

3[Te] E = -825.189411296239 au
ZPE = 0.01812073 au
G_{corr} = -0.01343289 au

Si	-0.60437647808684	1.84028885377580	-0.36461772516676
H	-0.60445330734768	2.66602178625546	0.85873543454148
H	-0.60445307550700	2.66602218767538	-1.58797069029657

Te	-2.02270563867318	-0.19457323923111	-0.36461676779655
Te	0.81430774961471	-0.19432423847554	-0.36461680128158

3^Po E = -764.634107583112 au
ZPE = 0.01770254 au
G_{corr} = -0.01532065 au

Si	-0.60438059433715	1.86098989068737	-0.36461757226540
H	-0.60445435410290	2.69285942740427	0.85644071380752
H	-0.60445451558920	2.69285942048696	-1.58567592393574
Po	-2.10816090215288	-0.23176923076639	-0.36461701782208
Po	0.89976961618214	-0.23150415781223	-0.36461674978428

4^C E = -2155.224821199502 au
ZPE = 0.06784327 au
G_{corr} = 0.04082643 au

C	-0.44996634707595	-0.26775462659881	0.00004305863336
C	1.07990086327959	-0.26762078759263	0.00004802440342
Ge	0.31485997836324	1.53393363179213	-0.00008964896010
H	-0.94177813105658	-0.60698412320055	-0.90344412545926
H	-0.94223072549226	-0.60675613242993	0.90366303232795
H	1.57163441197019	-0.60703112045431	-0.90370168284172
H	1.57206460102380	-0.60673738173845	0.90373280315362
H	0.31472354212586	2.32347635881375	1.30356671656174
H	0.31475626686210	2.32335305140878	-1.30381817781902

4^Si E = -2657.272418299892 au
ZPE = 0.04802390 au
G_{corr} = 0.01770803 au

Si	-0.85397098403552	-0.34755387182993	-0.00000008044484
Si	1.48381715072477	-0.34743853631571	-0.00000064920005
Ge	0.31483565941671	1.74343777915745	-0.00000210482066
H	-1.56867115371309	-0.75522399434910	-1.23058408418861
H	-1.56867054653844	-0.75522249196501	1.23058503105992
H	2.19857522619094	-0.75499437264431	-1.23058745392170
H	2.19857238058320	-0.75499232836021	1.23058860729412
H	0.31473944076695	2.59493002208163	1.27612083546260
H	0.31473728660447	2.59493666422518	-1.27612010124080

4^Ge = 1^Ge

4^Sn E = -2506.488632029080 au
ZPE = 0.04039947 au
G_{corr} = 0.00527062 au

Ge	-0.99941178755317	-0.36341196309685	0.00001467765278
Sn	1.65073480179716	-0.51974958556213	0.00000543411799
Sn	0.31090005882275	1.95391301289649	-0.00000708891289
H	-1.69565512950146	-0.90503506227906	-1.26441421222494
H	-1.69572978713128	-0.90506231656315	1.26438854983840
H	2.53606454166063	-0.93550779027616	-1.40625401201114
H	2.53608861073376	-0.93546208443100	1.40626310056656
H	0.09542565429444	2.91408730082266	1.40340588070385
H	0.09538803687716	2.91410748848918	-1.40340032973060

4[Pb] E = au
ZPE = au
G_{corr} = au

4[N] E = -2187.268017217305 au
ZPE = 0.04576399 au
G_{corr} = 0.01907344 au

Ge	1.66078973249123	2.15895782489411	2.14584094380195
H	2.34103849278309	1.77582628677048	0.83537903721032
H	2.19546219428848	1.55433881254938	3.44018698659297
N	-0.14258347330458	2.63433737170660	2.20579270958217
H	-0.56447943653245	2.59470642657953	1.27979954271641
N	0.82521204428558	3.82744211674350	2.12111932001519
H	0.79230987598865	4.23625825075638	3.05318884008098

4[P] E = -2759.812794840453 au
ZPE = 0.03485731 au
G_{corr} = 0.00567783au

Ge	1.73479553907373	2.09955817395961	2.14373105036925
H	2.32260513081430	1.54911635243576	0.84417173559790
H	2.41784303021232	1.61807637101948	3.42385328224371
P	-0.54487866875570	2.47561444625448	2.27175546172737
H	-0.71065445048003	2.36660471148135	0.86429591749473
P	0.89419034559356	4.25425056687867	2.06261887051444
H	0.99384850354180	4.41864646797062	3.47088106205254

4[As] E = -6546.949506522839 au
ZPE = 0.03213792 au
G_{corr} = 0.00066111 au

Ge	1.75018679901390	2.08745534248104	2.14315964888605
H	2.33389189236721	1.51094357309039	0.85021938574846
H	2.45634630020639	1.61562427289075	3.41725007464527
As	-0.65035054699662	2.40189382719615	2.28803418905276
H	-0.77294117941354	2.30158871496972	0.76646720924433

As	0.94273001631980	4.37261648730003	2.04739610811978
H	1.04788614850283	4.49174487207187	3.56878076430331

4^Sb^ E = -2557.222408912957 au
ZPE = 0.02932983 au
G_{corr} = -0.00398522 au

Ge	1.79257996408791	2.05231946524248	2.14215371811601
H	2.37059896052414	1.43998547641024	0.85953500117182
H	2.53282300788410	1.59575793639575	3.40628661750126
Sb	-0.81919176138080	2.29519710416820	2.31482819550717
H	-0.90887645176317	2.19490919037676	0.60136802163973
Sb	1.01212918742370	4.55960560095963	2.02219630593289
H	1.12768652322409	4.64409231644688	3.73493952013108

4^Bi^ E = -2505.953304717215 au
ZPE = 0.02788496 au
G_{corr} = -0.00705653 au

Ge	1.80722581661128	2.03962937572497	2.14119736034972
H	2.38713186728717	1.40946300720266	0.86504083578471
H	2.56436881726869	1.58792267066690	3.40034282630435
Bi	-0.89111636651481	2.25081470537957	2.32845330022827
H	-0.96390016684153	2.14827862378048	0.53081396729068
Bi	1.03873454893182	4.63875353412412	2.00954991052892
H	1.16530491325734	4.70700517312124	3.80590917951331

4° E = -2226.953322642702 au
ZPE = 0.02068263 au
G_{corr} = -0.00585491 au

Ge	-0.60437493951536	1.68996531334515	-0.36461775486158
H	-0.60444091389591	2.46513318282631	0.95114644968394
H	-0.60444071291669	2.46513406481328	-1.68038127206554
O	-1.38366862028623	0.08152308879112	-0.36461680422037
O	0.17524443661419	0.08167970022412	-0.36461716853646

4^S^ E = -2872.283465905326 au
ZPE = 0.01832880 au
G_{corr} = -0.01033289 au

Ge	-0.60436268798248	1.80073779332510	-0.36462429977111
H	-0.60445893739780	2.60877918791538	0.93360579818095
H	-0.60445634227091	2.60879376247972	-1.66284245495714
S	-1.69632449953816	-0.11753193164694	-0.36461136897138
S	0.48792171718935	-0.11734346207326	-0.36461422448132

4^{Se} E = -6877.080125900561 au
ZPE = 0.01720933 au
G_{corr} = -0.01378300 au

Ge	-0.60437934860006	1.82765451932447	-0.36463241350203
H	-0.60445099390528	2.64647202917022	0.92889577690675
H	-0.60443895059850	2.64651249100683	-1.65813851925404
Se	-1.83567243090304	-0.16872049158723	-0.36459784620120
Se	0.62726097400690	-0.16848319791430	-0.36461354794947

4^{Te} E = -2611.743366679356 au
ZPE = 0.01654672 au
G_{corr} = -0.01610622 au

Ge	-0.60438819167714	1.87234314876686	-0.36460501565560
H	-0.60445287806999	2.70904235779200	0.92048853340328
H	-0.60445277919649	2.70901607854002	-1.64972136784528
Te	-2.02342650459433	-0.25360973903328	-0.36462439472315
Te	0.81503960353795	-0.25335649606560	-0.36462430517923

4^{Po} E = -2551.191061049963 au
ZPE = 0.01616877 au
G_{corr} = -0.01792577 au

Ge	-0.60439987318724	1.88938572597581	-0.36460994488048
H	-0.60444947275151	2.73565635099302	0.91661974953244
H	-0.60444981529321	2.73564008710216	-1.64585362260456
Po	-2.10893760301918	-0.28876101239385	-0.36462158022185
Po	0.90055601425116	-0.28848580167715	-0.36462115182553

$H_3C-(CH_2)_3-CH_3$ E = -197.395125268494 au
ZPE = 0.15989301 au
G_{corr} = 0.12973619 au

C	-0.56886122287507	0.19552633728442	-0.10188007460415
H	-1.41730380991068	0.70334134801660	-0.56530827726274
H	-0.63983046233356	-0.86623795193367	-0.35208556090464
H	-0.67889789325831	0.28592651172437	0.98195877469383
C	0.76018500373682	0.78307616831162	-0.57035412803063
C	1.97382844824134	0.09873540314689	0.05547112944286
C	3.30933901672982	0.67908909721373	-0.40561188702272
C	4.51516558391405	-0.01159284730613	0.22689821645950
H	0.79017309723208	1.85299173001416	-0.33646700699646
H	0.82865933078361	0.70981122488031	-1.66134922389858
H	1.90619061166856	0.17082618509598	1.14766034062620
H	1.94525711302896	-0.97238018978945	-0.17807465278451
H	3.33624765709134	1.74891707736194	-0.17224666996671
H	3.37591793471836	0.60566550893934	-1.49645857976627
H	5.45437178141781	0.42341841495268	-0.12088408644355

H	4.49189240838685	0.07533770908949	1.31629737692983
H	4.53160273142793	-1.07686964700229	-0.01756596047125

H_3Si-$(SiH_2)_3$-SiH_3 E = -1452.462112006354 au
ZPE = 0.10093316 au
G_{corr} = 0.06295970 au

Si	-1.90178087175748	0.11832317038629	-0.07405895035647
H	-3.07292670336547	0.79528882583108	-0.68487131495609
H	-1.94791348880586	-1.32215168919651	-0.42742582277585
H	-1.99546394469902	0.24526839080976	1.40137009863676
Si	0.11778504947582	1.04997857776452	-0.82145007479786
Si	1.96330652730806	-0.01691694033190	0.15895710664473
Si	3.99863352172083	0.89246755267247	-0.57196614871737
Si	5.82246031885718	-0.19672302096268	0.42501666239587
H	0.16013331712383	2.49710685950699	-0.48386781910469
H	0.21370444692864	0.92401294259967	-2.29952274519393
H	1.87326346947110	0.10806987433518	1.63780741814623
H	1.92574498987283	-1.46584538459183	-0.17272044012290
H	4.02975400140729	2.33997517834389	-0.23496934810014
H	4.08284784482497	0.76671352636638	-2.05070184494764
H	7.11447246145748	0.38164778424002	-0.02123504148247
H	5.73145489598021	-0.07463072897065	1.90101248815939
H	5.79846149419957	-1.63700283880267	0.06862550657243

H_3Ge-$(GeH_2)_3$-GeH_3 E = -10385.261613650597 au
ZPE = 0.09350623 au
G_{corr} = 0.05090650 au

Ge	-2.07906679449063	0.11790695867043	-0.07877785665417
H	-3.27846111379054	0.83904661172371	-0.72219774731698
H	-2.14563025429131	-1.37486721835711	-0.45000922935646
H	-2.19149447280370	0.24475583104431	1.45150516024382
Ge	0.03773091557705	1.06901458295769	-0.83842704150249
Ge	1.96108243249151	-0.04383801358852	0.17673695497774
Ge	4.08143742644721	0.90577690689525	-0.57904816620775
Ge	5.99802466345622	-0.20995488916575	0.44258580969817
H	0.08731203021602	2.57157818012457	-0.49015792982430
H	0.14133517929952	0.94907360807779	-2.37339019059635
H	1.86729141041712	0.07300118778828	1.71305084530831
H	1.92263762680402	-1.54782351157143	-0.16863007178606
H	4.10850755719597	2.40912790404504	-0.23215400975140
H	4.16628815618773	0.78599592689336	-2.11504792345074
H	7.32573962780388	0.40617438273821	-0.03719078587056
H	5.92207980635298	-0.08400736963952	1.97514258992728
H	5.98912313312692	-1.70537899863631	0.07600932216194

H_3Sn-$(SnH_2)_3$-SnH_3 E = -1075.482750635599 au

ZPE = 0.08027117 au
G_{corr} = 0.03261172 au

Sn	-2.69965014002840	0.10075757527345	-0.07290287092445
H	-4.02986093276749	0.90332763014901	-0.79778457490411
H	-2.79516226652311	-1.56384372795435	-0.46851995170779
H	-2.84187410640314	0.25191102323128	1.62805141703388
Sn	-0.26038144214049	1.16159250200622	-0.93779166034272
Sn	1.95682025351078	-0.09499216753492	0.21847285386211
Sn	4.39679807129705	0.98395770439411	-0.62988856663369
Sn	6.60837574850955	-0.28521482508920	0.51915902526715
H	-0.20017680635483	2.83942608232790	-0.57228025415894
H	-0.14666011450617	1.02843254819503	-2.64700310833671
H	1.84882069254281	0.02343808245930	1.92979923651166
H	1.91755801685808	-1.77260117591554	-0.15364598053868
H	4.42741974953699	2.65813538106256	-0.24521059084804
H	4.49160172066645	0.87250401410062	-2.34164163747803
H	8.08335359004779	0.40677446575368	-0.01444259446388
H	6.54195184457412	-0.15907656094387	2.22682493366337
H	6.61500345117998	-1.94894647151531	0.10880405399885

H_3Pb-(PbH_2)$_3$-PbH_3 E = -968.060926605513 au
ZPE = 0.07339365 au
G_{corr} = 0.02114665 au

Pb	-2.91665819773141	0.09862397756952	-0.06670720403664
H	-4.28770039730205	0.93155618275402	-0.82043103996928
H	-3.02579129147764	-1.62308161579125	-0.46589652485381
H	-3.06524877735785	0.26283573753138	1.69008203579175
Pb	-0.36925214523757	1.18882878276662	-0.97254558459394
Pb	1.95383930257276	-0.11110401921209	0.23285497713561
Pb	4.50990561764678	1.00133891682463	-0.64515468460572
Pb	6.82294444772721	-0.31274148730208	0.54604860447489
H	-0.30673510949557	2.93361232498493	-0.61881458142672
H	-0.25632682806519	1.05582137981067	-2.74526437393158
H	1.83871451037206	-0.00006529654932	2.00814136527820
H	1.91050543932844	-1.85544237870445	-0.13165209253685
H	4.54891467467426	2.74078091322165	-0.26309805702741
H	4.61278271102142	0.89905964521211	-2.42047749725606
H	8.33967392653375	0.44183903116552	0.02438758254442
H	6.73833656186735	-0.22420983914962	2.31253582905703
H	6.86603288492319	-2.02207017513224	0.08599097595610

H_2N-(NH)$_3$-NH_2 E = -277.411113238205 au
ZPE = 0.10371274 au
G_{corr} = 0.07412598 au

N	-0.44409318849188	1.29166310837884	0.41509192581738
N	-1.83564297656491	1.24939439457079	0.62632408148063
N	-2.30707362307828	2.64597542661367	0.60668168879238

N	-3.69583945123765	2.58021618903683	0.88105420737738
H	-3.99182563093950	3.47747387291382	1.23802327297883
H	-4.25123529555364	2.32015649627870	0.06953746119095
H	-2.13662961888174	2.91802026277251	-0.36048229991827
H	-2.05725547795929	0.95492857738802	1.57690275520044
H	0.06554722477376	1.38463433439973	1.28963357428455
N	-0.00020902494519	0.20165668260035	-0.36431699953627
H	0.77972028621037	0.50762078947497	-0.93096309371239
H	0.28171274666800	-0.59684878442829	0.19689855604437

H_2P-$(PH)_3$-PH_2 E = -1708.725078771224 au
ZPE = 0.06551046 au
G_{corr} = 0.02940915 au

P	0.14058358592466	1.47718486976874	-0.21786393595001
P	-2.02908106752103	1.09210292817481	0.13509871033518
P	-2.47801978476988	3.13898249348990	0.93206806682187
P	-4.64160860268454	2.73188779942528	1.34551666275263
H	-4.95086361862474	4.07611665811814	1.67746048112165
H	-5.14361242341948	2.83181901646236	0.02407818713067
H	-2.60015277687361	3.71778812270661	-0.35739483352620
H	-1.93947739012986	0.50682077955699	1.42589445015620
H	0.57858157641370	1.54409050912355	1.12798427452426
P	0.69069925614129	-0.62607998662116	-0.71900630617819
H	2.08799473054362	-0.37818951726832	-0.71794550986304
H	0.69213248500001	-1.17763232293702	0.58849488267495

H_2As-$(AsH)_3$-AsH_2 E = -11176.549204762307 au
ZPE = 0.05688659 au
G_{corr} = 0.01561254 au

As	0.32176191958816	1.52981579082144	-0.34372277488242
As	-2.08148511579046	1.04205457154078	0.05495907148623
As	-2.51249602587715	3.28437823598003	1.01963235050341
As	-4.91547012243256	2.79709777039406	1.45556838517417
H	-5.23548512417403	4.26152195442044	1.74988084077420
H	-5.36563900923692	2.92974914646891	0.00514858038816
H	-2.72409413160157	3.89300669252333	-0.36490751822678
H	-1.88070627685905	0.42722356531215	1.43929166250172
H	0.72446040163910	1.50837783199097	1.12658823160768
As	0.89751128650222	-0.83724416979924	-0.83005480656362
H	2.39499308647138	-0.57458401078355	-0.67873672369864
H	0.78382508177101	-1.32650602886945	0.61073783093587

H_2Sb-$(SbH)_3$-SbH_2 E = -1202.225490830883 au
ZPE = 0.04817906 au
G_{corr} = 0.00445074 au

Sb	0.43214366033904	1.50813472932237	-0.77255072927759

Sb	-2.29182408042886	0.85333778944680	-0.12415573452920
Sb	-2.58091862863358	3.27785556544740	1.37201364617826
Sb	-5.44154843374295	2.96001820058165	1.52786779124247
H	-5.65826652319711	4.62083406332158	1.90577570082763
H	-5.66511518266713	3.27181501568588	-0.14173664595875
H	-2.66759949136223	4.17448576762876	-0.08898292324174
H	-1.85334672702498	0.00018316997922	1.30113974579751
H	0.87366833433660	1.74474565843940	0.86622812246314
Sb	1.18833155253204	-1.26629217538699	-0.75565564210760
H	2.85699518321304	-0.86430900335032	-0.80030622555511
H	1.21465630663628	-1.34591743111590	0.95474802416094

H_2Bi-$(BiH)_3$-BiH_2 E = -1074.049032546147 au
ZPE = 0.04402909 au
G_{corr} = -0.00313017 au

Bi	0.54832643321104	0.35462907315631	-1.72931563474437
Bi	-2.20095037798181	1.66007987438017	-1.88756322521629
Bi	-1.72734516308383	3.40880877848414	0.53707416861997
Bi	-3.78550579689888	1.80059272881269	2.06692284429340
H	-4.25349993460076	3.19259218870735	3.11450623421631
H	-5.08492067472923	2.22451317699390	0.90084178384455
H	-2.96491375432745	4.58356223106011	-0.05694207634804
H	-2.89009356462838	0.32339598647547	-0.88501268114705
H	1.27412064775784	1.87202914077649	-1.09191480467873
Bi	-0.00814593174721	-0.72903051623930	1.04657162943380
H	1.73457822966134	-0.67906442285821	1.50349369185612
H	-0.23447414263245	0.92278311025069	1.72572319987026

HO-$(O)_3$-OH E = -376.506411760668 au
ZPE = 0.03648806 au
G_{corr} = 0.00760336 au

O	-0.07875755442027	3.14201928258423	0.03691379532793
H	-1.01505704926694	3.13619822199523	0.29863204540491
O	0.51959832016166	2.35080497217243	1.05639560233531
O	0.36886904129388	0.97841795496719	0.71406607663452
O	1.34022534263194	0.64903981891904	-0.25369072117615
O	2.57579904730887	0.42924886897558	0.41346640454750
H	2.96540983229082	1.32010031038624	0.40766966692596

HS-$(S)_3$-SH E = -1989.878387660512 au
ZPE = 0.02323400 au
G_{corr} = -0.01068943 au

S	-0.53702687084590	3.75289881632309	-0.15769902931969
H	-1.80265497034833	3.74606554531064	0.30593615314078
S	0.33500879238207	2.63503702383001	1.35268529243020
S	0.09603282347555	0.63275940551804	0.87745777241067

S	1.52367782637641	0.18181115910620	-0.55300102593196
S	3.31715394778172	-0.11047439973744	0.43739434261493
H	3.74389543117845	1.16773187964942	0.41067936465505

HSe-(Se)$_3$-SeH E = -12001.855645213653 au
ZPE = 0.01843935 au
G_{corr} = -0.02071558 au

Se	-0.89243130055547	3.67393764326715	-0.64920324136498
H	-2.08907776783433	3.94882609500908	0.17048342367591
Se	0.51950730023897	3.08985136146754	1.14476012408069
Se	0.14915335068673	0.79715411484038	1.53602020052350
Se	1.39711427134595	-0.29559458062507	-0.13663038880759
Se	3.62777271596215	-0.25124717539822	0.61679595548813
H	3.96404841015595	1.04290197143904	-0.00877320359568

HTe-(Te)$_3$-TeH E = -1338.517895129959 au
ZPE = 0.01523867 au
G_{corr} = -0.02731422 au

Te	-1.47815449794466	2.57327479552761	1.35345763978412
H	-0.04714805493054	2.83323544391921	2.16756920020889
Te	-0.51077647954643	3.34687852451526	-1.08762330815961
Te	0.47433583459741	1.00814593878527	-2.11403384405041
Te	3.03247887056209	0.98510747596864	-1.13121719353709
Te	2.72960615923354	-0.10106009323628	1.36214406479008
H	2.47574514802855	1.36024734452023	2.12315631096400

HPo-(Po)$_3$-PoH E = -1187.135447287965 au
ZPE = 0.01357352 au
G_{corr} = -0.03187194 au

Po	-1.55357440956400	2.58287155162657	1.43581491590309
H	-0.02078191701451	2.84845339638955	2.24899653136755
Po	-0.58782168629976	3.44255618900978	-1.17053762279152
Po	0.44334707405056	0.96001361319126	-2.28154166629204
Po	3.14980348115529	0.95939241973193	-1.21149759569290
Po	2.76913357698384	-0.16956938791647	1.44255181495595
H	2.47598086068854	1.38211164796730	2.20966649254985

H_3Si-SiH_2-CH_2-SiH_2-SiH_3 E = -1201.454271540922 au
ZPE = 0.11076398 au
G_{corr} = 0.07441223 au

C	-1.04286654948414	0.50040173916183	0.52661230119252
H	-0.86475975062476	-0.57834009421422	0.57199545290845
H	-0.45430445233920	0.88162189753333	-0.31345723547522
Si	-3.54093156084643	-0.21577674565754	-1.82984480241991

Si	-2.87082211249242	0.80079836182156	0.17800643502767
Si	-0.41265958462586	1.29054331427671	2.11806435104113
Si	1.85672387976553	0.80759329508615	2.47579258409562
H	-2.74938000639536	0.32441431335593	-2.96513304934122
H	-4.98035111421940	0.02183430187536	-2.10442927005888
H	-3.31002082946766	-1.68181765734697	-1.76368351702278
H	-3.12755218211704	2.26251754982531	0.09929759966617
H	-3.68410710056269	0.26162968483192	1.29889110111926
H	-1.20969080780373	0.79281034543169	3.26945467173842
H	-0.59764380040073	2.76337449677143	2.04743564729986
H	2.05852719207303	-0.66138055373366	2.56774884682471
H	2.35285751340775	1.43025680071774	3.72879776867174
H	2.67284263613314	1.31456925026343	1.34268052473243

H_3Ge-GeH_2-CH_2-GeH_2-GeH_3 E = -8347.674555548365 au
ZPE = 0.10441379 au
G_{corr} = 0.06421430 au

C	-1.02503714309384	0.49528595519291	0.50454222060857
H	-0.85515320613966	-0.58142020038669	0.55157222878888
H	-0.43850345816615	0.88585106332593	-0.32839592990631
Ge	-3.64143591678195	-0.25950475190789	-1.92589623354133
Ge	-2.93835170200007	0.82332746978632	0.14764618490133
Ge	-0.38020988499617	1.31544468919012	2.18006017339036
Ge	1.98071129035117	0.81339697457978	2.55276588379247
H	-2.83306922842965	0.29092940425180	-3.11737830361749
H	-5.13806720503233	-0.02594773838722	-2.20650219284118
H	-3.39492130048687	-1.77882985072245	-1.84399259349939
H	-3.18021899371574	2.34534136160930	0.05827184597037
H	-3.77102248907702	0.28168240649428	1.32940212849197
H	-1.22209115134736	0.77939281272086	3.35791946534229
H	-0.58930353525697	2.84316382642897	2.10722802159914
H	2.19810726027241	-0.71152517705546	2.61248573984722
H	2.48906639258555	1.42736847290903	3.87100428378952
H	2.83536164131469	1.37109358197041	1.39749648688353

H_3Sn-SnH_2-CH_2-SnH_2-SnH_3 E = -899.834075394960 au
ZPE = 0.09275196 au
G_{corr} = 0.04791122 au

C	-1.03964344587634	0.51972979871978	0.51669447161559
H	-0.86977010330942	-0.55622139728472	0.55973497626185
H	-0.45520225128979	0.91995184929034	-0.31193909407115
Sn	-3.91661497161195	-0.39076346530363	-2.26286484094487
Sn	-3.13803578347863	0.86725558973351	0.10888497029898
Sn	-0.31619063706819	1.40207245171618	2.35763117026946
Sn	2.38691119876781	0.81308790437026	2.75653980223354
H	-3.01286162636695	0.20928613340844	-3.59118561883395
H	-5.57907568772642	-0.14925241296751	-2.60388403501295

H	-3.63816598646719	-2.08045581087921	-2.16790089309588
H	-3.39683472655120	2.56270110536382	0.01275845068925
H	-4.05204616648491	0.27328979032803	1.43662720143969
H	-1.26410028624440	0.79606701646026	3.65588646793560
H	-0.56376278192123	3.10008061650899	2.27804700490194
H	2.63106759103917	-0.88333815227352	2.82024869081799
H	2.98272728928569	1.48640073445590	4.21605490207865
H	3.33745974530399	1.42515854835306	1.46689578341618

H_2N-NH-CH_2-NH-NH_2 E = -261.433708849108 au
ZPE = 0.11544273 au
G_{corr} = 0.08549458 au

C	-1.13320422438747	0.49296777695788	0.58835185430315
H	-0.91455052305308	-0.57155946542226	0.65348138054358
H	-0.55568050685110	0.89547434142994	-0.27065512432835
N	-0.74517120586685	1.11936435469703	1.84222959672794
H	-1.00717004588973	2.10290973705045	1.83477489526133
N	-2.56614221874330	0.65846308316131	0.40063462506914
H	-2.80807717410881	1.64684306959265	0.42713281500470
N	-2.96359250155848	0.06635176827580	-0.83229644454232
H	-3.93279142145773	-0.21144347864451	-0.75611231060499
H	-2.86329138416022	0.70067113052077	-1.62166913542164
N	0.65569591653417	0.96431041535981	2.04730683093256
H	1.20057136983392	1.69737507332979	1.59868346142884
H	0.83760995970872	0.98269017369131	3.04191184562603

H_2P-PH-CH_2-PH-PH_2 E = -1406.446213698042 au
ZPE = 0.08282849 au
G_{corr} = 0.04782359 au

C	-1.26720148345567	0.23764819247988	0.83713700236257
H	-1.04977902806093	-0.83083837496089	0.90031845992785
H	-0.70448999900629	0.64901700839912	-0.00165032166870
P	-0.76805153772241	0.99330547868898	2.46802972860412
H	-0.95455317876580	2.34842093488893	2.09279572891293
P	-3.11090528325996	0.40659109594697	0.60869922646967
H	-3.12676162971584	1.80559643618170	0.37536838504154
P	-3.23203244857487	-0.30698014861535	-1.50255993273445
H	-4.61432739184568	-0.02770712262392	-1.64863043512034
H	-2.81904265365084	0.87350149529119	-2.17339533105477
P	1.44512345722469	0.85566993085737	2.21346011122152
H	1.65540043267588	1.98290924126344	1.37727976519840
H	1.75082678415780	1.55728381220255	3.40692190283964

H_2As-AsH-CH_2-AsH-AsH_2 E = -8980.693889286364 au
ZPE = 0.07525414 au
G_{corr} = 0.03591201 au

C	-1.31260154281198	0.15003857443866	0.92254685776916
H	-1.09481490392302	-0.91650406573653	0.97700964739545
H	-0.75939267816969	0.58460214009246	0.09332968486247
As	-0.77271179081795	0.95355845195030	2.66658388964708
H	-0.95995668591257	2.39675321723883	2.20462463242483
As	-3.28183640413998	0.34079330834571	0.67042692942789
H	-3.22703674707196	1.83920878069844	0.38251942590886
As	-3.28500773078150	-0.38670403294915	-1.70311249530165
H	-4.73378885944949	0.04243673430449	-1.91874025049457
H	-2.75139682429650	0.92518235719598	-2.27113327608012
As	1.66774970929137	0.84948942811163	2.22311596140655
H	1.70657026989849	2.04587164132135	1.27669835909777
H	2.00843022818484	1.71969144498780	3.42990492393626

H_2Sb-SbH-CH_2-SbH-SbH_2 E = -1001.219473774489 au
ZPE = 0.06711561 au
G_{corr} = 0.02438068 au

C	-1.39203719793038	0.00710267134541	1.06270884415852
H	-1.18099422116775	-1.06082166843168	1.11621217871699
H	-0.83918944857544	0.44082391059420	0.23332357099311
Sb	-0.75337491276897	0.90053949788404	2.95726451140557
H	-0.99297964819577	2.50198735170863	2.39284308118495
Sb	-3.54556998321023	0.24152845131206	0.74499528336697
H	-3.41038376117314	1.91850188713544	0.41368348946025
Sb	-3.39619136164670	-0.50565516650286	-2.02862923956665
H	-4.95098879339381	0.14928255416660	-2.34035392364797
H	-2.64211487274817	0.96743426668310	-2.47562009894901
Sb	2.02987546121413	0.86773399441252	2.23395360682049
H	1.85175656104753	2.17063080010448	1.13459524107274
H	2.42639821854876	1.94532942958801	3.50879774498401

H_2Bi-BiH-CH_2-BiH-BiH_2 E = -898.659186621367 au
ZPE = 0.06302185 au
G_{corr} = 0.01734218 au

C	-1.41983969330081	-0.02661482922184	1.10795759030383
H	-1.18058685054648	-1.08970551436696	1.12453431343669
H	-0.89079011509700	0.45807568667477	0.29237461783406
Bi	-0.74571895345379	0.86817990954519	3.10164049042730
H	-0.98292557834376	2.54742293097861	2.50947009445462
Bi	-3.67434842530514	0.17572021200875	0.78328246007058
H	-3.53773013359875	1.93112639129497	0.42796370677021
Bi	-3.43532884091579	-0.56989547995062	-2.15547339036604
H	-5.00427959754207	0.23072315753978	-2.52346037530620
H	-2.55586512497314	0.95538160885259	-2.50977678826369
Bi	2.17580366852005	0.84507509642269	2.25617171928703
H	1.87465679019544	2.13433043834552	1.04292672726838
H	2.58115889436131	2.08459837187648	3.49616312408318

HO-O-CH$_2$-O-OH E = -340.765752751550 au
ZPE = 0.06288743 au
G_{corr} = 0.03320208 au

C	-0.78699773960980	1.07766146832083	0.05750861445320
H	-0.39176816499399	0.09151396778735	-0.19413358672645
H	-0.52986868944843	1.83668792576625	-0.68579963542579
O	-2.64288620325492	-0.04909180086605	0.85193451809551
H	-3.19556215991894	-0.52049189991872	0.21190012632230
O	-0.08657180719177	0.40863234122746	2.15958184082948
H	-0.98911937690504	0.31420582855019	2.50582171310798
O	-0.23339764723629	1.53008451934916	1.25591932109225
O	-2.19440921144081	1.07489964978352	0.04217108825152

HS-S-CH$_2$-S-SH E = -1631.369489426792 au
ZPE = 0.04921744 au
G_{corr} = 0.01597499 au

C	-0.86201458488946	0.96400519071971	0.12525932242672
H	-0.43566156935071	-0.01985225147242	-0.05531943704032
H	-0.96066803578030	1.50940457642877	-0.81456189396201
S	-3.38357060471000	-0.73493194146590	-0.09554170171164
H	-3.95152911260297	-0.02102165250850	-1.08995229733434
S	0.82798203059848	0.63554482826985	2.62889745108381
H	-0.19552322401335	0.81026370778945	3.48822770523516
S	0.24255792215618	1.96292087723973	1.16257589655458
S	-2.48828021140782	0.80468013499929	0.94681274474802

HSe-Se-CH$_2$-Se-SeH E = -9640.939243478835 au
ZPE = 0.04451668 au
G_{corr} = 0.00703584 au

C	-0.75411409761661	1.02092550950113	0.01284139960263
H	-0.28507039566051	0.07411128324489	-0.23347345434883
H	-0.89958739463866	1.62717510612652	-0.87904759686582
Se	-3.41355887583505	-0.93518629762017	-0.47770351314423
H	-4.05261319562501	-0.01618211781148	-1.44269081459747
Se	1.17614923546388	0.35782422994911	2.62959189556417
H	0.05460238477728	0.41899185266609	3.58722681502023
Se	0.41717913029919	2.05948370153541	1.20171984675242
Se	-2.47712581116448	0.71254324240849	0.91848208201690

HTe-Te-CH$_2$-Te-TeH E = -1110.251295640017 au
ZPE = 0.04087148 au
G_{corr} = 0.00045407 au

C	-0.83734967953117	1.11096051679387	0.03354972957690

H	-0.32625478159631	0.16941506212523	-0.13675047946300
H	-1.02642360686721	1.62503168646007	-0.90553543746030
Te	-3.75352174426679	-1.19356253909745	-0.46182644419485
H	-4.50831873961600	-0.18158524059880	-1.55358897857199
Te	1.36086187488476	0.51075877078873	2.94669063954340
H	0.13204433628371	0.68274086289563	4.06012211117851
Te	0.44409312891945	2.40246084862027	1.21722258864450
Te	-2.69183817821037	0.78479350201244	1.09651406074683

HPo-Po-CH$_2$-Po-PoH E = -989.130521156118 au
ZPE = 0.03890764 au
G_{corr} = -0.00405551 au

C	-0.80792653374759	1.12580941531326	-0.00179574032563
H	-0.28100143462311	0.18605244040599	-0.12291064222219
H	-0.98990073973242	1.60830991764946	-0.95802221893714
Po	-3.89623028646745	-1.29165754463008	-0.52994717218142
H	-4.68322497744653	-0.21981996292126	-1.67987139470088
Po	1.50546471922840	0.50241688346649	3.03901509353344
H	0.20452497655095	0.65723254648120	4.20764970026304
Po	0.49001342725960	2.52390424641043	1.23068483579333
Po	-2.74842654102178	0.81876552782450	1.11159532877744

H$_3$Si-CH$_2$-(SiH$_2$)$_2$-CH$_3$ E = -950.453527005356 au
ZPE = 0.12285406 au
G_{corr} = 0.08798503 au

C	-2.94866911313870	0.75221767800690	-0.17626178735618
C	1.34055531193629	1.04198200705842	2.63810015565851
H	-3.60822201468215	0.61189793504407	0.68591425538570
H	-2.96378954537394	1.82098609786657	-0.41200300691739
H	1.68407567132513	1.63781268891636	3.48612363005392
H	1.38821512425140	-0.01098561725699	2.92263831923423
H	2.03514015489007	1.19899018424311	1.81052953916025
Si	-3.64860963641418	-0.19899230024521	-1.63582089288921
Si	-0.41184373418366	1.52364506160394	2.14263971148476
Si	-1.19446102795144	0.26798935365266	0.32031210311747
H	-2.80407899764103	0.01277059755178	-2.83664732334290
H	-5.03041772480655	0.24351191903058	-1.95130229196150
H	-3.68010817155968	-1.65074841534207	-1.33329627573756
H	-1.16079586524294	-1.17952080354529	0.66364559057720
H	-0.28295297551668	0.45757572373159	-0.84058782056975
H	-0.46081056166971	2.96772989340861	1.78598872064067
H	-1.33717095422222	1.32909794627494	3.29195738346178

H$_3$Ge-CH$_2$-(GeH$_2$)$_2$-CH$_3$ E = -6310.107826935488 au
ZPE = 0.11793445 au
G_{corr} = 0.07997095 au

C	-3.02652575709845	0.76851096252625	-0.19758486063927
C	1.46363134752809	1.05174989175886	2.72116265562734
H	-3.68342880753534	0.61613373436572	0.66017325292514
H	-3.03900515905425	1.83305033118826	-0.43650143314361
H	1.79887816535398	1.65320008292258	3.56608278134177
H	1.49391287312156	-0.00006370756122	3.00452366059267
H	2.14522534762530	1.20906926094987	1.88556148190185
Ge	-3.74035594316163	-0.23503024440665	-1.73269819222001
Ge	-0.37073028386967	1.56471629687697	2.20717359003484
Ge	-1.18937505729753	0.25578876102082	0.31428775931932
H	-2.85068640474910	-0.00128057419855	-2.96866550939433
H	-5.17790042329536	0.21786049591875	-2.06190553447965
H	-3.75613414272109	-1.74192072244499	-1.41202456364194
H	-1.17128916247883	-1.25006631446225	0.66377230048563
H	-0.25834782191731	0.45042198551471	-0.90455534257988
H	-0.40293243800920	3.06621295141301	1.84107525258499
H	-1.31888039244115	1.36760675861783	3.41205271128514

H_3Sn-CH_2-$(SnH_2)_2$-CH_3 E = -724.218716813057 au
ZPE = 0.10886889 au
G_{corr} = 0.06795045 au

C	-3.20493996526546	0.74466842287096	-0.29268228513359
C	1.78697361440163	1.11772189152813	2.97317575911886
H	-3.85885383698664	0.57351482669532	0.56284167441123
H	-3.21184631530403	1.81317252938209	-0.50957656838624
H	2.11817223952284	1.73057025774816	3.81054465677236
H	1.80427158833629	0.06929008634611	3.26643406247539
H	2.46128297284286	1.26543861600520	2.13116088680693
Sn	-4.01362238124228	-0.30437740498563	-1.99833492029906
Sn	-0.22615829155539	1.68835146208383	2.40999458290299
Sn	-1.19023232093599	0.17506576939386	0.26720280275108
H	-3.03491389450292	0.00361180886968	-3.37096862535212
H	-5.61664587141531	0.21692434514332	-2.32069142498505
H	-4.02322620136332	-1.98872099097717	-1.68191467249099
H	-1.20125228006645	-1.50105830156614	0.65522566467582
H	-0.18058033781585	0.36349969459255	-1.11348522439918
H	-0.23514932497908	3.35880402300801	1.99889691586529
H	-1.25722345367085	1.49948291386166	3.77410672526626

H_2N-CH_2-$(NH)_2$-CH_3 E = -245.439285606381 au
ZPE = 0.12694059 au
G_{corr} = 0.09733543 au

C	-2.42492687762263	0.50442579339605	0.46685936090175
C	0.29263592411856	0.81010236060084	2.09867696992108
H	-2.24607160070758	-0.40970352468803	1.05320604305005
H	-3.14561840944148	1.11432085054418	1.02436578016719
H	0.72169328543221	1.25089033794977	3.00068359776552
H	-0.19514885522967	-0.12373072456519	2.38358018179979

H	1.11464133596257	0.56841434499007	1.40544266062868
N	-2.85598848980605	0.09172137081982	-0.85777040397728
H	-3.57529657668751	-0.61757631850723	-0.80575650834801
H	-3.21937195421562	0.88185097807980	-1.37783301461106
N	-1.23174085424510	1.32228104377879	0.29513303098436
N	-0.68510637937789	1.73269137518303	1.53804998915231
H	-0.55453185650496	0.79663691002116	-0.25903684747265
H	-0.27312283167483	2.64626212239692	1.40125423003823

H_2P-CH_2-$(PH)_2$-CH_3 E = -1104.181213273127 au
ZPE = 0.10217352 au
G_{corr} = 0.06838643 au

C	-2.91751162707535	0.31554059284738	0.36355036154964
C	1.15285981907414	1.04075968673632	2.42104860845815
H	-3.40434830166108	-0.23854420260105	1.16919139119874
H	-3.16822942897243	1.36960292872119	0.47586446770488
H	1.53607611846252	1.71203555276381	3.19109973520329
H	1.38569597457289	0.01760885670384	2.71748804627847
H	1.65727612274829	1.25929072315340	1.48005337273639
P	-3.54821673595084	-0.39108596334627	-1.24634308942456
H	-4.81927373308079	0.24412055247686	-1.23509737188697
H	-2.98004842586563	0.58786970072254	-2.10363191693175
P	-1.08885493492311	-0.00457659400886	0.52824408924910
P	-0.69555020483011	1.22887772876386	2.33683845744227
H	-0.65752638687876	0.91199182647693	-0.46730332448300
H	-0.73030239561966	2.51509553059001	1.73585224290533

H_2As-CH_2-$(AsH)_2$-CH_3 E = -6784.862855699482 au
ZPE = 0.09640129au
G_{corr} = 0.05923316 au

C	-3.01595129302939	0.25387454807616	0.35267385503003
C	1.30839374737378	1.09612045091412	2.49398339437371
H	-3.54446466620438	-0.26939478862495	1.14939359952492
H	-3.19136229014701	1.32165670735876	0.45040876414163
H	1.70994929370594	1.80908484197353	3.21353206499732
H	1.59740710820330	0.09067564323464	2.79674917788010
H	1.71373745691640	1.31024611100007	1.50740356477497
As	-3.72508893507658	-0.43988626197280	-1.37945165210702
H	-5.00199192810302	0.40258354095435	-1.36488191017374
H	-2.99784133045273	0.58710034529645	-2.24519715712837
As	-1.08371984446964	-0.19564545575360	0.54335204961084
As	-0.68031567173407	1.21715821474780	2.53573590741482
H	-0.60550892710179	0.83533187382723	-0.47828619213690
H	-0.76119685988073	2.54968114896820	1.79143960379765

H_2Sb-CH_2-$(SbH)_2$-CH_3 E = -800.251786317608 au

ZPE = 0.09005274 au
G_{corr} = 0.05024921 au

C	-3.18007767734495	0.13099126466374	0.33964604258492
C	1.54845574902574	1.20128154041495	2.59885205951518
H	-3.77020919062254	-0.36160393606632	1.11234127822301
H	-3.25930634319639	1.20794529301279	0.46010303583331
H	1.97297100075020	1.97637179616527	3.23508388160620
H	1.92351674656022	0.23065144851639	2.91938444926601
H	1.82844758605120	1.38218671579145	1.56400331213617
Sb	-4.00805490208171	-0.46522219412210	-1.59401899902723
H	-5.27227213991139	0.70116095721824	-1.56982302186965
H	-2.99530379503964	0.60081590783044	-2.47914408770619
Sb	-1.10494865167372	-0.51619899240764	0.59995606812608
Sb	-0.62949961216835	1.22265656217373	2.83451018737938
H	-0.49666309124269	0.62453864356167	-0.52967170157151
H	-0.83500981910588	2.63301191324734	1.87563256550426

H_2Bi-CH_2-(BiH)$_2$-CH_3 E = -723.324717288483 au
ZPE = 0.08680161 au
G_{corr} = 0.04476780 au

C	-3.22457561373357	0.06861280355002	0.33324444755867
C	1.63542189882180	1.27741080712728	2.58954904474647
H	-3.86998210670196	-0.41195967444985	1.06825424808655
H	-3.23831871797316	1.14432326491323	0.48023054718361
H	2.03824813217081	2.09644296061197	3.18217834603778
H	2.10578174748507	0.34162311507862	2.88549260576706
H	1.79644198989776	1.46372741072940	1.53147953057148
Bi	-4.06512440302325	-0.41826735082239	-1.73357529095505
H	-5.37197131556485	0.82239144333325	-1.62522340951078
H	-2.98432548594982	0.77076987734167	-2.54190774610007
Bi	-1.10721914325681	-0.72887734516874	0.66011568546386
Bi	-0.61058754074785	1.11987082534712	3.01577489821730
H	-0.39066416880095	0.46647523171943	-0.47784307316796
H	-0.99107941262313	2.55604355068893	1.99908523610103

HO-CH_2-(O)$_2$-CH_3 E = -304.955115834813 au
ZPE = 0.08788303 au
G_{corr} = 0.05827928 au

C	-2.42723815524242	0.99730880650777	0.29551440708093
C	0.45105676275566	0.92613760005778	1.89323116495482
O	-2.94858069999884	0.48672898983386	-0.88345313186864
H	-3.32237921755066	-0.38531240069613	-0.70588764217425
O	-0.92378067775128	0.60116430710664	1.98465124596198
O	-1.32519730396063	0.16057307102290	0.65878389623805
H	-3.14468655565258	0.99881872098391	1.12372239273399
H	-2.08379979754795	2.01327425274785	0.08707832378329
H	0.72989563295168	1.23239652598669	2.90299052107937

H	1.04264135688023	0.05686084975906	1.59533159648528
H	0.62312233511683	1.74956238668964	1.19461260572513

HS-CH$_2$-(S)$_2$-CH$_3$ E = -1272.872308647193 au
ZPE = 0.07688104 au
G_{corr} = 0.04393152 au

C	-2.64339916900483	0.44367695923059	0.49438934453510
C	0.65833661539714	0.54886701608624	2.35700731112633
S	-3.61052941641648	-0.10165046504450	-0.93829506771637
H	-4.45142412762532	-0.90061591466131	-0.25899868239310
S	-0.90836027485275	-0.20240566269273	2.91023881017240
S	-1.70419895322305	-0.97362270649882	1.18180787510269
H	-3.27628643063884	0.87219147037418	1.26722171212686
H	-1.96057134031382	1.20455610142110	0.12030904747996
H	1.15167740360211	0.91949910480317	3.25719799176653
H	1.28858586448318	-0.19763367423007	1.87838461990311
H	0.47782522859274	1.38201941121214	1.68030815789650

HSe-CH$_2$-(Se)$_2$-CH$_3$ E = -7280.045219159149 au
ZPE = 0.07331377 au
G_{corr} = 0.03690072 au

C	-2.53024273691130	0.99700158399166	0.02874509247258
C	0.90182582393465	1.10828249306034	2.22313119256381
Se	-3.47898065857228	0.50796848320218	-1.61645139918935
H	-4.44173047098218	-0.37915417358254	-0.94193053753404
Se	-0.78843767315294	0.24965796482648	2.77090003635244
Se	-1.54436911458135	-0.57253108045760	0.71168137219553
H	-3.22374293647641	1.35394436649430	0.78217259918412
H	-1.83450301744033	1.78326473871366	-0.25009931418752
H	1.36445477905813	1.47829237095859	3.13804072817945
H	1.54649894476085	0.37342023305619	1.74982643793328
H	0.70028074036325	1.93736612973673	1.55055917202962

HTe-CH$_2$-(Te)$_2$-CH$_3$ E = -882.021936295803 au
ZPE = 0.07024264 au
G_{corr} = 0.03140998 au

C	-2.66818533746838	1.01945105341112	-0.05603706345681
C	1.14100132942622	1.15646264069318	2.35696671150207
Te	-3.70230677077144	0.54869356282541	-1.89491180050821
H	-4.76271164409186	-0.48678171070462	-1.14015187822936
Te	-0.70973633794340	0.24514975933404	3.04163785132631
Te	-1.59182132936892	-0.72246368830546	0.65817417687550
H	-3.37834788344419	1.35593411633426	0.69097468310359
H	-1.96681596909145	1.81285689365370	-0.29740502208999
H	1.64347160036360	1.54915252952910	3.23993877648232
H	1.75340017784187	0.39677833359856	1.88131996108237

H	0.91310584454807	1.96227961963069	1.66606898391213

HPo-CH_2-$(Po)_2$-CH_3 E = -791.175351786603 au
ZPE = 0.06860795 au
G_{corr} = 0.02780540 au

C	-2.73607672140492	1.02786287592754	-0.08617984240644
C	1.26203794308131	1.18267792410634	2.41471334414442
Po	-3.75866328731051	0.52556671345774	-2.04053581983530
H	-4.94609608072018	-0.48407643789053	-1.24145923362934
Po	-0.66219774168883	0.23685238515232	3.18168584139309
Po	-1.60502703071853	-0.79231360945491	0.64532314617092
H	-3.48007680801746	1.33585200978209	0.63904266544172
H	-2.04350910000531	1.83504381222367	-0.30394671581225
H	1.78190855822396	1.58191452414321	3.28366776765333
H	1.85258209734328	0.41083395365564	1.93262251538139
H	1.00617185121733	1.97729895889686	1.72164171149837

H_3C-CH_2-SiH_2-CH_2-CH_3 E = -448.416252117456 au
ZPE = 0.14738363 au
G_{corr} = 0.11554621 au

Si	-0.90133202490285	0.36075462792697	0.38246098545162
H	-0.67216601317412	-1.10933507166042	0.44773772140170
H	-0.09395772094483	0.88006639865588	-0.75616889256621
C	-3.25719930793083	0.06268445441603	-1.23490348117759
C	-2.72618122442911	0.69499529234486	0.05858017843626
C	-0.30937208749291	1.15741883212346	1.98304562848256
C	1.17360177300410	0.91145640509451	2.29139194826099
H	-2.71888406103841	0.43481345296859	-2.10969159540340
H	-4.31722803626089	0.28132098550289	-1.38399777315094
H	-3.14480443217813	-1.02391173444257	-1.22161352200698
H	-2.87774853980981	1.77952421157875	0.03578240248283
H	-3.29987754458157	0.33036698156463	0.91742993256956
H	-0.93054070359013	0.78259710013876	2.80358470960277
H	-0.50726509167345	2.23313706365358	1.92471827441945
H	1.38845089139536	-0.15557542614058	2.38536180319097
H	1.47535576385474	1.39078192943214	3.22563415899815
H	1.81500972975288	1.30395479684250	1.49887693100822

H_3Ge-GeH_2-SiH_2-GeH_2-GeH_3 E = -8598.699525367001 au
ZPE = 0.09498095 au
G_{corr} = 0.05336274 au

Si	-0.89047559964943	0.41413114682698	0.35530400467986
H	-0.65728172200737	-1.05225112519485	0.39591894846874
H	-0.09100919293175	0.97076676371551	-0.76585451849865
Ge	-3.98971363998780	-0.29091613354206	-2.08063190678443
Ge	-3.21050106998648	0.82542325248644	-0.05194537530819

Ge	-0.14754691267595	1.37853663316729	2.41309662259652
Ge	2.20968127793077	0.90561257144588	2.84426547852993
H	-3.20688925457469	0.22554566246604	-3.30223445069118
H	-5.49063712646846	-0.03787419276058	-2.31644845210003
H	-3.76040054560616	-1.80930726906768	-1.96423197151866
H	-3.45301314265810	2.34443218656438	-0.17350748183690
H	-4.03884268685827	0.32424745937600	1.14997351783124
H	-0.99836144350556	0.82095474913618	3.57373747784072
H	-0.36538089170264	2.90579050756115	2.37836598735682
H	2.44178225644191	-0.61637159207187	2.88410560498181
H	2.66428618640534	1.50710390434603	4.18732876776458
H	3.08016487783473	1.49922577554513	1.72098715668777

H_3Sn-SnH_2-SiH_2-SnH_2-SnH_3 E = -1150.869203122093 au
ZPE = 0.08397598 au
G_{corr} = 0.03848549 au

Si	-0.90405120796911	0.40619410510647	0.37770538137587
H	-0.67153618244395	-1.06059845933071	0.42522746823082
H	-0.09972245755911	0.94808226869193	-0.74795457457358
Sn	-4.27616930711940	-0.39959296028979	-2.43489195607953
Sn	-3.40326437388341	0.84331317721889	-0.08671827340664
Sn	-0.07976635935214	1.45549250712077	2.58493472188348
Sn	2.62430374099643	0.92510884612654	3.05510048929400
H	-3.39272046648284	0.18258837332721	-3.78326857266554
H	-5.94151871427248	-0.11137419101303	-2.72129258152371
H	-4.03613738876507	-2.09379218567692	-2.33613245536901
H	-3.67350090737675	2.53562552230260	-0.19346694268357
H	-4.33301274850704	0.27490226153976	1.24045806614213
H	-1.01935561248908	0.84559702152082	3.88639593747562
H	-0.33712407517143	3.15250391086896	2.53884491990818
H	2.89591204583303	-0.76600493173333	3.11882291286677
H	3.15712113831074	1.60901167994883	4.53379779137619
H	3.58640424625169	1.56799335427098	1.79066707774844

H_2N-NH-SiH_2-NH-NH_2 E = -512.512764208024 au
ZPE = 0.10419216 au
G_{corr} = 0.07305007 au

Si	-0.94877147005267	0.33936598768216	0.39720890042910
H	-0.71233836923442	-1.02213702480439	0.93734803074947
H	-0.27140258535362	0.44310765516448	-0.91878625475316
N	-0.42918502969290	1.42719292147868	1.63504017333465
H	-0.62395187544042	2.41762353026850	1.55968307960660
N	-2.59070983746400	0.79621635585407	0.11222162258439
H	-3.22191538034741	0.94251574090485	0.88991500220888
N	-3.20410356049461	0.41244015814097	-1.10997010409627
H	-3.83412491506586	-0.37162040033791	-0.97765515585977

H	-3.72834815871958	1.19000918726900	-1.49348827966746
N	0.71036473655317	1.08480596904776	2.41062119463208
H	1.52699427161087	1.61269536309075	2.12147396414757
H	0.53169821370155	1.27220253624103	3.39016211668384

H_2P-PH-SiH_2-PH-PH_2 E = -1657.484762739489 au
ZPE = 0.07306181 au
G_{corr} = 0.03677146 au

Si	-1.19779874380953	0.07163799560743	0.80184371169104
H	-0.95540856800429	-1.38104522199867	0.95558577776874
H	-0.40088903385245	0.55807265522530	-0.35609624031147
P	-0.62196258683616	1.08465144777709	2.74197413536952
H	-0.89324228228461	2.39903682654721	2.27909011850734
P	-3.42167819436230	0.38239718392169	0.51981571278397
H	-3.32897512166031	1.78942905158391	0.35523785248546
P	-3.48683958490725	-0.26466669254394	-1.61904397514438
H	-4.81675482854317	0.16916557639420	-1.85317391190664
H	-2.90104099877984	0.87514587994988	-2.22510039791724
P	1.58784424379012	0.99574226207047	2.41531184231162
H	1.72018335884669	2.01600545256327	1.43996354316866
H	1.92076838040316	1.84884556290214	3.49836612119334

H_2As-AsH-SiH_2-AsH-AsH_2 E = -9231.739106333796 au
ZPE = 0.06600607 au
G_{corr} = 0.02550560 au

Si	-1.23913802321730	-0.00659482709109	0.87827881567354
H	-0.98013425527046	-1.46190548733075	0.99855464353885
H	-0.45064161179554	0.50619727011867	-0.27244761573988
As	-0.61435438527747	1.01078101506349	2.93557365660439
H	-0.91652909091826	2.41170161844911	2.41075195425508
As	-3.58011364123182	0.29054675995054	0.58109755935662
H	-3.42891329500864	1.79637783772720	0.38370443673301
As	-3.52326977305878	-0.32780108720330	-1.82678785451964
H	-4.91486627214645	0.24357211689739	-2.08780504633072
H	-2.84571641508331	0.95123998535444	-2.30610628340417
As	1.81055166724300	1.01086991969774	2.38682526564760
H	1.74159980542782	2.13431123453699	1.35842763988728
H	2.14573133033730	1.98512162382954	3.51370711829800

H_2Sb-SbH-SiH_2-SbH-SbH_2 E = -1252.266175060422 au
ZPE = 0.05843701 au
G_{corr} = 0.01460440 au

Si	-1.34297599115638	-0.14337172951472	1.05199637893328
H	-1.11736539522537	-1.60079502030869	1.23821381342925
H	-0.52514678114244	0.28090360662606	-0.11521343308392
Sb	-0.60859330527280	1.04934980106115	3.23217483579254

H	-0.91123039123925	2.58205596990762	2.52804908889550
Sb	-3.87654376274633	0.21111027956781	0.63928172304893
H	-3.63267219330705	1.89161913625307	0.40875426551087
Sb	-3.55203597244304	-0.41478823700868	-2.14907932619794
H	-5.08139193280627	0.26326750149811	-2.53308719966338
H	-2.76745066720444	1.06911820044195	-2.48813039431304
Sb	2.14091777544951	0.98862615389117	2.39065113313927
H	1.90171370023722	2.19331815748787	1.19726191460716
H	2.57698095685670	2.17400416009724	3.55290148990144

H_2Bi-BiH-SiH_2- BiH-BiH_2 E = -1149.712600096972 au
ZPE = 0.05454570 au
G_{corr} = 0.00762533 au

Si	-1.42789335603109	-0.17940656490578	1.17396232074087
H	-1.22334739438406	-1.63905962454664	1.39419182439549
H	-0.58878960580277	0.18294417136146	-0.00174155491477
Bi	-0.59428310068953	1.09624420045832	3.38052154655399
H	-0.89708183927928	2.67232691332440	2.57687101348759
Bi	-4.03107625756406	0.19966850891629	0.64830665662065
H	-3.72290589069144	1.95115928035379	0.40511389850251
Bi	-3.44969482355256	-0.43654294688941	-2.26675240175117
H	-5.08014012236686	0.14549231313273	-2.76465631963444
H	-2.76755246383680	1.19932269776998	-2.54394578560891
Bi	2.24361085401621	0.94414944851642	2.30099569023184
H	1.92770396953794	2.26724808860791	1.13139967922453
H	2.81565607064438	2.14087149390044	3.51950772215178

HO-O-SiH_2-O-OH E = -591.864711173754 au
ZPE = 0.05249962 au
G_{corr} = 0.02163113 au

O	-3.23124431609003	-0.58971720529294	2.86795303202280
H	-3.71839378361232	-1.38853936985090	2.61543868481614
O	1.52027522636714	-0.73667350870732	2.05123204114265
H	1.94608143908933	0.12311600839322	1.91488689628669
O	-2.28135283211190	-0.49715410964720	1.75279688391125
O	0.26237877113587	-0.52520608428891	1.32534290580429
Si	-0.83483642001792	-0.68296405573838	2.58901336871888
H	-0.75735202337483	-2.02125712251359	3.19994127245929
H	-0.64598690138533	0.39354627764603	3.57667051483798

HS-S-SiH_2-S-SH E = -1882.441124820322 au
ZPE = 0.03998468 au
G_{corr} = 0.00531366 au

S	-3.69685864333637	-1.63005261696372	2.62602859002520
H	-3.61622428527345	-1.62308310930050	1.28187491885931
S	1.77355962545757	-1.37074666975591	1.66019070116352

H	2.40011176980806	-0.18091221247491	1.57411407973002
S	-2.90529354912991	0.27555278598150	2.97280286586193
S	-0.13992835682378	-0.73104576205035	1.10807946561238
Si	-0.82051172943387	-0.24245337258975	3.09264006590781
H	-0.60599387683105	-1.39223027849513	3.99025998795995
H	-0.12929179443717	0.97012206564879	3.58728492487983

HSe-Se-SiH$_2$-Se-SeH E = -9892.009655286489 au
ZPE = 0.03573420 au
G_{corr} = -0.00307572 au

Se	-4.11890197982972	-0.94249501948307	3.31868971982015
H	-4.10585491260597	-2.37880676062900	2.97678757863623
Se	2.48909139971395	-0.14541090402336	2.35882782712743
H	2.71228065184494	-1.55398996091300	1.97661397575691
Se	-2.94112495031978	-0.21328692472981	1.39292305409400
Se	0.68207336566916	0.23711741876946	0.87001680486199
Si	-0.88658550688182	-0.46165479923257	2.40752705979363
H	-0.65992215244958	-1.88174495999712	2.74841156602324
H	-0.81511129940274	0.36940196846602	3.62521822264417

HTe-Te-SiH$_2$-Te-TeH E = -1361.317513436409 au
ZPE = 0.03227183 au
G_{corr} = -0.00945013 au

Te	-4.18965378834856	0.41431869416423	2.50244788727966
H	-4.68508023723635	-0.37208814131343	3.88670130132544
Te	2.34541670062279	0.76502500752861	2.85869048729991
H	3.32218420260227	-0.27337807493323	1.99364365001298
Te	-3.08328624282570	-1.81628505143784	1.35243580266081
Te	0.20076259628357	0.57921328104610	1.15567022230992
Si	-0.78876495864839	-1.41502640451089	2.31917123519960
H	0.00761021929745	-2.63633018093516	2.04994123741586
H	-0.86961933174702	-1.17029829960839	3.77457377649571

HPo-Po-SiH$_2$-Po-PoH E = -1240.198348412611 au
ZPE = 0.03048172 au
G_{corr} = -0.01385836 au

Po	-4.27939754256530	0.50073150370250	2.54570256107023
H	-4.85174129833425	-0.35124697061279	3.97069944577162
Po	2.47837257025273	0.87253890497765	2.88193804235662
H	3.52908374110589	-0.23066874938266	2.00814398568446
Po	-3.16928407726976	-1.88273224496228	1.28640677403101
Po	0.21932445519672	0.57062551439161	1.05995765963847
Si	-0.79060666029559	-1.47910577309130	2.30829170865489
H	0.00904165332433	-2.70655183766001	2.07064381107295
H	-0.88522368141472	-1.21843951736272	3.76149161171964

H_3C-SiH_2-$(CH_2)_2$-SiH_3 E = -699.431773442619 au
ZPE = 0.13368220 au
G_{corr} = 0.10038949 au

Si	-2.86809368783929	0.84217537125337	-0.03518198009671
Si	1.06658816299507	0.92204738043465	2.34884517100503
H	-3.81453633833657	0.63147984674079	1.09293775052214
H	-2.93657900459696	2.27975152695198	-0.41136888074230
H	1.45259398243347	1.77199238286870	3.50408789159340
H	1.15754476218130	-0.50259297750869	2.75834319953394
H	2.03759270633296	1.14982018567200	1.24848012440182
C	-3.36726117403071	-0.22562312472500	-1.49653697574079
C	-0.67794033133448	1.31340565121851	1.76027488088064
C	-1.12233130627427	0.45421406735109	0.55824044917706
H	-2.69287783759778	-0.07253750973938	-2.34207686936411
H	-4.37988192884457	0.01254078873916	-1.82910148557806
H	-3.34210353303737	-1.28572574203085	-1.23397609749772
H	-1.07126877381135	-0.60977902889734	0.81373612591958
H	-0.43176811103293	0.59143843888858	-0.28165332706183
H	-0.72728440507524	2.37703241054729	1.50603707092794
H	-1.36633724213123	1.17632028223510	2.60084296211997

H_3Ge-SiH_2-$(GeH_2)_2$-SiH_3 E = -6812.143155082050 au
ZPE = 0.09662622 au
G_{corr} = 0.05592044 au

Si	-3.33788637446980	0.86964835792590	-0.26196868064350
Si	1.88069435464603	1.06073463518002	2.95399024804818
H	-4.26855536877070	0.65160778067655	0.87470550128896
H	-3.37141322868484	2.31198265054964	-0.61462715831660
H	2.34084797000214	1.88519168717236	4.09807855340711
H	1.92915803490076	-0.36981937872455	3.34096985830713
H	2.79476387495458	1.27635763310722	1.80659011265575
Ge	-4.04204845034171	-0.43137656951220	-2.14119545188217
Ge	-0.34883381253754	1.63782262107965	2.31285768973059
Ge	-1.12251217373632	0.29098539578502	0.42509327954625
H	-3.08977395948878	-0.20378604492125	-3.33045950230094
H	-5.47012552046241	-0.06077840184048	-2.58641430599371
H	-4.01886434391397	-1.93099280450522	-1.78966762482242
H	-1.07268752631623	-1.20164760519603	0.81678509448755
H	-0.14716494347879	0.47885974436981	-0.75711336539886
H	-0.42169221203105	3.12814838052581	1.91762305188903
H	-1.31785038027132	1.43302186832772	3.49668270999764

H_3Sn-SiH_2-$(SnH_2)_2$-SiH_3 E = -1226.269375196609 au
ZPE = 0.08865119 au
G_{corr} = 0.04508062 au

Si	-3.51695972013155	0.86111670718297	-0.36120606461954

Si	2.20601008482650	1.12546903235004	3.20375509635501
H	-4.44631581472606	0.64302510501773	0.77712728776091
H	-3.54399414769303	2.30846243849153	-0.69520774619614
H	2.67163938577607	1.94925973438464	4.34592731987836
H	2.25132435520337	-0.30479553659251	3.59172009631517
H	3.11749471399294	1.33737743141171	2.05384140655230
Sn	-4.31652319928702	-0.50204031015411	-2.39910090260332
Sn	-0.20146959744002	1.75593633823838	2.52172209315930
Sn	-1.12903550026575	0.22041925109260	0.37605575831247
H	-3.27623198088423	-0.23243769824680	-3.73400085296882
H	-5.91058980442324	-0.07881787257835	-2.86725063920433
H	-4.29097507664340	-2.17721834065526	-2.03840730873807
H	-1.09493007525063	-1.44341328429280	0.80456396043478
H	-0.05893266944422	0.40016057961884	-0.95675370373545
H	-0.27751813751652	3.41581092331495	2.08617971061469
H	-1.26693687609315	1.54764545141640	3.85296449868269

H_2N-SiH_2-$(NH)_2$-SiH_3 E = -747.558941351050 au
ZPE = 0.10093208 au
G_{corr} = 0.06794497 au

Si	-2.76088733550928	0.89758706921807	0.29749366978856
Si	0.86684627884632	1.32169636265721	1.85417004076406
H	-4.05832658551840	0.79498171221300	1.02037067423700
H	-2.37590736524559	2.32726076337734	0.23645304248469
H	1.31245225782982	1.92475119285274	3.13430409786862
H	1.80731710197440	0.23535125507918	1.46811127177005
H	0.89454038283548	2.32987778020480	0.76805352622389
N	-3.03215924488850	0.09429586778681	-1.19101509232535
H	-3.96406987521020	-0.11047373789991	-1.51033531241846
H	-2.36438402141175	0.10520044780977	-1.94463241031508
N	-1.41847162232104	0.12154533042249	1.05998033417224
N	-0.73465398182765	0.75206701773362	2.12164170500334
H	-1.29104446574905	-0.88099065329374	1.02048511938332
H	-1.15920566380449	0.65543651183855	3.03177440336306

H_2P-SiH_2-$(PH)_2$-SiH_3E = -1606.238546837742 au
ZPE = 0.07996498 au
G_{corr} = 0.04343929 au

Si	-2.90869109216733	0.36295178523508	0.33313594998505
Si	1.15589953644738	0.99853877639210	2.44505355602871
H	-3.33940074304701	-0.15378482291545	1.19381794986266
H	-3.19576963579752	1.41202210889102	0.38878998136077
H	1.53474656992268	1.61049895349886	3.26513554359743
H	1.35614328689365	-0.04688085235297	2.68136498699334
H	1.68984819764710	1.26247714457517	1.53250498517679
P	-3.57591223142009	-0.48072054731005	-1.19454583109539
H	-4.84644732677585	0.15602713925297	-1.21203259014082

H	-3.02880645143810	0.41745255697838	-2.14902736801420
P	-1.06454262425692	0.12017613962917	0.44289226007197
P	-0.68492254013904	1.23684189602342	2.32905050902961
H	-0.69351759877192	1.11464087492868	-0.49951979910939
H	-0.67658148709696	2.55834576717358	1.81023493625344

H_2As-SiH_2-$(AsH)_2$-SiH_3 E = -7286.929708423387 au
ZPE = 0.07506256 au
G_{corr} = 0.03552165 au

Si	-3.28302277668635	0.32057453351437	0.33881798382305
Si	1.61432270549402	1.16161249483634	2.57941910945723
H	-4.11913123447340	-0.32202323466314	1.38185723671026
H	-3.45712345466632	1.79060798652180	0.43784175453333
H	2.14058330318763	2.21999531625179	3.47551195432354
H	2.08446330201673	-0.15951467699041	3.05697272390009
H	2.12058592277646	1.39012582126641	1.20517274222250
As	-3.95430561550257	-0.55932825719396	-1.77661849626611
H	-5.17848206032859	0.35139794934883	-1.84317557316965
H	-3.09083499705161	0.44783898863698	-2.53200613776357
As	-1.03465711766006	-0.34398127158601	0.74031373814282
As	-0.76217871088458	1.17119004850310	2.69360894282853
H	-0.48485467643519	0.61793733131669	-0.31034732478596
H	-0.87331872978608	2.48215389023714	1.91948641604388

H_2Sb-SiH_2-$(SbH)_2$-SiH_3 E = -1302.319435703557 au
ZPE = 0.06960309 au
G_{corr} = 0.02755159 au

Si	-3.44656367428924	0.21741557671104	0.32825927946463
Si	1.84977714785034	1.22473572516771	2.73942062109733
H	-4.30021651713339	-0.41986407182104	1.36418846663214
H	-3.58905331693575	1.68858980299967	0.46199185887491
H	2.45269803107755	2.34325071309159	3.50544245589647
H	2.30597680618940	-0.05690303562652	3.33022552958228
H	2.31480894112236	1.29617912421988	1.33374092948575
Sb	-4.23503624999858	-0.64212272750873	-1.99690304192443
H	-5.50687529643605	0.50757971902047	-2.06281154270317
H	-3.16078564924374	0.43776548117505	-2.78435548729055
Sb	-1.01306688116097	-0.56049881268898	0.76252541171094
Sb	-0.73485992121936	1.31916857894664	2.91946677052055
H	-0.41451524709009	0.53170898215303	-0.41537487872727
H	-0.80024231273237	2.68158186416011	1.88103869738038

H_2Bi-SiH_2-$(BiH)_2$-SiH_3 E = -1225.401822677802 au
ZPE = 0.06678795 au
G_{corr} = 0.02236863 au

Si	-3.46556373001018	0.06098148491460	0.29755575068307

Si	1.94028986627699	1.27389967672727	2.81682823305187
H	-4.28599972908965	-0.71331152640823	1.26910065106089
H	-3.67938392876695	1.50056987219915	0.59104511221733
H	2.54042042864699	2.38864976509842	3.59214005517817
H	2.41992227163669	-0.00703279917966	3.39471703710443
H	2.40871122216954	1.36148141927213	1.41297435343692
Bi	-4.30333404304210	-0.57298163861784	-2.18164141708259
H	-5.72516058884213	0.51661910687525	-2.01449439922706
H	-3.32275603921905	0.77832064543235	-2.84580736493729
Bi	-0.90960877673730	-0.65339284004272	0.68982887695126
Bi	-0.73607263654814	1.32886021566067	2.98530974061076
H	-0.37467650247451	0.57600027146154	-0.50447584808718
H	-0.78474195400009	2.72992326660702	1.86377428903937

HO-SiH$_2$-(O)$_2$-SiH$_3$ E = -807.128831589339 au
ZPE = 0.06457077 au
G_{corr} = 0.03246592 au

O	-2.82639632839963	0.39954317658287	-0.02794632061962
O	-0.13965650348429	1.47412975676071	2.55990175111208
O	-0.59271757256969	0.71520755552296	1.37071119911445
Si	-2.20002590290810	1.21382106649032	1.24788661050566
Si	1.35474880361696	2.09745155910609	2.09733786031946
H	-2.95545731686020	0.80193853665288	2.43902675137412
H	-2.21199942146585	2.67733291426487	1.04343643308561
H	2.28982027380844	1.00683151044745	1.75521774647758
H	1.79046768633607	2.82336319177886	3.30970669962892
H	1.20102963918570	3.01618546331948	0.94885978021041
H	-2.54463215725937	0.62766984907344	-0.91727820120873

HS-SiH$_2$-(S)$_2$-SiH$_3$ E = -1774.985738483329 au
ZPE = 0.05597682 au
G_{corr} = 0.02075122 au

S	-3.40162421845203	0.39635624575033	-0.40813402670391
S	0.14309048718140	1.07165239821788	3.24670492697631
S	-0.61937134431986	-0.05949525623434	1.63727710793039
Si	-2.35944138304515	1.16407769445003	1.29989096983685
Si	1.53887809742457	2.26982873389108	2.12131039067334
H	-3.30510099082165	1.05207388640429	2.42749617548773
H	-1.92815254760968	2.56376573866193	1.11200406646783
H	2.54225642224441	1.40395282217152	1.46652482844936
H	2.18683570771652	3.15752833047352	3.11495787362397
H	0.82518254170924	3.06600228363945	1.09999586644664
H	-2.45737157202772	0.76773170257419	-1.29116786918857

HSe-SiH$_2$-(Se)$_2$-SiH$_3$ E = -7782.156505802613 au
ZPE = 0.05301251 au

G_{corr} = 0.01477014 au

Se	-3.53939967050149	0.40108043884658	-0.54279486590187
Se	0.25523999311899	1.04595219895913	3.47394355344574
Se	-0.64145969658996	-0.25907930005080	1.68496383590416
Si	-2.42574028073814	1.13703707923144	1.32087022595024
Si	1.61080895784260	2.33123399224498	2.13202528852765
H	-3.40483531938938	1.05286229516530	2.42329650021870
H	-1.93575313371277	2.52095665486526	1.16322841675952
H	2.60144171325582	1.48075143761825	1.43926156426812
H	2.29255106155853	3.28314641422948	3.04056696263082
H	0.79760907671918	3.06321463581059	1.13761255214789
H	-2.44528150156330	0.79631873307968	-1.44611372395107

HTe-SiH$_2$-(Te)$_2$-SiH$_3$ E = -1384.123571626460 au
ZPE = 0.05051128 au
G_{corr} = 0.00987461 au

Te	-3.78701467219435	0.43674545594261	-0.73975377796683
Te	0.41692936058334	1.00534451085567	3.76976435867219
Te	-0.68219215761378	-0.50417824618665	1.73693565680629
Si	-2.55115881205088	1.12332292362489	1.34145464213744
Si	1.75235405109775	2.40726338789095	2.16028993344542
H	-3.55889281000019	1.07422423670744	2.42379608883762
H	-1.99949098604113	2.49058207593503	1.23065938274167
H	2.72398064196641	1.56461801541102	1.43055566487282
H	2.46680347454622	3.42815531817626	2.96394903931806
H	0.84346612935556	3.06641171134354	1.19740150016804
H	-2.45960301964888	0.76098519029911	-1.68819217903283

HPo-SiH$_2$-(Po)$_2$-SiH$_3$ E = -1293.279336151368 au
ZPE = 0.04920366 au
G_{corr} = 0.00653770 au

Po	-3.87346095598220	0.44482235956374	-0.82264683127002
Po	0.48030906715871	0.99639666950286	3.90773943176547
Po	-0.69497873293886	-0.62431754025205	1.76758425216829
Si	-2.58746550436587	1.11130093423299	1.35528867302277
Si	1.79253282263749	2.43506962437456	2.16598602420824
H	-3.60880409224508	1.08839088072411	2.42850022361323
H	-2.00976925739715	2.46984417938569	1.25553398212611
H	2.76668251172156	1.59639677495387	1.43378731414096
H	2.50688869741835	3.50030904117889	2.91197968618235
H	0.84455427220949	3.04437197781301	1.20730256899126
H	-2.45130762821636	0.79088967852221	-1.78419501494877

H$_3$C-CH$_2$-GeH$_2$-CH$_2$-CH$_3$ E = -2234.961507893052 au
ZPE = 0.14569946 au
G_{corr} = 0.11284893 au

Ge	-0.87683620373829	0.33775592354172	0.35720510160058
H	-0.64298607529007	-1.19118844542633	0.42834362425629
H	-0.03790602293169	0.88229655346299	-0.82478311371622
C	-3.31762291273588	0.06082358107196	-1.26662398536015
C	-2.79075052547615	0.69348767330098	0.02299806476453
C	-0.26307980763288	1.17487343937608	2.03774474505105
C	1.21653351467412	0.93046588398976	2.34100012990796
H	-2.78000867002210	0.43194751215220	-2.14251287266525
H	-4.37816690153441	0.28011723929068	-1.41596309988221
H	-3.20696891682101	-1.02609357216038	-1.25260268108521
H	-2.92672221119100	1.77797073931753	0.00184650380618
H	-3.35097448402885	0.32661131982092	0.88705084432461
H	-0.88949491047971	0.79027809701397	2.84691127633171
H	-0.46872459820518	2.24628547918277	1.96752586806449
H	1.43431265867941	-0.13589594209500	2.43378079476123
H	1.51808022608904	1.40908705896176	3.27665724252427
H	1.85717721064471	1.32622775919839	1.54965096731609

H_3Si-SiH_2-GeH_2-SiH_2-SiH_3 E = -3239.024434617420 au
ZPE = 0.09960161 au
G_{corr} = 0.06073397 au

Ge	-0.91473274603924	0.32488306046309	0.40548365146911
H	-0.69700781536327	-1.20128261285031	0.49284561216821
H	-0.06360614983200	0.82844333385957	-0.78076740472373
Si	-3.92037603115185	-0.20172586622999	-2.04226720474222
Si	-3.22488385012844	0.77858334325897	-0.02891849945577
Si	-0.14804042045700	1.35758388099879	2.42464476294409
Si	2.13468490855947	0.95415142152657	2.77926729000774
H	-3.10847258822098	0.33499424043245	-3.16219715941454
H	-5.35255268505701	0.07477674372480	-2.31706053171383
H	-3.72309933621374	-1.67072249905232	-1.97633058326187
H	-3.41641342733880	2.25091856810945	-0.08614485703877
H	-4.03421478601022	0.25579042394268	1.10214704586992
H	-0.94392578622175	0.82786069145206	3.56208204219486
H	-0.39734886495243	2.81961898580487	2.33439084792378
H	2.37762479140669	-0.50724860545760	2.86013735258246
H	2.60948184324580	1.58604518569429	4.03568170700254
H	2.91874431377487	1.50238000432261	1.64523533818795

H_3Sn-SnH_2-GeH_2-SnH_2-SnH_3 E = -2937.433700875438 au
ZPE = 0.08255481 au
G_{corr} = 0.03613724 au

Ge	-0.93138992953886	0.25862680942539	0.44655840375669
H	-0.73738835859647	-1.26661428243827	0.59163838395684
H	-0.07178802500048	0.70274379137376	-0.75763808982582
Sn	-4.29158703327803	-0.31302040700926	-2.50162773873465
Sn	-3.45307377644529	0.76989701336247	-0.06397022411246

Sn	-0.05610876072911	1.45762510710128	2.60977900613761
Sn	2.66531699269535	0.99863228817816	3.05204393256891
H	-3.36422992572486	0.33722635095741	-3.78769204476246
H	-5.94444237845333	0.02298468903664	-2.80853596485707
H	-4.08154391203715	-2.01381471006497	-2.50176689388218
H	-3.67478846695782	2.47306739512962	-0.07759118310739
H	-4.41311588259713	0.15210049299061	1.21898553719451
H	-0.97299582122164	0.91114151574003	3.95519710491287
H	-0.34774570591846	3.14338252722335	2.45853386262587
H	2.96803751045798	-0.68084086689538	3.20805287239047
H	3.21681402676554	1.77794078741454	4.47596583552691
H	3.58589081657984	1.58397179847461	1.73029661021126

H_2N-NH-GeH_2-NH-NH_2 E = -2299.031338036310 au
ZPE = 0.10172380 au
G_{corr} = 0.06952203 au

Ge	-0.95138870164719	0.33735966039017	0.40222989738628
H	-0.82826668716133	-1.10779117132090	0.93083689333375
H	-0.15309239595034	0.51476244089645	-0.90704071192648
N	-0.46430145632013	1.43994887835022	1.80877979832491
H	-0.57045691071847	2.43431131970594	1.63901761119245
N	-2.66873495153022	0.92828182687616	0.03840944035382
H	-3.30336479457558	0.94218639008440	0.82940192435432
N	-3.24233235853190	0.39049958116827	-1.15273525099125
H	-3.81859264498812	-0.42388584018289	-0.96312980210708
H	-3.82088574922636	1.09726085511539	-1.59150950671937
N	0.76989934355771	1.09062219474608	2.43515727032158
H	1.55094886604122	1.61780054259777	2.05650292758057
H	0.70477448105082	1.28306130157288	3.42785379889641

H_2P-PH-GeH_2-PH-PH_2 E = -3444.039766350541 au
ZPE = 0.07139720 au
G_{corr} = 0.03410528 au

Ge	-1.18590141040045	0.01467251720618	0.80588636411352
H	-0.95034675514236	-1.49613069677719	0.98248475388191
H	-0.35046130419190	0.50655572450554	-0.40021848382610
P	-0.58718013667403	1.09020343319769	2.79520096093017
H	-0.87857844815922	2.39176733987187	2.30913093803501
P	-3.47769624977531	0.36861553198285	0.49858935347110
H	-3.35383199945150	1.77460964878448	0.34697445917889
P	-3.53632020525888	-0.24104753556685	-1.65177046801506
H	-4.85072951868386	0.23560362581751	-1.89253863533416
H	-2.91146735706898	0.89145502492315	-2.23256071623911
P	1.62086778335084	1.04059614724198	2.44733047523040
H	1.72101609385891	2.03595929310954	1.44252276554096
H	1.94483554759681	1.93155792570322	3.50274252303244

H_2As-AsH-GeH_2-AsH-AsH_2 E = -11018.297041874031 au
ZPE = 0.06433325 au
G_{corr} = 0.02286499 au

Ge	-1.23056968783766	-0.06139152297332	0.88423130911657
H	-0.97479614183607	-1.57558108232927	1.03129141336809
H	-0.40127431583088	0.44837560887697	-0.31807916223921
As	-0.57985180693033	1.02604135243152	2.98080031950024
H	-0.89803895029077	2.41399372834828	2.43078750423204
As	-3.63419330142174	0.27567687448529	0.55728445147079
H	-3.45944354605754	1.78130278550448	0.37607631036011
As	-3.57137026708265	-0.30309960453707	-1.86045798654579
H	-4.94622323036823	0.30738383467819	-2.12430630928741
H	-2.85781599677046	0.96737157805192	-2.31031102993264
As	1.84181623340009	1.05346812782297	2.41827458536818
H	1.74748761765224	2.15330455868216	1.36632882712961
H	2.16847943337409	2.05757174095783	3.52185405745936

H_2Sb-SbH-GeH_2-SbH-SbH_2 E = -3038.828592758589 au
ZPE = 0.05690407 au
G_{corr} = 0.01199476 au

Ge	-1.34531606686089	-0.21953120252490	1.08137956559516
H	-1.13857334371665	-1.73487428359293	1.31194107266164
H	-0.47452890228897	0.16729386560944	-0.13911191885197
Sb	-0.57905335961100	1.05911196397788	3.26966567466860
H	-0.91743428680559	2.56954754081592	2.53378768138001
Sb	-3.91876730453917	0.19710060254928	0.61564255117107
H	-3.63778574440648	1.87692615517999	0.42395843297573
Sb	-3.57084877640386	-0.36417785798929	-2.18320816110803
H	-5.07922812185517	0.35950929410772	-2.56949991278349
H	-2.74799330325060	1.11000739006899	-2.47094938250678
Sb	2.16196607725532	1.04355607620812	2.39831540266520
H	1.87375625411620	2.19949632593118	1.16780590573626
H	2.57801291836692	2.28045210965855	3.51404737839654

H_2Bi-BiH-GeH_2-BiH-BiH_2 E = -2936.277003849903 au
ZPE = 0.05303859 au
G_{corr} = 0.00486808 au

Ge	-1.43533512019338	-0.25390748621704	1.20479148266582
H	-1.27093155854518	-1.77408912247334	1.47008796970086
H	-0.53659762251023	0.05432000973582	-0.02132153006220
Bi	-0.55284648708830	1.08630508300818	3.41992130680211
H	-0.90689878544046	2.65030027003892	2.61348432547682
Bi	-4.06671133826612	0.21532615980105	0.62290645906896
H	-3.70622672168769	1.95930420711967	0.39870075648988
Bi	-3.47826546319380	-0.40362852094265	-2.29363887581751
H	-5.08113378728335	0.24529574401831	-2.79976552065535

H	-2.73421512524360	1.21005730616116	-2.53968425847784
Bi	2.26888627833323	1.00884813721122	2.29352359673350
H	1.88504366348292	2.29740125798280	1.10589249906224
H	2.81943810763605	2.24888493455581	3.47887607901268

HO-O-GeH$_2$-O-OH E = -2378.372241955891 au
ZPE = 0.04963503 au
G_{corr} = 0.01746775 au

O	-3.35287843915707	-0.62407627050244	2.85754732595601
H	-3.87195377759364	-1.37602472649693	2.53701068290130
O	1.63239230178990	-0.69769262607080	2.01362070568702
H	2.06805237441470	0.13822099657663	1.79217460648053
O	-2.37910367987646	-0.49437976351372	1.76668276046882
O	0.35801301561155	-0.52378410140660	1.30573652898493
Ge	-0.82018536542560	-0.69558988499707	2.67727508883691
H	-0.74323966980312	-2.10683406905771	3.27099550811079
H	-0.63152759996023	0.45531127546865	3.67223239257363

HS-S-GeH$_2$-S-SH E = -3668.985050950709 au
ZPE = 0.03803248 au
G_{corr} = 0.00221907 au

S	-3.73880122591756	-1.66895284586111	2.60300326210096
H	-3.59548174312704	-1.67376488125167	1.26367571671181
S	1.77033528285830	-1.44306609656776	1.55320704521575
H	2.49355783086916	-0.30574636226032	1.53662181187078
S	-3.02527679308663	0.25499060996150	2.97099736510323
S	-0.08657011630628	-0.62777620387390	1.07405724193099
Ge	-0.82547152275411	-0.17247324019690	3.15576047243951
H	-0.57550886523575	-1.39739316618832	4.04498225017463
H	-0.15721368730005	1.10933301623850	3.69097043445228

HSe-Se-GeH$_2$-Se-SeH E = -11678.558894708693 au
ZPE = 0.03376668 au
G_{corr} = -0.00655570 au

Se	-4.33398973612277	-0.52889246713469	3.27947797678416
H	-4.57476451721728	-1.93163198088088	2.88588490519095
Se	2.68623471005169	-0.88849515006675	2.06312018716115
H	2.88219159053578	0.57495401147809	2.03831896193420
Se	-2.87521475592696	0.00028562664881	1.49187921179239
Se	0.71196936970845	-0.91193641393792	0.75793771710786
Ge	-0.84039965744375	-0.66410289181763	2.56044910363316
H	-1.07840873051878	-1.99386930924485	3.29605705207366
H	-0.31804911306634	0.41883940495583	3.52015048432240

HTe-Te-GeH$_2$-Te-TeH E = -3147.874087814406 au

ZPE = 0.03060821 au
G_{corr} = -0.01201469 au

Te	-4.21402550704462	0.44452374372964	2.51398806623922
H	-4.71367304910604	-0.33755338075655	3.89954019536525
Te	2.36595384432283	0.84464600924415	2.84484273488987
H	3.37831040254398	-0.20015211099318	2.02891458921936
Te	-3.17250580017728	-1.79551613666780	1.33294081260193
Te	0.25287361617212	0.57244135642024	1.12403990924295
Ge	-0.79153903038135	-1.47649243652457	2.30994180601380
H	0.01559792506108	-2.75881743800123	2.01392424740545
H	-0.86142324139066	-1.21792877645068	3.82514323902204

HPo-Po-GeH$_2$-Po-PoH E = -3026.757575939604 au
ZPE = 0.02887230 au
G_{corr} = -0.01636589 au

Po	-4.28005045622083	0.54433836937113	2.53763213143323
H	-4.84459803218145	-0.26567194834216	3.99046318205962
Po	2.46239503940327	0.96257007625416	2.88301584035310
H	3.57384215674198	-0.11974131894658	2.05888218017985
Po	-3.26768044799201	-1.88342903055820	1.29041966240805
Po	0.25541284837204	0.54974720110137	1.02787561383828
Ge	-0.79502174079818	-1.56256471663254	2.28915305489836
H	0.01228231500819	-2.85119279045461	2.00999798181881
H	-0.85701252233295	-1.29890501179256	3.80583595301059

H$_3$C-GeH$_2$-(CH$_2$)$_2$-GeH$_3$ E = -4272.524795092841 au
ZPE = 0.13016995 au
G_{corr} = 0.09451018 au

Ge	-2.93760183937772	0.86566908475212	-0.04844040010803
Ge	1.15612372014870	0.92150648639506	2.39871277402424
H	-3.92933946419255	0.63767595011897	1.11602158259805
H	-3.01253277905677	2.35722581606830	-0.44995863622203
H	1.55157096554047	1.80684371894075	3.59868223229450
H	1.24650155516459	-0.55916763832095	2.82197662088223
H	2.16040727033854	1.16017751097864	1.25234363224153
C	-3.42530549856989	-0.27004052403067	-1.58058687213007
C	-0.67551845704408	1.32589720608440	1.77561018006207
C	-1.10581934849282	0.46618595371579	0.58377948082126
H	-2.73239934493532	-0.10703130329062	-2.40664339774221
H	-4.43396192061279	-0.03962808330586	-1.92356067398201
H	-3.38731179043177	-1.32233350479187	-1.29726003894459
H	-1.06923554094872	-0.59691053068074	0.83709939689823
H	-0.42762336667497	0.60627750395795	-0.26250201583065
H	-0.71095403298832	2.38865898377753	1.52359607082426
H	-1.35094418786652	1.18495331963116	2.62306007431321

H_3Si-GeH_2-$(SiH_2)_2$-GeH_3 E = -5025.580950807469 au
ZPE = 0.09802498 au
G_{corr} = 0.05815658 au

Ge	-3.31716518167985	0.87632651815935	-0.26355289764595
Ge	1.83149721380332	1.04454067782559	2.92983965155220
H	-4.28363012677942	0.66986075957973	0.92218916182464
H	-3.37209128773331	2.37344721425121	-0.63539800830250
H	2.29994085334887	1.89158226268969	4.12871787739589
H	1.90377652707542	-0.44107793331971	3.33079971703600
H	2.81384734006359	1.27234354896278	1.76527435987100
Si	-4.01903009405006	-0.45115348062766	-2.12535091588270
Si	-0.39401327644986	1.61415107464958	2.25933990766358
Si	-1.09450041283153	0.32445452049683	0.43352832290952
H	-3.09984930092955	-0.22771443831946	-3.26734061826952
H	-5.39889279848916	-0.10430229628921	-2.54413756352630
H	-3.97661975629428	-1.88613163135625	-1.75377891972110
H	-1.04570199208763	-1.11563234033672	0.79690280175315
H	-0.16468596081754	0.53442385227066	-0.70648072994818
H	-0.44458040212160	3.05279803884702	1.89257132681893
H	-1.32224540402731	1.39804360251652	3.39880653647135

H_3Sn-GeH_2-$(SnH_2)_2$-GeH_3 E = -4799.391618321759 au
ZPE = 0.08556184 au
G_{corr} = 0.03963159 au

Ge	-3.56298853071823	0.82561392268746	-0.34831817954677
Ge	2.26087461135348	1.16749106651631	3.19542189581981
H	-4.56302343370654	0.57514039473216	0.80062918883105
H	-3.58574577504908	2.34159564825251	-0.63973768355392
H	2.76570088014203	2.06418185719227	4.34147122445791
H	2.34845487516500	-0.30113754027002	3.64950167260210
H	3.18948982586402	1.35467834135442	1.98188353733710
Sn	-4.32654163702943	-0.48174070145360	-2.48713098708103
Sn	-0.20792346566135	1.75376869134139	2.53560656188387
Sn	-1.15566049017680	0.15189658022608	0.44845746985240
H	-3.24022380302856	-0.17048067486761	-3.77471961572628
H	-5.90263544307221	-0.02271377049636	-2.98007917566824
H	-4.32233273604119	-2.16622028104153	-2.17455366149260
H	-1.16925751406403	-1.49556899529807	0.93593217731557
H	-0.08207582145006	0.25944742521444	-0.88915363375062
H	-0.30005495612700	3.40247584210793	2.06355841327566
H	-1.23000064639997	1.56753214380216	3.90316080544399

H_2N-GeH_2-$(NH)_2$-GeH_3 E = -4320.611600739925 au
ZPE = 0.09671952 au
G_{corr} = 0.06181576 au

Ge	-2.88517061548791	0.91745637170269	0.24943291097238
Ge	1.04131575634398	1.32359021520244	2.00071381726225

H	-4.09718031085590	0.64823184335387	1.17523675335400
H	-2.68975261362452	2.43134030527189	0.03611792968387
H	1.42344194390932	1.90613696236584	3.37574023221238
H	1.86980671156584	0.04695291539300	1.71650157432226
H	1.27082615041139	2.33967880433783	0.87288629035127
N	-3.15679031844546	-0.10742688088614	-1.25995886117706
H	-4.12257071920020	-0.22202277426288	-1.53390738874541
H	-2.60800953427154	0.15267983031773	-2.06792455377778
N	-1.26495890602884	0.30124069507254	0.89777569812733
N	-0.78045277701768	0.96820810047430	2.05912981942338
H	-1.14879211870608	-0.70654990753558	0.92103907035988
H	-1.12966678859232	0.56907043919241	2.92407177763118

H_2P-GeH_2-$(PH)_2$-GeH_3 E = -5179.346510236993 au
ZPE = 0.07655498 au
G_{corr} = 0.03793929 au

Ge	-3.27975132427278	0.34194051261331	0.35602043170345
Ge	1.58551732788027	1.12798722802433	2.53804222196696
H	-4.11328846478368	-0.41492528347947	1.40756888997774
H	-3.55429913578972	1.85570041403977	0.50031060795559
H	2.07147067633061	2.21113303648889	3.51896104021561
H	2.05543592692678	-0.25377239788821	3.02111245944408
H	2.16949064061317	1.40459771261485	1.13903625605874
P	-3.82974741940120	-0.49897158857026	-1.76388015241533
H	-5.06298595026438	0.20279722863981	-1.82925068940577
H	-3.12202439377365	0.52171962594287	-2.45224590043112
P	-1.02903192246809	-0.16964626419275	0.73773618655946
P	-0.75341363754867	1.12893262863092	2.53344954620914
H	-0.53742747842477	0.70421018599365	-0.26797392462606
H	-0.87789898502382	2.40688388114224	1.92796809678746

H_2As-GeH_2-$(AsH)_2$-GeH_3 E = -10860.042049158565 au
ZPE = 0.07163524 au
G_{corr} = 0.03016602 au

Ge	-3.34189344363455	0.31306200727755	0.33748925350223
Ge	1.68614862630861	1.18077718886576	2.60053759913945
H	-4.23290170036634	-0.35039350527574	1.40832580552341
H	-3.50616284245948	1.84447637561865	0.44774555569022
H	2.21021993755293	2.31320438900065	3.50427363774965
H	2.21819714531676	-0.16654823282744	3.12004349474903
H	2.19012185219156	1.40224420830031	1.16212257855584
As	-4.01507240513398	-0.56117282425175	-1.85717284042259
H	-5.21049154213025	0.38527626043104	-1.94377913631288
H	-3.11310147468848	0.42908306524554	-2.58967204814883
As	-1.03299693930074	-0.38013254112132	0.77287652467455
As	-0.75848015305775	1.13298121245263	2.72629972919176
H	-0.47566491021208	0.58669425859403	-0.26970513502576

H	-0.89587629038612	2.43903505769000	1.94747005113387

H_2Sb-GeH_2-(SbH)$_2$-GeH_3 E = -4875.438558732128 au
ZPE = 0.06636625 au
G_{corr} = 0.02224507 au

Ge	-3.49402725177497	0.20408213090903	0.31678253571316
Ge	1.90965617259488	1.23561767594444	2.76938233109718
H	-4.39013020289276	-0.46303640930942	1.38591802153263
H	-3.65026331975630	1.73276896002048	0.47226981768218
H	2.52208352107454	2.41997010943532	3.54308294770590
H	2.40642052492269	-0.07438704386133	3.41124701059707
H	2.40313032786157	1.28560492517433	1.31108225571416
Sb	-4.28591765720356	-0.64697327780966	-2.06963403556647
H	-5.55200804150719	0.50904359762699	-2.13293029864960
H	-3.20535365684829	0.43758658859379	-2.84186305374615
Sb	-1.00478075604619	-0.57185514539145	0.77683701786680
Sb	-0.73017831107983	1.30808532695622	2.93250373350915
H	-0.40706414163019	0.52754621168489	-0.39469625265928
H	-0.79952134771428	2.66453327002628	1.88687303920323

H_2Bi-GeH_2-(BiH)$_2$-GeH_3 E = -4798.523745497505 au
ZPE = 0.06359604 au
G_{corr} = 0.01662456 au

Ge	-3.53527942134275	0.00320926172095	0.31360247385419
Ge	1.99433887765011	1.30965635333196	2.81593580211577
H	-4.42688483327561	-0.85007389820827	1.25194171293411
H	-3.78078379466904	1.48081681497413	0.69679025920582
H	2.60594063817888	2.52659163682947	3.54141108625618
H	2.52558649084774	0.03243768841369	3.50081792101668
H	2.48712821985632	1.31196394732552	1.35591142267323
Bi	-4.28865052869126	-0.49367078167308	-2.27560872491109
H	-5.72126711026883	0.58098913461138	-2.10643999135578
H	-3.29094913984204	0.89632718681720	-2.82380127209361
Bi	-0.93729203641648	-0.72408657883040	0.77076104427284
Bi	-0.73228375482774	1.34406877738238	2.98424340947608
H	-0.37886302686200	0.45524953595834	-0.46286080006359
H	-0.79869472033719	2.69510784134667	1.80415072661914

HO-GeH_2-(O)$_2$-GeH_3 E = -4380.164497399391 au
ZPE = 0.05974468 au
G_{corr} = 0.02517549 au

O	-2.92900499271985	0.34893205639441	-0.14511066348187
O	-0.14076286322669	1.45497230604018	2.60302519597241
O	-0.57461985011534	0.67717572191980	1.42021061410491
Ge	-2.30484665624552	1.22145332320385	1.27291446591180
Ge	1.47149499143420	2.13426439675245	2.10401608123290

H	-3.11230131885122	0.72556569580892	2.47194166240501
H	-2.28619532350029	2.74410244206012	1.05745074502481
H	2.40336518511928	0.96376609705781	1.76545281460714
H	1.91102949665406	2.90491756754773	3.35817043411064
H	1.25147979659434	3.05519903502314	0.89326752737650
H	-2.52445726514291	0.62312593819149	-0.97447856726434

HS-GeH$_2$-(S)$_2$-GeH$_3$ E = -5348.076959721025 au
ZPE = 0.05204695 au
G_{corr} = 0.01464071 au

S	-3.42773085568410	0.37772094713151	-0.52667974941308
S	0.17696035969915	1.02569922214399	3.29597821810370
S	-0.63020297321447	-0.11439197006044	1.73160642084240
Ge	-2.44263650689349	1.16090602465903	1.32254100249792
Ge	1.61542480530388	2.29614955436737	2.11300459191611
H	-3.47055482968851	1.00838562171109	2.45272308020599
H	-1.97352283696300	2.61542802830148	1.15814965909648
H	2.64756415006881	1.38139198752845	1.43766450386450
H	2.28227882267456	3.23777587984700	3.13084141808976
H	0.82729704240760	3.08677344828143	1.05693741142457
H	-2.43969597771037	0.77763583608897	-1.34590624662842

HSe-GeH$_2$-(Se)$_2$-GeH$_3$ E = -11355.257031366964 au
ZPE = 0.04920538 au
G_{corr} = 0.00898131 au

Se	-3.60952629090931	0.36143168820312	-0.62998859779734
Se	0.27783147118642	0.99739282390433	3.49760980497530
Se	-0.63890898711482	-0.31096612499418	1.73590971263199
Ge	-2.49421312437257	1.12963352159628	1.32290778500585
Ge	1.69304823317605	2.36063374978440	2.14321649903791
H	-3.51949778978027	1.03064316134212	2.46309467616727
H	-1.97200019896209	2.56537203313683	1.15322611862136
H	2.73691552563275	1.47579224249224	1.44669171510099
H	2.36713564976263	3.35839954893652	3.10196990839065
H	0.83420575122595	3.09653299888305	1.10342228199685
H	-2.50980903984464	0.78860893671516	-1.51119959413094

HTe-GeH$_2$-(Te)$_2$-GeH$_3$ E = -4957.233637884731 au
ZPE = 0.04699287 au
G_{corr} = 0.00446339 au

Te	-3.82004677534280	0.41379019636240	-0.82436557690559
Te	0.42934525647780	0.97441212977343	3.80234377059359
Te	-0.67752618776515	-0.55724263642370	1.80375884186981
Ge	-2.60660374688047	1.10489360421775	1.35182822484838
Ge	1.80779200913280	2.42356807185676	2.15847984566281
H	-3.66770020272411	1.04305460935893	2.46594322544058

H	-2.02668945114644	2.52681307581232	1.24750530225766
H	2.82822269144928	1.53972752934917	1.42505782068057
H	2.52973868601139	3.50429922026163	2.98513212606772
H	0.85306713715652	3.07615115953898	1.14635018260099
H	-2.48441821636876	0.80400761989218	-1.73517345311665

HPo-GeH$_2$-(Po)$_2$-GeH$_3$ E = -4866.393319620797 au
ZPE = 0.04576775 au
G_{corr} = 0.00114999 au

Po	-3.89081027485945	0.42682672604719	-0.91681276038463
Po	0.48846806258040	0.98601430542305	3.93351534165676
Po	-0.69971089706132	-0.67083241932237	1.84264176947588
Ge	-2.65990725949377	1.08316070245947	1.37129632101265
Ge	1.85501583099848	2.45333529170971	2.15801978627002
H	-3.74820600693159	1.03265302412730	2.46340308976022
H	-2.06398280690573	2.50216722495302	1.30211761624376
H	2.86972468472092	1.56428613825224	1.42058966922561
H	2.59090145408240	3.57016662859542	2.92493070400947
H	0.87203253228980	3.06881955543818	1.14843597442407
H	-2.44834411942005	0.83687740231665	-1.82127720169395

H$_3$C-CH$_3$ E = -79.675369592908 au
ZPE = 0.07435934 au
G_{corr} = 0.05123769 au

C	-2.77870850117516	0.80552524215195	-0.86830150761919
H	-2.81541897424387	-0.27542781083275	-0.71609018993229
H	-2.13260361170994	0.99500948529412	-1.72830537359187
H	-3.78561977737273	1.13480637376088	-1.13415377554715
C	-2.26978300579346	1.52527062937182	0.37840007043734
H	-1.26288157135680	1.19596150435740	0.64425568964839
H	-2.91587588319738	1.33581693313113	1.23842126935622
H	-2.23306200515065	2.60622209276544	0.22618166724855

H$_3$Si-SiH$_3$ E = -581.690308462365 au
ZPE = 0.04885772 au
G_{corr} = 0.02210501 au

Si	-2.91514427754730	0.61223487369499	-1.20313354149073
H	-2.95618486162431	-0.85292543005087	-0.97164727892840
H	-2.02229969255476	0.88468182170365	-2.35656222153853
H	-4.28313207640490	1.07632475808139	-1.54294287463996
Si	-2.13286499544877	1.71848160827762	0.71309799974023
H	-0.76514099962215	1.25474070524883	1.05394598821767
H	-3.02666975356026	1.44587362516819	1.86583872430900
H	-2.09251667323752	3.18377248787620	0.48181105433072

H_3Ge-GeH_3 E = -4154.803074056857 au
ZPE = 0.04577789 au
G_{corr} = 0.01654257 au

Ge	-2.93078747522831	0.59033902533199	-1.24033546925177
H	-2.97388788490560	-0.93022452883309	-1.00287435385240
H	-2.00537732961004	0.87189159596689	-2.43824630317519
H	-4.34985491676344	1.07033431669539	-1.59584973158421
Ge	-2.11762274791401	1.74053579517707	0.75037009848615
H	-0.69857810781088	1.26056140857856	1.10596592348203
H	-3.04312901988053	1.45858726929870	1.94812424408043
H	-2.07471584788714	3.26115956778445	0.51325344181498

H_3Sn-SnH_3 E = -430.883943912504 au
ZPE = 0.03963749 au
G_{corr} = 0.00829025 au

Sn	-2.98929387504611	0.50757737041589	-1.38378678973083
H	-3.04093747127511	-1.18596084704116	-1.12955618219282
H	-1.96567586110096	0.81473625512223	-2.72298198632634
H	-4.56859033257948	1.03497410133187	-1.78761513436377
Sn	-2.05918830838548	1.82322507363426	0.89388319904202
H	-0.47987379181525	1.29586769959850	1.29769189089771
H	-3.08279507750606	1.51599298497065	2.23307076094980
H	-2.00759861229149	3.51677181196772	0.63970209172426

H_2N-NH_2 E = -111.702524914538 au
ZPE = 0.05309611 au
G_{corr} = 0.03049958 au

N	-1.57917135678299	0.75299239899349	0.05566100288137
N	-0.74740600427163	1.72185868043926	0.69997957856963
H	-1.67855546029076	1.02256377987755	-0.91447338512089
H	-2.50539136845638	0.70002674299654	0.46874882804696
H	-0.25086476166276	1.25791427589725	1.44959985145792
H	-1.28094699853547	2.48909936179590	1.09746091416501

H_2P-PH_2 E = -684.196307863232 au
ZPE = 0.03475019 au
G_{corr} = 0.00847765 au

P	-3.03385810768963	0.81811098436012	-1.18724002110749
H	-1.81854815002785	0.42696254817582	-1.81140106300754
H	-3.26665761957671	-0.49241504716688	-0.68942011872212
P	-2.01462936534590	1.51268560376457	0.69734218069517
H	-1.78183175904845	2.82321199270009	0.19952251035458
H	-3.22994021831145	1.90383246816627	1.32150253178740

H_2As-AsH_2 E = -4471.319736765365 au
ZPE = 0.03095985 au
G_{corr} = 0.00216928 au

As	-3.08858164736278	0.78429361571388	-1.28490869443976
H	-1.76099250885902	0.36032983548162	-1.91317429646056
H	-3.30340042807048	-0.61923660204171	-0.71869480261506
As	-1.95987890788129	1.54649915135023	0.79506015258087
H	-1.74510373257466	2.95001617669838	0.22877877386425
H	-3.28750799525174	1.97048637279759	1.42324488707026

H_2Sb-SbH_2 E = -481.580991965782 au
ZPE = 0.02651153 au
G_{corr} = -0.00422877au

Sb	-3.17189036841812	0.71921656132623	-1.44610079796851
H	-1.65853212290700	0.24940229060252	-2.10878781366127
H	-3.38406274642925	-0.84634272035068	-0.77232697532149
Sb	-1.87659812226973	1.61157919765993	0.95620328767360
H	-1.66442499767074	3.17713841442832	0.28242980472182
H	-3.38995686230513	2.08139480633366	1.61888851455585

H_2Bi-BiH_2 E = -430.301833895994 au
ZPE = 0.02430663 au
G_{corr} = -0.00810864au

Bi	-3.22183400624169	0.71793113124164	-1.51174485507580
H	-1.63393000405688	0.21674058216952	-2.19874283870606
H	-3.43279891278613	-0.92551418058101	-0.80515753886302
Bi	-1.82664757134741	1.61286933193296	1.02184172483988
H	-1.61569936156267	3.25632124188404	0.31526430369605
H	-3.41455536400519	2.11404044335283	1.70884522410895

HO-OH E = -151.364468696187 au
ZPE = 0.02631192 au
G_{corr} = 0.00400349 au

O	-1.32191119456447	0.80172507791373	-0.10462147832254
O	-0.02917563814148	1.45059571771957	0.00932251261720
H	-1.91096408843687	1.54476463623099	0.09111873180097
H	0.29067032114282	1.36938650813572	-0.90091644609563

HS-SH E = -796.662122835873 au
ZPE = 0.01820350 au
G_{corr} = -0.00669006 au

S	-1.56185062540301	0.55788233643781	0.06610276129330
S	0.32617759016550	1.41093371998378	0.14476050066339
H	-2.29334913458445	1.69005236781314	0.06366637878398

H	0.55764156982195	1.50760351576528	-1.17962632074067

HSe-SeH E = -4801.444734004313 au
ZPE = 0.01559016 au
G_{corr} = -0.01199994 au

Se	-1.66960085167037	0.45117116405094	0.07645040139792
Se	0.46086733836874	1.45369367048820	0.20358694974756
H	-2.44607967354143	1.70724240413278	0.06776893997900
H	0.68343258684307	1.55436470132810	-1.25290297112449

HTe-TeH E = -536.090723680227 au
ZPE = 0.01264889 au
G_{corr} = -0.01661004 au

Te	-0.08312960078957	1.00885460167786	-0.00006347391848
H	-0.25620756786835	2.66300629418818	0.00001927341123
Te	2.69100880548095	1.30175151724227	-0.00009184491557
H	2.86408596317695	-0.35240035310831	0.00031135542282

HPo-PoH E = -475.529275305455 au
ZPE = 0.01151278 au
G_{corr} = -0.01929083 au

Po	-0.17090613870318	1.01401083034646	-0.00185635444026
H	-0.31948406314730	2.75963106674503	0.00180229576641
Po	2.77878441001733	1.29659473485919	-0.00188372792249
H	2.92736339183314	-0.44902457195068	0.00211309659633

H_3C-SiH_3 E = -330.690171439932 au
ZPE = 0.06082648 au
G_{corr} = 0.03594480 au

C	-2.97046107185737	0.53438837308236	-1.33798917020515
H	-2.14676079112275	0.29691802847432	-2.01416741785775
H	-3.68526550614142	1.15524365709019	-1.88176601525324
H	-3.46888732590411	-0.39995979353836	-1.07208888401034
Si	-2.34481165893715	1.41921565591406	0.19467513697603
H	-1.37348464239395	0.57056821756561	0.92881486706422
H	-3.46861235678981	1.73855256979164	1.10998024438624
H	-1.66902269685344	2.68833898162019	-0.17324123110000

H_3C-GeH_3 E = -2117.237323291783 au
ZPE = 0.05913900 au
G_{corr} = 0.03286944 au

C	-2.98864202616876	0.50874420629158	-1.38247482700314
H	-2.15792044626315	0.27704294405536	-2.04885236167883

H	-3.70054979671375	1.13788841801150	-1.91651300439848
H	-3.48389430205975	-0.42148117724485	-1.10444533996543
Ge	-2.33323069966976	1.43567782192691	0.22329185582943
H	-1.32518141997178	0.54901034137726	0.98026405548278
H	-3.50494127667180	1.76412791404180	1.16909859097941
H	-1.63294608248125	2.75225522154043	-0.16615143924574

H_3C-SnH_3 E = -255.269784599058 au
ZPE = 0.05556713 au
G_{corr} = 0.02806139 au

C	-3.02811367593856	0.45291642836121	-1.47925569182568
H	-2.19355375020027	0.22401533830963	-2.14046501275262
H	-3.73845946030996	1.08671753798632	-2.00817459598998
H	-3.52194067428560	-0.47507492831831	-1.19447081606718
Sn	-2.30855603730830	1.47057682518115	0.28352240561868
H	-1.18819654609489	0.47830723354096	1.12007880298982
H	-3.61724670308550	1.83212006718116	1.33051699998692
H	-1.53123920277690	2.93368718775788	-0.15753456195994

H_3C-NH_2 E = -95.699461446501 au
ZPE = 0.06369220 au
G_{corr} = 0.04075147 au

C	-2.96796889867972	0.50399505456170	-1.34809620838034
H	-2.18229939079476	0.39496335523046	-2.09802610203715
H	-3.75856527938071	1.10955937164886	-1.79508944197169
H	-3.38157523750441	-0.49412661720983	-1.14558458011361
N	-2.42726572393823	1.20234542644340	-0.18204122898166
H	-1.68111064910010	0.66605777202095	0.24453575086939
H	-3.14162364060207	1.32824652730445	0.52518512061506

H_3C-PH_2 E = -381.932154816660 au
ZPE = 0.05418982 au
G_{corr} = 0.02964627 au

C	-3.01039870331485	0.47965701954839	-1.50657426379475
H	-2.25346670932923	0.37132285125402	-2.28453026203332
H	-3.86247420454307	1.00099243724189	-1.94486808648580
H	-3.32873292086283	-0.50931487957023	-1.18112716947017
P	-2.31105053120960	1.52705320836747	-0.13448216446669
H	-1.44433523136698	0.55227139067508	0.42688138201288
H	-3.32995051937343	1.28905886248337	0.82558387423786

H_3C-AsH_2 E = -2275.489138493700 au
ZPE = 0.05201628 au
G_{corr} = 0.02606665 au

C	-3.02733168098466	0.46549259282588	-1.55908068492884
H	-2.26992031899047	0.36162489361706	-2.33504859840987
H	-3.89310560826497	0.97169996849085	-1.98429637682743
H	-3.31954328878441	-0.51805669965073	-1.20015250632335
As	-2.28381163010436	1.60748079570675	-0.10553655684016
H	-1.36585689337624	0.53237177738639	0.47491737052668
H	-3.38083939949487	1.29042756162380	0.91008066280298

H_3C-SbH_2 E = -280.613967968892 au
ZPE = 0.04929687 au
G_{corr} = 0.02223554 au

C	-3.05401317850113	0.44492914260197	-1.63975525290967
H	-2.30387059115415	0.34595707813427	-2.42288064516454
H	-3.94061548272957	0.92154528371107	-2.05517865761081
H	-3.31123947569698	-0.53596020273922	-1.24885375677832
Sb	-2.24943515039502	1.72359518620649	-0.06101231893525
H	-1.20787360944785	0.50683568940123	0.55965526208060
H	-3.47336133207527	1.30413871268418	1.06890867931799

H_3C-BiH_2 E = -254.967294049697 au
ZPE = 0.04792243 au
G_{corr} = 0.01988026 au

C	-3.06759817871509	0.43298702445355	-1.67915031862816
H	-2.31453656285730	0.33802092112681	-2.45906181943475
H	-3.96149262462336	0.90300637014815	-2.08459323951398
H	-3.30828923699442	-0.54234216203344	-1.26653813809050
Bi	-2.23057589787237	1.78418366362768	-0.03763087341678
H	-1.14605636253083	0.49208200253262	0.59423039231004
H	-3.51185995640661	1.30310307014462	1.13362730677415

H_3C-OH E = -115.555024441570 au
ZPE = 0.05097202 au
G_{corr} = 0.02818660 au

C	-2.96566472636700	0.51120322574892	-1.34800306034900
H	-2.14879956606425	0.28297834847378	-2.04223974328178
H	-3.67225217396563	1.16787822882431	-1.85423623307559
H	-3.48315244007462	-0.42053412820310	-1.09226238162649
O	-2.51165879041035	1.20668730103439	-0.19492012513187
H	-1.88134264311815	0.65044383412169	0.27364604346472

H_3C-SH E = -438.164945800220 au
ZPE = 0.04580922 au
G_{corr} = 0.02156302 au

C	-3.03155632407541	0.48043432135294	-1.46292908364794

H	-2.18951735225760	0.26302503435491	-2.11719523042564
H	-3.74335537894806	1.10294479336173	-2.00305536579258
H	-3.52738587716596	-0.44165403724488	-1.16585778317556
S	-2.50944970526367	1.45107334218046	-0.00965843982079
H	-1.66160570228930	0.54283335599483	0.50068040286250

H_3C-SeH E = -2440.551328815357 au
ZPE = 0.04429356 au
G_{corr} = 0.01859015 au

C	-3.05818612419544	0.46645114706535	-1.51093784805935
H	-2.20224747772215	0.25488537931018	-2.14551933029846
H	-3.77043844935831	1.08396175853928	-2.05389514528953
H	-3.54233591558601	-0.45191958850167	-1.19114760304317
Se	-2.50479856890767	1.53097657864790	0.05338529836172
H	-1.58486380423041	0.51430153493896	0.59009912832879

H_3C-TeH E = -307.870066630438 au
ZPE = 0.04266407 au
G_{corr} = 0.01590915 au

C	-3.09341273343899	0.45084834512249	-1.57165708908947
H	-2.22556092559218	0.24155560691893	-2.18928423060550
H	-3.80702541942032	1.05490466593721	-2.12768333848307
H	-3.56759785269218	-0.46561425252029	-1.23459485000550
Te	-2.50183423267477	1.64083654937091	0.14026369366909
H	-1.46743917618154	0.47612589517076	0.72494031451446

H_3C-PoH E = -277.582741141336 au
ZPE = 0.04181511 au
G_{corr} = 0.01416874 au

C	-3.11156628348295	0.43988513611603	-1.60492356783863
H	-2.23421403185733	0.23507966152117	-2.20914889259499
H	-3.82434454779550	1.04470867976087	-2.16034166170581
H	-3.57943159776621	-0.47335031164746	-1.25318463124056
Po	-2.49968242559755	1.69491739787823	0.18420859108368
H	-1.41363145350045	0.45741624637116	0.78537466229631

H_3Si-GeH_3 E = -2368.246733369245 au
ZPE = 0.04735732 au
G_{corr} = 0.01934075 au

Si	-3.04395102527719	0.43017694396878	-1.51782606053089
H	-1.91192433780677	0.11700568251737	-2.42300496445822
H	-4.01229873657772	1.28887406104631	-2.24165592021111
H	-3.71709578633378	-0.83492024279643	-1.13671792950598
Ge	-2.24694141298475	1.55752202570337	0.43377281693268

H	-1.23866845234247	0.67912031135590	1.19826828373812
H	-3.41131697329672	1.89004973691218	1.38581015518564
H	-1.54510932538058	2.87543717129251	0.05557114884977

H_3Si-SnH_3 E = -506.284511390243 au
ZPE = 0.04422341 au
G_{corr} = 0.01509777 au

Si	-3.08330702518285	0.37434307684634	-1.61411961381428
H	-1.95183511536725	0.06147369766743	-2.51999581735237
H	-4.05165519875933	1.23264923418944	-2.33830896378891
H	-3.75655095489066	-0.89110045452608	-1.23472253911621
Sn	-2.22419979372050	1.58975925953926	0.49031942170080
H	-1.10181741512076	0.61688294340012	1.34450098222178
H	-3.51561089864003	1.96285964853929	1.55282075390023
H	-1.44232964831860	3.05639828434420	0.07372330624896

H_3Si-NH_2 E = -346.745572194968 au
ZPE = 0.05011711 au
G_{corr} = 0.02529343 au

Si	-3.00696752442670	0.44686012361091	-1.41465201609300
H	-1.87564134537345	0.27558282953847	-2.35616359065630
H	-4.02391841346236	1.36214036239282	-1.98345982020466
H	-3.65250796218522	-0.88569661928689	-1.25133751112970
N	-2.39352917568242	1.16676205592558	0.02659339008497
H	-1.56249159902044	0.80105764870003	0.46453095320717
H	-3.02535279984939	1.54433448911907	0.71537190479152

H_3Si-PH_2 E = -632.950209171619 au
ZPE = 0.04199433 au
G_{corr} = 0.01572222 au

Si	-3.03430897557244	0.44696947073521	-1.58790914964194
H	-1.96553766080635	0.26134481455592	-2.59789709680736
H	-4.13739397631716	1.23152973695142	-2.19167317248366
H	-3.55252311072714	-0.88203205834337	-1.18484219914959
P	-2.17182936737789	1.65840101057583	0.12691634124391
H	-1.40736086991331	0.57947281799663	0.64443197287415
H	-3.27145485928567	1.41535509752834	0.99185661396451

H_3Si-AsH_2 E = -2526.509870454804 au
ZPE = 0.04012806 au
G_{corr} = 0.01251965 au

Si	-3.04684313763327	0.43704846776766	-1.62982982122657
H	-1.98719347290195	0.25592384163100	-2.65020793017812
H	-4.16644353756340	1.19921508417940	-2.23163926206218

H	-3.54038733222835	-0.89313266549522	-1.20275593136141
As	-2.14146905256942	1.73490181586955	0.15424791959698
H	-1.33571243751524	0.55488163334672	0.69137508768669
H	-3.32235984958832	1.42220271270088	1.06969324754462

H_3Si-SbH_2 E = -531.634088089302 au
ZPE = 0.03776710 au
G_{corr} = 0.00906380 au

Si	-3.07418228439503	0.41561485110858	-1.71398948126315
H	-2.02872089035572	0.24139357319652	-2.75136257234860
H	-4.21980570440342	1.14130272153433	-2.31433255752748
H	-3.53081857597932	-0.91780208541965	-1.25787569835914
Sb	-2.09652269547693	1.85657605041171	0.21377432988019
H	-1.17849723411107	0.52713083019960	0.78934805274232
H	-3.41186143527846	1.44682494896890	1.23532123687588

H_3Si-BiH_2 E = -505.990475573502 au
ZPE = 0.03656960 au
G_{corr} = 0.00692402 au

Si	-3.08235279381896	0.41023943937597	-1.74482329708316
H	-2.04708200214212	0.23470252640840	-2.79331505396501
H	-4.24125824483793	1.11517359761772	-2.34641890795323
H	-3.52126911646107	-0.92332632254077	-1.27218638775504
Bi	-2.07370942467358	1.91773557021030	0.23893636074372
H	-1.12307004199331	0.50835334862264	0.82443318433200
H	-3.45166719607299	1.44816273030572	1.29425741168073

H_3Si-OH E = -366.622892978998 au
ZPE = 0.03836161 au
G_{corr} = 0.01392509 au

O	-0.53972731005642	0.83743385458934	0.99116732365710
H	-0.11055294310236	1.27737971816145	0.25355208926319
Si	-2.15378302566132	1.09464108390994	1.23831919266676
H	-2.44854146923222	2.52843553691611	1.48702196851024
H	-2.96303078292734	0.65758932908976	0.07271629453346
H	-2.51151730902032	0.29473626733339	2.42650835136924

H_3Si-SH E = -689.204945675257 au
ZPE = 0.03395569 au
G_{corr} = 0.00812325 au

Si	-3.04918797004259	0.47327115051066	-1.49358604074464
H	-1.92038752425880	0.16016843747771	-2.39908394305041
H	-4.00673381111819	1.37173859357916	-2.17218293889314
H	-3.72207628208282	-0.79008139176767	-1.11498087604817

S	-2.39613030550759	1.57205357517537	0.23643506262650
H	-1.56835444699000	0.61150644502477	0.68538323610987

H_3Si-SeH E = -2691.589160607925 au
ZPE = 0.03264280 au
G_{corr} = 0.00541340 au

Si	-3.07365665189744	0.46006781036138	-1.53547794949042
H	-1.93526627739041	0.14893326336378	-2.42842285500963
H	-4.03090750088740	1.35114500290529	-2.22538183123959
H	-3.74342918800274	-0.80141423668808	-1.14802722002623
Se	-2.39419464989032	1.65102432215508	0.30777383193542
H	-1.48541607193167	0.58890064790255	0.77152052383045

H_3Si-TeH E = -558.903528626007 au
ZPE = 0.03129403 au
G_{corr} = 0.00304838 au

Te	0.05786496511456	0.55933836541740	1.15139480959921
H	0.22399268859366	1.51818710002084	-0.19808688614599
Si	-2.39485230330677	1.09084473451457	1.33294781389713
H	-2.61887777434907	2.53740979106381	1.54962217157916
H	-3.13004405010224	0.65806940734702	0.12385413667485
H	-2.86523636595011	0.32636639163634	2.50955317439563

H_3Si-PoH E = -528.616820987103 au
ZPE = 0.03059222 au
G_{corr} = 0.00148626 au

Po	0.12332547270960	0.53057289433620	1.16891864341042
H	0.27540089443719	1.54236136030595	-0.25323904055469
Si	-2.42228123690040	1.09088903794329	1.34370785297537
H	-2.64455221393419	2.53802637108170	1.55940572963854
H	-3.15380340857227	0.65897175951945	0.13188757696720
H	-2.90524234773990	0.32939436681338	2.51860445756315

H_3Ge-SnH_3 E = -2292.842137641837 au
ZPE = 0.04265579 au
G_{corr} = 0.01234838 au

Ge	-3.08496240032609	0.37213971348022	-1.61744714741295
H	-1.91691435073292	0.04380904514462	-2.56549157013095
H	-4.09215217936142	1.25533010152340	-2.37677767495981
H	-3.78519654448404	-0.94458274907943	-1.23482530258306
Sn	-2.21282657410691	1.60588866055851	0.51826281402685
H	-1.09240449276094	0.62548554565117	1.36546741369281
H	-3.51056181861108	1.97540905100900	1.57353940096805
H	-1.43228768961657	3.06978632171250	0.09148959639907

H_3Ge-NH_2 E = -2133.279740008566 au
ZPE = 0.04841779 au
G_{corr} = 0.02238265 au

Ge	-3.01324763330493	0.44851836002843	-1.45594071537248
H	-1.83634935903467	0.26465906116271	-2.42811327525610
H	-4.07372742438102	1.39634335505771	-2.03989911163191
H	-3.66600210800998	-0.93216974873022	-1.19385654108477
N	-2.33197519290132	1.27764695095865	0.05683139875299
H	-1.58716749393514	0.76086703735146	0.50702839399020
H	-3.03193960843291	1.49517587417125	0.75483316060207

H_3Ge-PH_2 E = -2419.502921791040 au
ZPE = 0.04032545 au
G_{corr} = 0.01272143 au

Ge	-3.04053154396784	0.44141098935334	-1.60838124796806
H	-1.93084707201399	0.25051455475720	-2.65773727434783
H	-4.18591840644173	1.25693872841465	-2.23435774613189
H	-3.57644865618341	-0.93860124392098	-1.18467855949780
P	-2.15298499446429	1.67968993460878	0.17097425478344
H	-1.39825939617533	0.59277818113339	0.68546112144115
H	-3.25541875075336	1.42830974565358	1.02960276172102

H_3Ge-AsH_2 E = -4313.064060265152 au
ZPE = 0.03844742 au
G_{corr} = 0.00955519 au

Ge	-3.05220311472405	0.43208903835861	-1.64762872970332
H	-1.95293829563837	0.24681663764611	-2.70970425826375
H	-4.21614359779768	1.22351794978387	-2.27156569671630
H	-3.56243689825284	-0.95064607049196	-1.20430749522219
As	-2.12284432272678	1.75355258975370	0.19537153882860
H	-1.32667754513792	0.56726221795283	0.73301383223182
H	-3.30716504572230	1.43844852699682	1.10570411884515

H_3Ge-SbH_2 E = -2318.190629281029 au
ZPE = 0.03615644 au
G_{corr} = 0.00621910 au

Ge	-3.07744913253518	0.41416394739257	-1.72434713088391
H	-1.99465280477550	0.23188461039022	-2.80531264359004
H	-4.26636360254820	1.16515327419574	-2.35381353642741
H	-3.55397303998048	-0.97300515678151	-1.25898681138048
Sb	-2.08311049822991	1.87243153519959	0.24906855809059
H	-1.16721382846155	0.54137783495052	0.82418888496610
H	-3.39764591346912	1.45903484465286	1.27008598922516

H_3Ge-BiH_2 E = -2292.547970137613 au
ZPE = 0.03497102 au
G_{corr} = 0.00411623 au

Ge	-3.08581155147401	0.40948859864053	-1.75378827051301
H	-2.01197920978373	0.22377848581133	-2.84505715801080
H	-4.28720273091454	1.13915251988175	-2.38808171786888
H	-3.54731014664959	-0.97846937646101	-1.27560932976189
Bi	-2.06307941160800	1.93206171095488	0.27294176926679
H	-1.10841633394962	0.52569304754015	0.85834189713585
H	-3.43660943562045	1.45933590363235	1.33213611975193

H_3Ge-OH E = -2153.149379899454 au
ZPE = 0.03626289 au
G_{corr} = 0.01041235 au

O	-0.44580470825151	0.81471720852338	0.99017162725681
H	-0.09639877044862	1.28901579882532	0.22931075107421
Ge	-2.19896585772713	1.09871367754755	1.25002241147335
H	-2.45656005964966	2.59891173538760	1.50066342286470
H	-2.99596343778723	0.63318613284272	0.01388987527524
H	-2.53346000613582	0.25567123687341	2.48522713205568

H_3Ge-SH E = -2475.751889424720 au
ZPE = 0.03210437 au
G_{corr} = 0.00489453 au

Ge	-3.06156859675141	0.46459732913440	-1.51903881821089
H	-1.87563410658146	0.15073872130629	-2.44689478020495
H	-4.05478894363640	1.40792935691073	-2.21049914187446
H	-3.75602682798140	-0.84273232052324	-1.10128069726738
S	-2.36558629871115	1.59609775317845	0.29081442123403
H	-1.54926556633816	0.62202596999337	0.72888351632367

H_3Ge-SeH E = -4478.139214943628 au
ZPE = 0.03083532 au
G_{corr} = 0.00228509 au

Ge	-3.08668585027905	0.45217234236527	-1.55468727008268
H	-1.88758380966978	0.13735135983714	-2.46370825250984
H	-4.07324304431854	1.38801284456409	-2.26711951476412
H	-3.78630812784084	-0.85232241502223	-1.13908011644538
Se	-2.37802123810605	1.66615866174267	0.36653459287213
H	-1.45102826978571	0.60728401651306	0.80004506092990

H_3Ge-TeH E = -2345.456786155954 au

ZPE = 0.02957515 au
G_{corr} = 0.00004609 au

Te	0.11256556689999	0.55918297299179	1.13936843070838
H	0.25916167192579	1.51382713551945	-0.21495921862353
Ge	-2.40877279957240	1.09076192525242	1.33969481560784
H	-2.63181459555887	2.59462124814936	1.56739066262542
H	-3.16091912667449	0.63742536456698	0.07803074500506
H	-2.89737355701999	0.29439714351998	2.55975978467682

H_3Ge-PoH E = -2315.171449170985 au
ZPE = 0.02890320 au
G_{corr} = -0.00145515 au

Po	0.17357656293013	0.52719775970678	1.15435223005679
H	0.31447817807340	1.54273883852270	-0.26595826432896
Ge	-2.43419898230777	1.08993278132570	1.34862073041482
H	-2.65646007355633	2.59447925715432	1.57457296745964
H	-3.18888452154401	0.63638909564062	0.08820502051589
H	-2.93566400359539	0.29947805764983	2.56949253588180

Si_2P E = -921.990265122100 au
ZPE = 0.04300612 au
G_{corr} = 0.01462388 au

Si	-3.11978012446018	0.82324604558955	-0.10986022994840
Si	-0.87948112073955	1.18980107158091	-0.08294751873334
H	-3.73216618781155	0.06240825712459	-1.21827293170628
H	-3.90529723436972	0.75870433954364	1.13897120789823
H	-0.03073839410158	0.66861956271505	-1.17400726234461
H	-0.14372333941593	1.37453153148244	1.18408019728166
P	-2.29859405594109	2.88006404370699	-0.72389508749162
H	-2.39208726316029	3.35111524825676	0.61480582504436

H_3Si-SiH_2-PH-SiH_2-SiH_3 E = -1503.727023047850 au
ZPE = 0.09404230 au
G_{corr} = 0.05661625 au

Si	-4.27616589698859	1.52970663825687	0.37074448101564
Si	-0.15809151221031	-0.12966551313756	3.70399286481331
H	-3.85877464469084	2.92424171847067	0.66950258780454
H	-5.48934087501532	1.23342711125390	1.17370630692522
H	0.92338893030186	0.14257341547958	2.72474949740184
H	-0.34213467141902	-1.59714084906552	3.81335502049381
H	0.25311845524740	0.40469451875304	5.02636545540541
Si	-4.74260841869616	1.29286984683606	-1.91622303396144
Si	-2.14972282847053	0.87285031194415	2.97468770962886
H	-3.50407630828060	1.51811149157654	-2.70228982463584
H	-5.76750662792350	2.27342527594802	-2.35363007003215

H	-5.23828844941282	-0.07738472365849	-2.19221255735533
H	-2.00074089808398	2.35087597207853	2.93981585505477
H	-3.25775066032939	0.54658233611000	3.90752734861434
P	-2.72007027226682	-0.00884763121963	0.96513405560345
H	-1.65555688176122	0.60151057037378	0.24979050322352

H_2P-$(SiH_2)_2$-PH- SiH_3 E = -1554.986771892824 au
ZPE = 0.08721368 au
G_{corr} = 0.05008505 au

Si	-4.37334444609751	1.50032091563160	0.40039383764325
Si	-0.05252774686333	0.09399115159476	3.52974465473071
H	-4.00410871131456	2.93937305557163	0.40876344370031
H	-5.56952746357833	1.32535833747023	1.26130735919279
H	0.81631401418625	0.43166762714547	2.37700934766257
H	-0.19427469113079	-1.37876068249600	3.61226561265824
H	0.58143170854546	0.59175779308240	4.77401152851292
Si	-2.59831792862340	0.24100212363711	1.27037684375768
H	-1.43156701050662	0.31829730931861	0.35154387926319
H	-2.99300254411927	-1.18533777424374	1.38146903318269
P	-4.89659356488854	0.71570254200505	-1.66945437619430
H	-5.65188133224575	1.85713741237582	-2.04763160526475
H	-3.69995240988939	1.19518715449481	-2.26423245168228
P	-2.13687797331372	0.96240438075320	3.37013584976772
H	-1.70746723016040	2.25498273365900	2.96697816306918

Si_2S E = -978.260555848699 au
ZPE = 0.03584816 au
G_{corr} = 0.00798190 au

Si	-3.04708789426201	0.92315626210328	0.04678582573440
Si	-0.79803893039377	0.98712885708776	0.10742319439900
H	-3.80273028351955	0.80728216409161	-1.21782536856290
H	-3.85884451502415	0.49082932877332	1.20340645928654
H	0.02974408555832	0.91542120182210	-1.11461483775079
H	-0.02641642734193	0.59889863402969	1.30643115780536
S	-1.98118165501687	2.78925686209223	0.31543407908838

H_3Si-SiH_2-S-SiH_2-SiH_3 E = -1559.992128882924 au
ZPE = 0.08628245 au
G_{corr} = 0.04901185 au

Si	-4.14318958226001	1.63983410606474	0.36744001801562
Si	-0.27501541295562	-0.25883791977777	3.66164689313346
H	-3.74736092586610	3.06376542553538	0.50528191795271
H	-5.27012934594367	1.39475447357121	1.30190161529632
H	0.91182104838872	-0.01298569818545	2.80805406097667
H	-0.60974742646566	-1.70252160587847	3.62635071168206
H	0.04412278129082	0.13008772894138	5.05805801324045

Si	-4.75158133571787	1.14116180623095	-1.84219433464706
Si	-2.09293716583576	1.00224189332838	2.88275376529579
H	-3.61035783100602	1.40484810067270	-2.75086396795985
H	-5.90067228987244	1.98751093517942	-2.24999736243358
H	-5.13974019105390	-0.28555381355808	-1.94913700498252
H	-1.78544697535741	2.45420626631054	2.90475466984101
H	-3.29557038066682	0.78542586322591	3.72536861510740
S	-2.48074516667821	0.36013112833913	0.86197392948150

HS-$(SiH_2)_2$-S- SiH_3 E = -1667.506123761277 au
ZPE = 0.07127860 au
G_{corr} = 0.03404105 au

Si	-4.18122618775611	1.65353306782791	0.30716181213759
Si	-0.24097418162281	0.00369408985303	3.60450280691997
H	-3.81007771497106	3.08408850126400	0.18977656419360
H	-5.33627430876018	1.54845656514206	1.23071399008015
H	0.86897535977358	0.17292337225835	2.63954069320365
H	-0.63132799764415	-1.42404791227522	3.63967411778206
H	0.18055481600225	0.45023462734613	4.94951498732718
Si	-2.37057395672498	0.40938858354662	1.12614393418390
H	-1.19792871863524	0.52132524631840	0.22467234307150
H	-2.72155155995937	-1.02703167307787	1.24286936211993
S	-4.64721241599657	0.80921371164101	-1.62272743378242
H	-5.66477052079228	1.64666594225550	-1.89295561127941
S	-1.91638679291301	1.23752252790009	3.06259381404228

Ge_2N E = -4208.849462412401 au
ZPE = 0.04515743 au
G_{corr} = 0.01580039 au

N	0.64482600916899	2.04035719164302	0.00713799202978
H	0.71076217238288	2.76051628672118	-0.70068784481336
Ge	-0.56563715998520	0.59043560177429	-0.10365101509980
Ge	1.78458048582536	0.53053234733104	0.05493331770389
H	-1.24279476896526	0.34314092576450	-1.46512666168213
H	-1.57170733946113	0.56824943253974	1.05760022826781
H	2.62507697711689	0.24543744618610	-1.20426733645962
H	2.62375810391745	0.46264518804011	1.34036625005342

H_2N-$(GeH_2)_2$-NH-GeH_3 E = -6342.197772468074 au
ZPE = 0.09601923 au
G_{corr} = 0.05808321 au

N	0.14864382609138	-0.22004225373710	0.46602847837987
H	0.65655940678972	0.63814098594220	0.63964256620953
H	-0.39396206858373	-0.10122944262442	-0.38021198510577
Ge	1.21798518849411	-1.74382949333190	0.47688231977800
H	1.94948716423781	-1.72843692377957	1.83244189457286

H	0.26467003324489	-2.95137584465551	0.40130730282227
Ge	2.80814152434994	-1.80065029254619	-1.39585207258846
H	2.09767729038800	-1.45885726485357	-2.72673156948031
H	3.92469781930480	-0.73711644402352	-1.19437163191240
N	3.49758725890194	-3.51302846733281	-1.59299113117254
H	3.95367945933428	-3.66874038029748	-2.48233107629378
Ge	4.16341331521241	-4.54287980522113	-0.21452771602942
H	5.58161808679850	-4.11999739298761	0.23840422095419
H	4.20271892640856	-6.01057144166990	-0.67797902319774
H	3.19036396902725	-4.36154258888142	0.96405777306371

H_3Ge-GeH_2-NH- GeH_2-GeH_3 E = -8363.722185097449 au
ZPE = 0.09318170 au
G_{corr} = 0.05268435 au

N	0.06195212092963	-1.19058124735964	0.56639526287937
H	-0.05668618061006	-0.79899284014030	1.49136260152624
Ge	0.89787401546940	-0.04574136776342	-0.62835026342431
Ge	-0.35122283460874	2.05140945006170	-0.63790424466221
Ge	0.22560596686922	-3.03694296465769	0.55104141539422
Ge	-1.69812427531587	-3.96410937974156	1.73455095052093
H	0.37329127721127	3.13000021096918	-1.46548551295722
H	-1.75818622120434	1.83952977355371	-1.22398208322404
H	-0.50040904509690	2.57083304050387	0.80577290504539
H	2.38571733224158	0.20444058222845	-0.26558793144950
H	0.85111055425948	-0.76079138315969	-1.99221982927343
H	0.24708007809249	-3.44020061303885	-0.93576582111356
H	1.54986762236956	-3.51841905792136	1.20102793157027
H	-1.55625769707128	-5.48588637286969	1.92658082953888
H	-1.81185467850508	-3.31096424034719	3.12627788565993
H	-2.99735429503034	-3.68117348031748	0.95990983396901

H_2N-GeH_3 E = -2133.279741976842 au
ZPE = 0.04841892 au
G_{corr} = 0.02238397 au

N	1.04123875284971	1.31455657218282	-0.25366928255287
H	1.04614747525626	1.78079264676774	0.64472999183268
H	1.53583962818532	1.89964902283905	-0.91518519351093
Ge	1.65432779060834	-0.43505224731637	-0.19462066323267
H	3.12846991123422	-0.58444030276223	0.25837577277377
H	0.75119481261584	-1.18783236320532	0.79609238400560
H	1.51185480925029	-1.00233262850567	-1.61651403931558

CSiO E = -404.592342691647 au
ZPE = 0.04678930 au
G_{corr} = 0.02139000 au

Si	-0.06917128292559	-1.14077870930681	-0.00074382000520
C	0.85353670124365	0.41658458915897	-0.00074374586874
H	-0.66619746281465	-1.69716839610080	-1.22917004265214
H	-0.66619718531545	-1.69716827906089	1.22768255971174
H	1.02438026207237	0.98564691460910	0.90892052898411
H	1.02438040589265	0.98564698980829	-0.91040797357693
O	1.57602873184703	-0.89035018910785	-0.00074361659284

H_3C-O-SiH_2-CH_2-OH E = -520.211376478322 au
ZPE = 0.10082054 au
G_{corr} = 0.06896894 au

O	-1.91770040548404	1.04834602808002	-0.44887006283691
O	2.05739098861729	0.81909232894251	0.20059930945385
H	2.83925799965521	1.17143439645252	-0.23659642267310
C	-3.25464754538374	0.72253913145597	-0.11016041296865
Si	-0.59879602851550	0.52596934401257	0.39250655173950
C	0.88853644854149	1.24927741497327	-0.50874015072947
H	-3.42267295208215	-0.35920511783200	-0.14584151759318
H	-3.91564308315787	1.20117026521656	-0.83324644197626
H	-3.51197953920951	1.08435500439664	0.89111264822455
H	-0.54025933675787	-0.95822938956835	0.40540236026792
H	-0.65050456109764	1.00421302779769	1.79784169389937
H	0.89422590589817	0.90021453742460	-1.54907632882028
H	0.80843266897618	2.34343549864798	-0.53252329598734

H_3C-SiH_2-O-CH_2-SiH_3 E = -735.347812684406 au
ZPE = 0.11050911 au
G_{corr} = 0.07727440 au

C	-3.20061434148575	0.45985580196131	0.03096266039945
C	0.87811615047638	1.38468874693543	-0.50684497369426
H	-3.09059224425032	-0.61589003476697	-0.11840109979684
H	-4.16454422041362	0.76416371763172	-0.38433947543858
H	-3.21595512418040	0.65615019040036	1.10469410816662
H	1.05614936241771	1.20242970167902	-1.57458252962766
H	0.92506822457704	2.47136429539592	-0.35873014149937
Si	-1.81543455178712	1.38919306472874	-0.79298009550907
Si	2.20489507302104	0.52489500604431	0.53352722295810
O	-0.39876191857516	0.87511349803369	-0.11302728536247
H	-1.78586847154296	1.13385814150672	-2.25872231795101
H	-1.95628332747911	2.85793504205355	-0.59940439797339
H	3.54016801122637	1.04142585991207	0.13897920285516
H	1.97736239468454	0.80397486733529	1.97022521215663
H	2.15785046331136	-0.93715148885117	0.30124371031670

H_3Si-O-CH_2-SiH_2-OH E = -771.279806946419 au
ZPE = 0.08806946 au

G_{corr} = 0.05502151 au

C	-0.72823054658372	0.53997145084238	0.30189584486291
H	-0.72926536229008	0.80982621317847	1.36492029544853
H	-0.68021971560018	-0.55431110795953	0.24662702720170
Si	-3.42895225503088	0.59855066274551	0.11064481365493
Si	0.74013192964296	1.31835324607462	-0.58359980097338
O	-1.90735973284195	1.03258763808502	-0.35086677305831
O	2.08226160072508	0.73181363190688	0.18700008894856
H	-3.61268437524108	-0.87139451782134	-0.00147914724777
H	-4.35892750869483	1.30483597977697	-0.79344831016091
H	-3.68121749416435	0.98827282215551	1.52188330983725
H	2.95284535339424	1.00722538170917	-0.10837909093494
H	0.65937784605846	2.79651751273151	-0.48897995645301
H	0.72395309062634	0.93626643657483	-2.01685212112556

CGeO E = -2191.119367903585 au
ZPE = 0.04475026 au
G_{corr} = 0.01802086 au

Ge	-0.20534571428946	-1.11407174891639	-0.00074094598279
C	0.93502174096142	0.42188463003539	-0.00074391917632
H	-0.67777797559579	-1.76219892617446	-1.30119056555601
H	-0.67778050734506	-1.76220646320429	1.29970275637494
H	1.04092023281605	1.00690360885406	0.91167701565711
H	1.04091814634360	1.00690057797950	-0.91316739265400
O	1.62080424710925	-0.83479875857381	-0.00074305866293

H_3C-GeH_2-O-CH_2-GeH_3 E = -4308.420681321947 au
ZPE = 0.10646676 au
G_{corr} = 0.07087100 au

C	-3.30941124075578	0.44218462911384	0.04201857092674
C	0.87329425699302	1.38316508069995	-0.50904579731097
H	-3.16684812791018	-0.62647955087677	-0.11460143153002
H	-4.28330241710708	0.73159374611595	-0.35387954242494
H	-3.28574198025061	0.64978877599872	1.11123673908046
H	1.05872705541089	1.20003475048180	-1.57537888639990
H	0.91844431361097	2.46861268148377	-0.35144880168781
Ge	-1.88805773372967	1.43298582266932	-0.85115945351576
Ge	2.30185613896963	0.51536185466456	0.55509765245266
O	-0.36868279978473	0.84106884120049	-0.08985826000826
H	-1.80140896510100	1.14673248750691	-2.36872236008246
H	-1.96828245298783	2.96054575486179	-0.62166031449814
H	3.67931800563021	1.07218258781512	0.13611101697480
H	2.07600028223944	0.79840661948228	2.05083869781139
H	2.27565114477271	-1.00417767121774	0.31305197021223

H_3C-O-GeH_2-CH_2-OH E = -2306.736119141128 au

ZPE = 0.09858758 au
G_{corr} = 0.06575111 au

O	-1.98619016355375	1.08470392089489	-0.51722225670323
O	2.13317043421320	0.84250895455578	0.17580333252801
H	2.90864544556913	1.20373823658899	-0.26675741439560
C	-3.29574852576294	0.72786756059517	-0.11813575685340
Ge	-0.58207170809804	0.49365403596265	0.43133695210810
C	0.95981229267553	1.26231173719077	-0.52158991229307
H	-3.44983424694076	-0.35859726374613	-0.13607829893088
H	-3.99860199233567	1.18342985037187	-0.81868545031791
H	-3.53233184967627	1.09195465532261	0.88981605479408
H	-0.58190908412626	-1.05070587764677	0.41496568579674
H	-0.70270304376438	1.01309497747344	1.88081913725099
H	0.94680842713905	0.90623449283317	-1.55705126089939
H	0.85659457466119	2.35241718960353	-0.53481288208442

H_3Ge-O-CH_2-GeH_2-OH E = -4344.331819242763 au
ZPE = 0.08361648 au
G_{corr} = 0.04826907 au

C	-0.77916139968402	0.55254542332887	0.24734966808185
H	-0.76430679282282	0.81675598508087	1.31161293663524
H	-0.71381504687729	-0.53967724402077	0.17520310668298
Ge	-3.55332556737152	0.56352651596507	0.16436550777976
Ge	0.78838704232779	1.35595062777331	-0.63486492741694
O	-1.92978657592141	1.06344005193139	-0.41554008003138
O	2.18266416159442	0.70288727018328	0.29900749753707
H	-3.67571656342182	-0.96782634364437	0.02895365128910
H	-4.53584526902770	1.30836594138480	-0.74586779941137
H	-3.69423844854665	0.97813193235992	1.64307924239116
H	3.03881291445053	0.98583016843675	-0.03544652815528
H	0.76775171370066	2.89639839951492	-0.53588712349950
H	0.90029266159984	0.92218662170595	-2.11259897188269

CGeAlE = -2358.579785065143 au
ZPE = 0.04584079 au
G_{corr} = 0.01688367 au

Ge	0.09770911889087	0.68235861652747	0.03222515638362
C	1.31782067438524	2.35478408302811	-0.01954829994594
H	-0.80774314965281	0.58595072212653	-1.21219637963743
H	-0.77989965800244	0.63642313479723	1.29919218345930
H	1.23107384149118	2.99868152578273	0.85618776089223
H	1.20500963691301	2.95750960626853	-0.92125879645668
Al	2.47192994887415	0.80181660031015	-0.00141950212598
H	3.96845058710076	0.30248371115923	-0.00684012256912

H_3C-AlH-GeH_2-CH_2-AlH_2 E = -2641.670902088353 au

ZPE = 0.09728162 au
G_{corr} = 0.06059415 au

C	-3.79290529831851	-0.32387285354468	-1.12768851058243
H	-3.88213772275388	-1.39388192212287	-1.34141609390404
H	-3.47184494386987	0.17952965740884	-2.04093736311791
H	-4.80283614248458	0.02692793754992	-0.88827988758286
C	-0.39228758248338	2.79865105080097	1.80630545197248
H	-0.43690491165885	3.82415282815495	1.44447847399510
Ge	-0.67690042633263	1.54647129011080	0.27528335747993
Al	-2.60086075110165	-0.02321773160252	0.39080735442065
H	-2.84987444991417	-0.74575255006045	1.77752374412399
Al	1.37283837219084	2.03952819428566	2.16263639566717
H	2.63951858447660	2.63990520241478	1.43016908907778
H	1.49536884941171	0.84377668702678	3.19186906678484
H	-1.15088152279500	2.64273737476893	2.57113398928177
H	-0.42297650810305	2.28380415630967	-1.05894404271645
H	0.65371820373648	0.66847506849915	0.41864381509994

H_3C-GeH_2-AlH-CH_2-GeH_3 E = -4475.888503473131 au
ZPE = 0.10824876 au
G_{corr} = 0.07038471 au

Ge	2.71812520867427	-3.34257437385326	-0.37119691061924
H	4.05306527669287	-3.14855660528565	-1.11799563282371
H	2.73075271512932	-4.73206601768178	0.30078744099338
H	2.61406927579169	-2.27585525188782	0.73838098745736
C	1.18237898248040	-3.19338256352776	-1.60341930917331
Al	-0.53774354637200	-3.45995966390254	-0.72365342107199
H	-1.14024238517820	-4.92584145967160	-0.63825193834304
Ge	-1.71031776385150	-1.52521340417641	0.30896358565932
C	-3.22490989226508	-1.99278786626474	1.49856221871537
H	1.24885351930319	-2.19907804818116	-2.05314214763807
H	1.34177040478365	-3.93454920731255	-2.39139125291066
H	-0.72055974271149	-0.64951853079174	1.12112758220631
H	-2.25880816777861	-0.58012164120319	-0.79409646402345
H	-2.87532700764939	-2.59713223148054	2.33566136314957
H	-3.69046625360266	-1.08868251911966	1.89022838941287
H	-3.97375222344644	-2.56364082565950	0.94962982900927

CGeGa E = -4040.043877458781 au
ZPE = 0.04557625 au
G_{corr} = 0.01554504 au

Ge	0.08525620781676	0.71853021785588	0.03117558684110
C	1.27678383938558	2.37061212407206	-0.02018876962374
H	-0.80018208794705	0.55744257036034	-1.21992884476806
H	-0.76927161015137	0.61117880942293	1.30925931361181
H	1.25267661477529	3.01108919508597	0.86019924637145
H	1.22812442717797	2.97244702969321	-0.92639975873062

Ga	2.47400760944408	0.77794453421097	-0.00062144933653
H	3.95695599949872	0.30076351929861	-0.00715332436541

H_3Ge-GaH-CH_2-GeH_2-GaH_2 E = -8042.169465766182 au
ZPE = 0.08426188 au
G_{corr} = 0.04292911 au

Ge	-0.67569986312055	-0.33195144665065	-0.01630020966685
H	-0.27705679436808	0.93852834810511	0.77182170983818
C	-0.16794351149468	-1.92639176249689	1.06163582736441
H	-0.63252458354608	-2.79727469552361	0.59854908432967
Ge	2.84776468065801	-3.13454310995143	-0.92841788840972
H	0.21598976766345	-0.31625219329559	-1.29081718095431
H	-0.56719667197713	-1.79711325752252	2.06769739468977
H	3.83725118342426	-2.19806634706850	-1.65605408974438
H	1.80728735576313	-3.59313246242888	-1.97412747983559
H	3.65430119286421	-4.38953626239780	-0.52370814209033
Ga	-3.03589328841489	-0.34114184687869	-0.71870637183241
H	-3.74974659990014	-1.72208833623726	-0.97942875137789
H	-3.85063390113970	0.99166150170881	-0.92191379375489
Ga	1.80091795571926	-2.05585412866343	1.01957180297857
H	2.66861407786891	-1.37179220069855	2.14424265846578

H_3C-GaH-GeH_2-CH_2-GaH_2 E = -6004.601518524712 au
ZPE = 0.09708891 au
G_{corr} = 0.05850618 au

C	-3.89456129609762	-0.07584510731949	-1.30118491062648
H	-4.19172510739384	-1.10697605294583	-1.50902782800731
H	-3.37096808123176	0.32341055269156	-2.16858135717509
H	-4.81247548839464	0.49778931689857	-1.14699308332565
C	-0.34102601800287	2.29929147274115	1.89390814050558
H	-0.90070225609425	3.18245960367626	1.58453586682490
Ge	-0.52492440734778	0.93850432287147	0.44685153301277
Ga	-2.79645359133831	-0.01903933833826	0.35009722301068
H	-3.41112375342312	-0.57989368113154	1.69636143398603
Ga	1.57755150732149	2.67248429671069	2.13519532708133
H	2.28826430406072	3.75631902666637	1.24293208801840
H	2.40884877565583	1.81316136431281	3.15819019804736
H	-0.78964804151224	1.88823961105528	2.79852393959278
H	-0.07954873689848	1.59878840439774	-0.88226090563885
H	0.51952594069691	-0.18145940228682	0.71303717469352

H_3C-GeH_2-GaH-CH_2-GeH_3 E = -6157.356815775880 au
ZPE = 0.10807213 au
G_{corr} = 0.06951804 au

Ge	2.71104835580257	-3.33477257015415	-0.37282527597779
H	4.04849912366288	-3.14955840357781	-1.11774965123783

H	2.71799501147634	-4.71800990179297	0.31049754151150
H	2.60427778471258	-2.25673127095434	0.72490346133952
C	1.18534825939455	-3.19236848261503	-1.61430120211084
Ga	-0.55305282357845	-3.44546710390849	-0.69932156985349
H	-1.13992569433023	-4.91040217008027	-0.60741654964154
Ge	-1.69909593205807	-1.51989411731469	0.32139342962156
C	-3.22268239576908	-2.00042696456496	1.48790163399877
H	1.23769037890286	-2.20268965843330	-2.07148680529503
H	1.32327870888605	-3.94692634822356	-2.39079938536539
H	-0.69686420181047	-0.67180720846794	1.14379503701988
H	-2.22123646979629	-0.57831121096591	-0.79449657737309
H	-2.88095079279995	-2.61756370047481	2.31877526494048
H	-3.69134801850201	-1.10173240859279	1.88864347274656
H	-3.96609289419326	-2.56229868987891	0.92268149567671

SiGeAl E = -2609.603378455993 au
ZPE = 0.03724656 au
G_{corr} = 0.00626334 au

Ge	0.07883080725064	0.58558385469330	-0.06967039071098
Si	1.30209934156803	2.70072256034935	0.07402392308658
H	-0.97169332737827	0.40023549799395	-1.18166085238486
H	-0.53147665801999	0.12667167462165	1.27577734089627
H	1.12861412240680	3.75089014923959	1.11153908279794
H	1.26761728115721	3.32159142277133	-1.27979922431884
Al	2.54440701253731	0.63144257237240	-0.01773579997005
H	3.88595242047825	-0.19712973204159	0.11386792060394

H_3Ge-Al**H-SiH_2-GeH_2-**Al**H_2** E = -4930.247562757149 au
ZPE = 0.07576511 au
G_{corr} = 0.03496716 au

Ge	-0.82837369792692	-0.24554151794783	-0.41342025248745
H	-0.14575060808251	1.02851551552139	0.13963615193270
Si	-0.33543900162038	-2.03090701930402	1.14750309791876
H	-0.99545324820343	-3.25564383318451	0.61856856535261
Ge	3.19001520420082	-3.21354961585835	-0.83765092692448
H	-0.07725693408260	-0.59491090245791	-1.72072010586609
H	-0.98381420496442	-1.69715783080702	2.44415363806373
H	4.10507124618642	-2.23212099230835	-1.60486775953667
H	2.17823543510291	-3.77106994021950	-1.86165952733126
H	4.09057785696236	-4.39881105686806	-0.41869140853693
Al	-3.27342117839940	-0.04181201246583	-0.69143371498562
H	-4.01374884699067	-0.93053475016934	-1.76970403687338
H	-4.10209722991045	0.88640516268796	0.28455309796534
Al	2.09604520838001	-2.14335264092154	1.12131555386473
H	2.97084099934824	-1.40445676569681	2.21646219744407

H_3Si-AlH-GeH_2-SiH_2-AlH_2 E = -3143.687833844652 au
ZPE = 0.07726994 au
G_{corr} = 0.03750949 au

Si	-4.06870978881243	0.27126703409990	-1.38761736875640
H	-4.48542576860979	-0.86933041004240	-2.24944805312179
H	-3.31709530349087	1.22537069408869	-2.24375775302383
H	-5.31033985647055	0.95287169560773	-0.93145027607244
Si	-0.41487758656085	2.37624035725605	1.80463060396754
H	-1.26600498209361	3.25283752897516	0.95383159505017
Ge	-0.37774810338195	0.20152174526911	0.73943387703572
Al	-2.72383712955611	-0.53334706528113	0.51017165395288
H	-3.37894468012995	-1.40779809766851	1.65819673843275
Al	1.90678435555664	3.10017568652579	2.00115997783700
H	2.60069902762927	3.86818676362625	0.80389386598893
H	2.75956336616883	2.68439272680341	3.26803142905569
H	-1.12118398774182	2.20948794411555	3.10448615632202
H	0.34803669888346	0.38935579877634	-0.61448000309608
H	0.53011748860978	-0.71399801215199	1.59450239642779

H_3Si-GeH_2-AlH-SiH_2-GeH_3 E = -4977.915651377618 au
ZPE = 0.08751402 au
G_{corr} = 0.04855306 au

Ge	2.25337436873387	-3.67762960423301	0.41017001908092
H	3.79102030301968	-3.62843039913811	0.49459158433724
H	1.80388196356314	-5.12028457909275	0.72031908800273
H	1.67864001261624	-2.76641107637916	1.51198628043978
Si	1.44652773079949	-2.98743761043521	-1.74415019784857
Al	-0.99367221174363	-3.18671842621458	-1.75624237289441
H	-1.71589466661154	-4.43877487333454	-2.40447915792353
Ge	-2.29902079210123	-1.50438752121135	-0.48981360009933
Si	-2.19519083920548	-1.91741191593951	1.87155287183603
H	1.88535502240667	-1.57762173278945	-1.93031125623156
H	2.13546934029648	-3.81506692078006	-2.77017236170286
H	-1.74683611424820	-0.07589714945276	-0.70781364014864
H	-3.79236419691584	-1.46216134689694	-0.88723767899313
H	-0.79208083911925	-1.80165539102103	2.33758551254024
H	-3.03303749072203	-0.95598732996709	2.62879263332910
H	-2.66928319076835	-3.29308433311427	2.16541659627599

SiGeGa E = -4291.074593171485 au
ZPE = 0.03696127 au
G_{corr} = 0.00507425 au

Ge	0.06191096494385	0.60618933802471	-0.10534242788008
Si	1.25668686296381	2.69635944281758	0.10637149254639
H	-1.04226279640605	0.42100701885192	-1.16688392668468
H	-0.44876720426082	0.06931808803936	1.25395217121553
H	1.12860807285782	3.80294126126937	1.09262625816517

H	1.35560440044702	3.25884893144001	-1.26947848455613
Ga	2.55214446320731	0.67664101377831	-0.00312483560740
H	3.84042623624701	-0.21129709422130	0.11822175280120

H_3Ge-Ga**H-SiH$_2$-GeH$_2$-**Ga**H**$_2$ E = -8293.193262994055 au
ZPE = 0.07538082 au
G_{corr} = 0.03306668 au

Ge	-0.81613555821101	-0.24819215804642	-0.43065176087033
H	-0.16675957284596	1.05263707350254	0.09475671756001
Si	-0.32073589021842	-2.00117175182526	1.15316043853884
H	-0.97110576186998	-3.24164694750009	0.65310847418281
Ge	3.16072205324858	-3.20937430945695	-0.82397906710807
H	-0.08398267873613	-0.60545943068199	-1.74486012234861
H	-0.94491816790494	-1.65125793652470	2.45615382828386
H	4.05189352282739	-2.21031929078096	-1.59381129256871
H	2.14041877172448	-3.78264027557644	-1.82884786487443
H	4.07471601045849	-4.37517885488570	-0.38535118295972
Ga	-3.24278034671186	-0.07796307464821	-0.70404702742980
H	-3.97621610723366	-0.98917630588898	-1.75640521075020
H	-4.07964479138100	0.85610333237290	0.24612327838613
Ga	2.08019803438306	-2.14110314577971	1.11677811298603
H	2.96976148247089	-1.42020512427973	2.20191724897222

H_3Si-Ga**H-GeH$_2$-SiH$_2$-**Ga**H**$_2$ E = -6506.634320076876 au
ZPE = 0.07692286 au
G_{corr} = 0.03498316 au

Si	-4.02441170979623	0.29978304415558	-1.38342073609229
H	-4.38550255226842	-0.83855820768477	-2.27005786605894
H	-3.26777666868993	1.28711574961196	-2.19323307700832
H	-5.28757721212637	0.94022238351214	-0.93244747341994
Si	-0.42344564264100	2.35524875719480	1.82539943897590
H	-1.29308605091971	3.23189693805770	0.99621814411523
Ge	-0.39147912208099	0.18174079235719	0.77412897343747
Ga	-2.72751829871406	-0.51452266388073	0.49949708628205
H	-3.39466990507015	-1.39500030046711	1.62695076750265
Ga	1.86103024960705	3.09812630890565	1.94498870488944
H	2.51064760060172	3.87165911659521	0.73716283712594
H	2.76585389381675	2.70464773297455	3.17198431577738
H	-1.07792225840731	2.20204153749354	3.15172826404340
H	0.34112059504700	0.34146813345531	-0.57863968153377
H	0.47577083164171	-0.75863493228109	1.64132514196373

H_3Si-GeH$_2$-GaH-SiH$_2$-GeH$_3$ E = -6659.390837296760 au
ZPE = 0.08728300 au
G_{corr} = 0.04776207 au

Ge	2.25311937722310	-3.68939987392777	0.40289942469578

H	3.79024224466348	-3.62406046785433	0.48545947286237
H	1.81807444062031	-5.14100706312089	0.68767684433452
H	1.66853207540739	-2.79860784247686	1.51580397312205
Si	1.44201649848217	-2.97167014665483	-1.73885504016137
Ga	-0.96601968803715	-3.16466729127353	-1.71142822883410
H	-1.69309925185575	-4.42818122038569	-2.31505701424597
Ge	-2.27755042490256	-1.48161439667629	-0.49178386956635
Si	-2.21431986847655	-1.93432942864470	1.86287563525650
H	1.86965054620845	-1.55964521604104	-1.92232412588605
H	2.10174228777386	-3.79272110454113	-2.78753179319507
H	-1.70000627971004	-0.06311159077254	-0.70152724381046
H	-3.75756821457979	-1.43452204368432	-0.93286629092129
H	-0.81490565162930	-1.85374928745041	2.34718291850734
H	-3.04242390585167	-0.96737120609691	2.62398193279623
H	-2.72059578533592	-3.30430203039861	2.12568772504585

SiGeO E = -2442.194252582257 au
ZPE = 0.03593580 au
G_{corr} = 0.00795826 au

Ge	-0.38497372371758	-1.25196671487735	-0.00074790975947
Si	0.98624857592583	0.56242397994646	-0.00074407807142
H	-0.75397698149740	-2.04247438712448	-1.26793722457795
H	-0.75397088423382	-2.04246178685828	1.26645235176277
H	1.23423666120944	1.36778004813712	1.21596917418822
H	1.23423861958150	1.36778444330669	-1.21745353078060
O	1.51495790273204	-0.99867266253016	-0.00074489276154

H_3Ge-O-SiH$_2$-GeH$_2$-OH E = -4595.413353742042 au
ZPE = 0.07414149 au
G_{corr} = 0.03683193 au

Si	-0.72848210942129	0.38773840577158	0.49383977621213
H	-0.65677675000385	0.76746445601455	1.92926942082775
H	-0.58742887084137	-1.08947398271876	0.40946485613640
Ge	-3.87871595313537	0.58516710539491	0.10108833757680
Ge	1.03214787203325	1.45243515704595	-0.72575954208926
O	-2.13853771070363	0.88694863832659	-0.17983535180101
O	2.53976935458727	0.82098108864162	0.04570770306949
H	-4.13346492435823	-0.92500344280159	-0.04827050359125
H	-4.60940120946465	1.39872332487766	-0.97375996669347
H	-4.22503221127835	1.06720789053979	1.52072130020382
H	3.34103122349582	1.19332571633490	-0.33536406257907
H	1.02309759394308	2.99741317831701	-0.61502419097846
H	1.05350652514733	1.09558781425578	-2.23271159629387

H_3Si-GeH$_2$-O-SiH$_2$-GeH$_3$ E = -4810.511163975829 au
ZPE = 0.08512175 au

G_{corr} = 0.04617801 au

Si	-3.59570183334091	0.27674201850882	0.17549878212436
Si	1.12374769358367	1.59783178843977	-0.69484480446454
H	-3.29268390157050	-1.15532219902805	-0.04781948883177
H	-4.96955711471949	0.57056709219290	-0.29902308130823
H	-3.49412129860209	0.59292935502684	1.61842478308811
H	1.47319423786752	1.32386670611185	-2.11577854987378
H	1.31851782413977	3.05668004927300	-0.47223173433864
Ge	-2.03235228722079	1.61526351065779	-1.04236413959753
Ge	2.55061462505989	0.34534400761816	0.75414707451564
O	-0.42385015580401	1.15826019772009	-0.37750909466130
H	-1.99903126123888	1.32700443953266	-2.55999763095833
H	-2.19939981819657	3.13660998290199	-0.83117315013516
H	4.03120116140322	0.70374198335930	0.51137122493088
H	2.23290537008273	0.63156394803608	2.23464157531109
H	2.38807223855644	-1.16907647035120	0.51925803419921

H_3Si-O-GeH_2-SiH_2-OH

E = -2808.886607091557 au
ZPE = 0.07633177 au
G_{corr} = 0.04049751 au

O	-2.02640033793795	0.93764268343926	-0.25749815121315
O	2.61779156567028	0.86388360435516	0.11689503372076
H	3.46547870306184	1.14975855839052	-0.23347680432544
Si	-3.63672712896630	0.68995878239023	-0.11580808126892
Ge	-0.55736065985557	0.39478423080183	0.62830850727716
Si	1.22689779935963	1.36429312784417	-0.63094019639027
H	-3.96863098268358	-0.75501875692016	-0.22150104648795
H	-4.31665595706365	1.42536604544351	-1.20653164858563
H	-4.14273190865933	1.18092061718405	1.19258654618106
H	-0.57042639252404	-1.14983292994586	0.64300426786742
H	-0.68044398944881	0.90671789579878	2.08037749603852
H	1.16100259954754	0.90005582050478	-2.03986454221846
H	1.10384724949998	2.84408279071371	-0.63314345059509
